# Supplementary material for: CellUntangler: Separating distinct biological signals in single-cell data with deep generative models
Source: Cell Genom. 2025 Dec 1;6(2):101073. doi: 10.1016/j.xgen.2025.101073 (PMC12903416; doi:10.1016/j.xgen.2025.101073)
Supplement: Document S1. Figures S1–S7 and Tables S2–S4 [file mmc1.pdf]

Cell Genomics, Volume *6*

Supplemental information

CellUntangler: Separating distinct biological signals in single-cell data with deep generative models

Sarah Chen, Aviv Regev, Anne Condon, and Jiarui Ding

Supplementary Information

Supplemental Figures

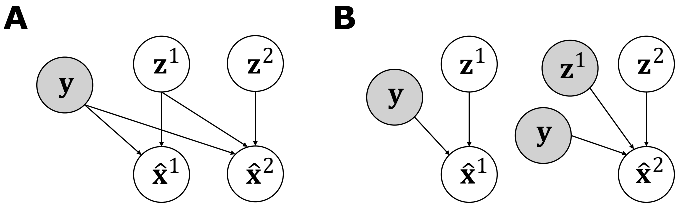

Figure S1: Probabilistic graphical models for CellUntangler, related to Figure 1. The probabilistic graphical model for CellUntangler when the latent representation is decomposed into $\mathbf{z}^1$ and $\mathbf{z}^2$, (**A**) when stop gradient is not used, and (**B**) when stop gradient is used.

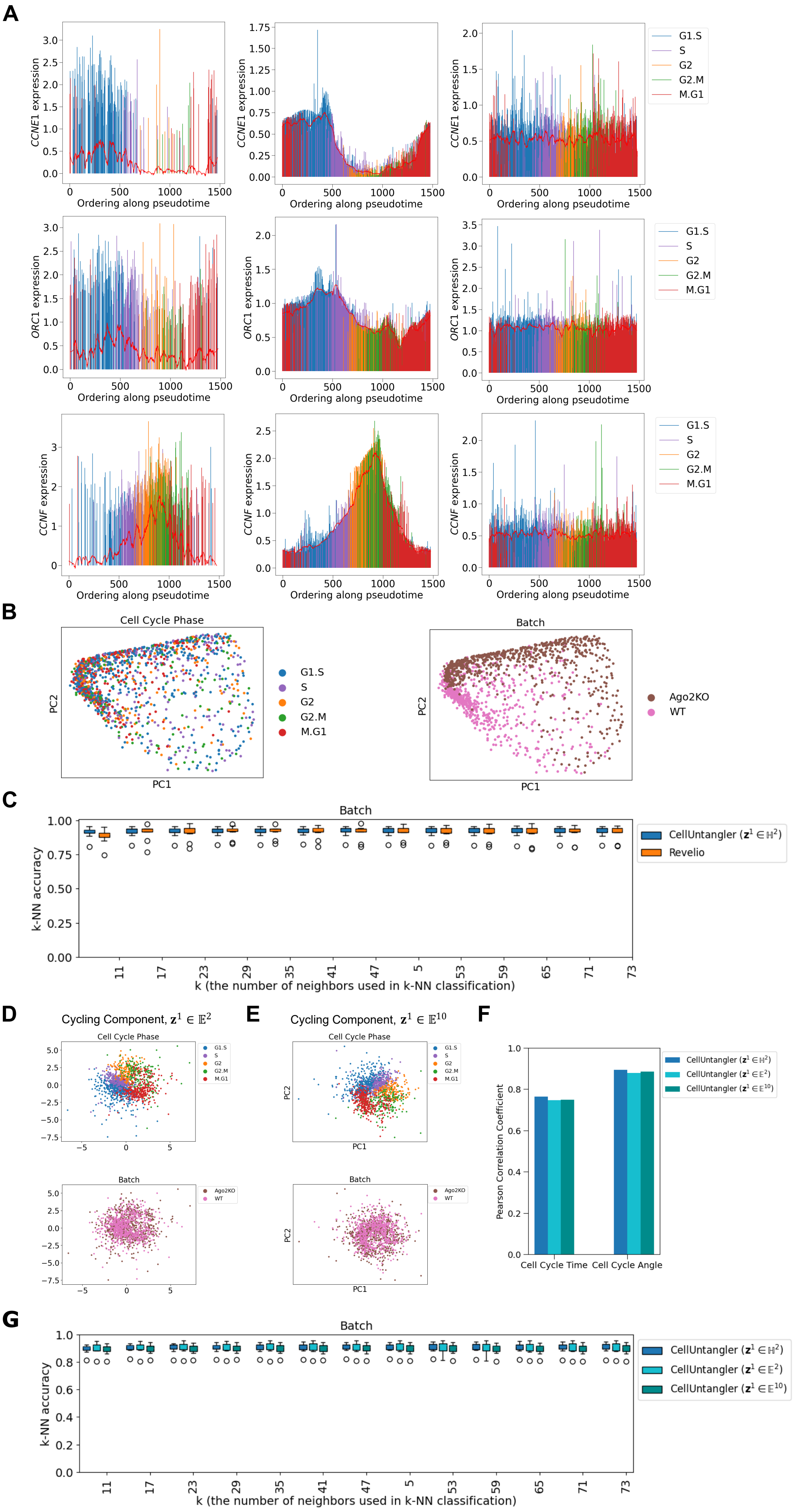
A
CCNE1 expression
ORC1 expression
CCNF expression
Ordering along pseudotime
G1.S
S
G2
G2.M
M.G1
B
Cell Cycle Phase
Batch
PC1
PC2
Ago2KO
WT
C
Batch
k-NN accuracy
k (the number of neighbors used in k-NN classification)
CellUntangler ($\mathbf{z}^1 \in \mathbb{H}^2$)
Revelio
D
Cycling Component, $\mathbf{z}^1 \in \mathbb{E}^2$
E
Cycling Component, $\mathbf{z}^1 \in \mathbb{E}^{10}$
F
Pearson Correlation Coefficient
Cell Cycle Time
Cell Cycle Angle
CellUntangler ($\mathbf{z}^1 \in \mathbb{H}^2$)
CellUntangler ($\mathbf{z}^1 \in \mathbb{E}^2$)
CellUntangler ($\mathbf{z}^1 \in \mathbb{E}^{10}$)
G
Batch
k-NN accuracy
k (the number of neighbors used in k-NN classification)

Figure S2: CellUntangler captures and removes the cell cycle from HeLa cells, related to Figure 2. (**A**) Gene expression of *CCNE1* (G1.S), *ORC1* (G1.S), and *CCNF* (G2) as a function of pseudotime obtained with the embeddings from CellUntangler using the original preprocessed data (size-factor normalized and log1p-transformed, left) and the reconstructed gene expression output from CellUntangler after decoding $\mathbf{z}^1$ and $\mathbf{z}^2$ (right). Cell cycle stages (G1.S, G2) are in parentheses. (**B**) PCA representation of the reconstructed data obtained by running $\mathbf{z}^2$ through CellUntangler's decoder. (**C**) Accuracy of k-nearest neighbors on wild type and knockout cells using $\mathbf{z}^2$ from CellUntangler and PCA embeddings from Revelio, when using the Revelio genes for CellUntangler and Revelio. Boxplots depict the medians and the interquartile ranges (IQR). The whiskers are the lowest datum still within 1.5IQR of the lower quartile and the highest datum still within 1.5IQR of the upper quartile. Individual points below and above the whiskers indicate outliers. (**D**) The first component of CellUntangler when $\mathbf{z}^1 \in \mathbb{E}^2$ and (**E**) when $\mathbf{z}^1 \in \mathbb{E}^{10}$. (**F**) Comparison of cell cycle reconstruction when using the hyperbolic space with the RoWN or the Euclidean space to capture the cell cycle. (**G**) Accuracy of k-nearest neighbors on wild type and knockout cells using $\mathbf{z}^2$ from CellUntangler and either the hyperbolic space with the RoWN or the Euclidean space to capture the cell cycle. Boxplots depict the medians and the interquartile ranges (IQRs). The whiskers are the lowest datum still within 1.5 IQR of the lower quartile and the highest datum still within 1.5 IQR of the upper quartile. Individual points below and above the whiskers indicate outliers.

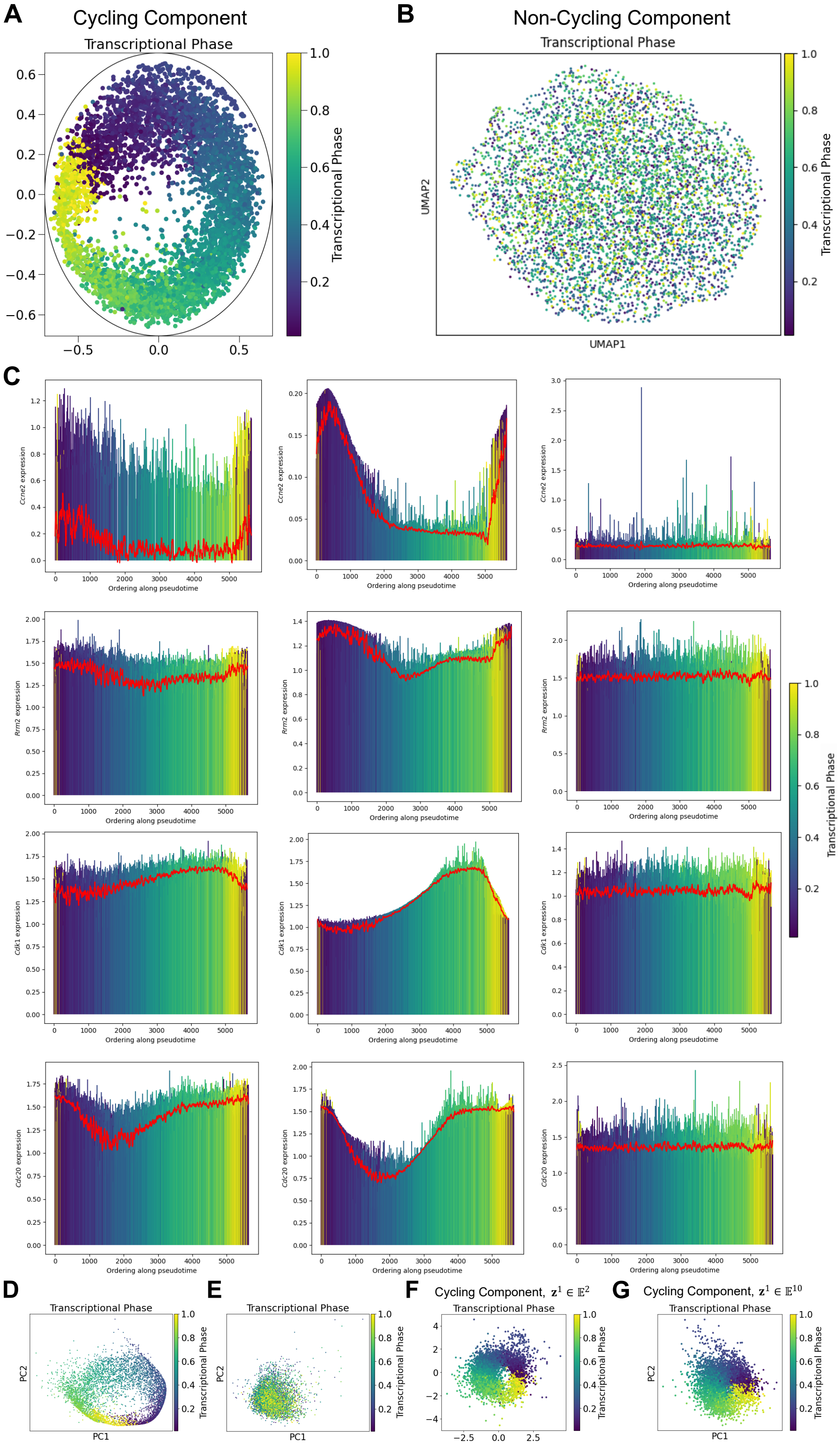
A
Cycling Component
Transcriptional Phase
B
Non-Cycling Component
Transcriptional Phase
UMAP1
UMAP2
C
Ordering along pseudotime
Ccne2 expression
Rrm2 expression
Cdk1 expression
Cdc20 expression
D
E
F
Cycling Component, $\mathbf{z}^1 \in \mathbb{E}^2$
G
Cycling Component, $\mathbf{z}^1 \in \mathbb{E}^{10}$
PC1
PC2

Figure S3: Analysis of the embeddings from CellUntangler on the mESCs demonstrates the capturing and removal of the cell cycle signal, related to Figure 2. (**A**) Visualization of the embeddings for the first component, $\mathbf{z}^1$, where cells were colored by the transcriptional phase output from the original study, and (**B**) embeddings for the second component $\mathbf{z}^2$. (**C**) Marker gene expression across cells when ordered by pseudotime obtained using $\mathbf{z}^1$ and colored using the transcriptional phase from DeepCycle of the original preprocessed data (size-factor normalized and log1p-transformed, left), and the reconstructed gene expression output from CellUntangler after decoding $\mathbf{z}^1$ (middle) and $\mathbf{z}^2$ (right). From top to bottom, *Ccne2* (G1.S), *Rrm2* (S), *Cdk1* (G2), and *Cdc20* (M). Cell cycle stages (G1.S, S, G2, M) are in parentheses. (**D**, **E**) PCA representations of the data reconstructions obtained from $\mathbf{z}^1$ (**A**) and $\mathbf{z}^2$ (**B**), respectively. (**F**) The first component of CellUntangler when $\mathbf{z}^1 \in \mathbb{E}^2$ and (**G**) when $\mathbf{z}^1 \in \mathbb{E}^{10}$.

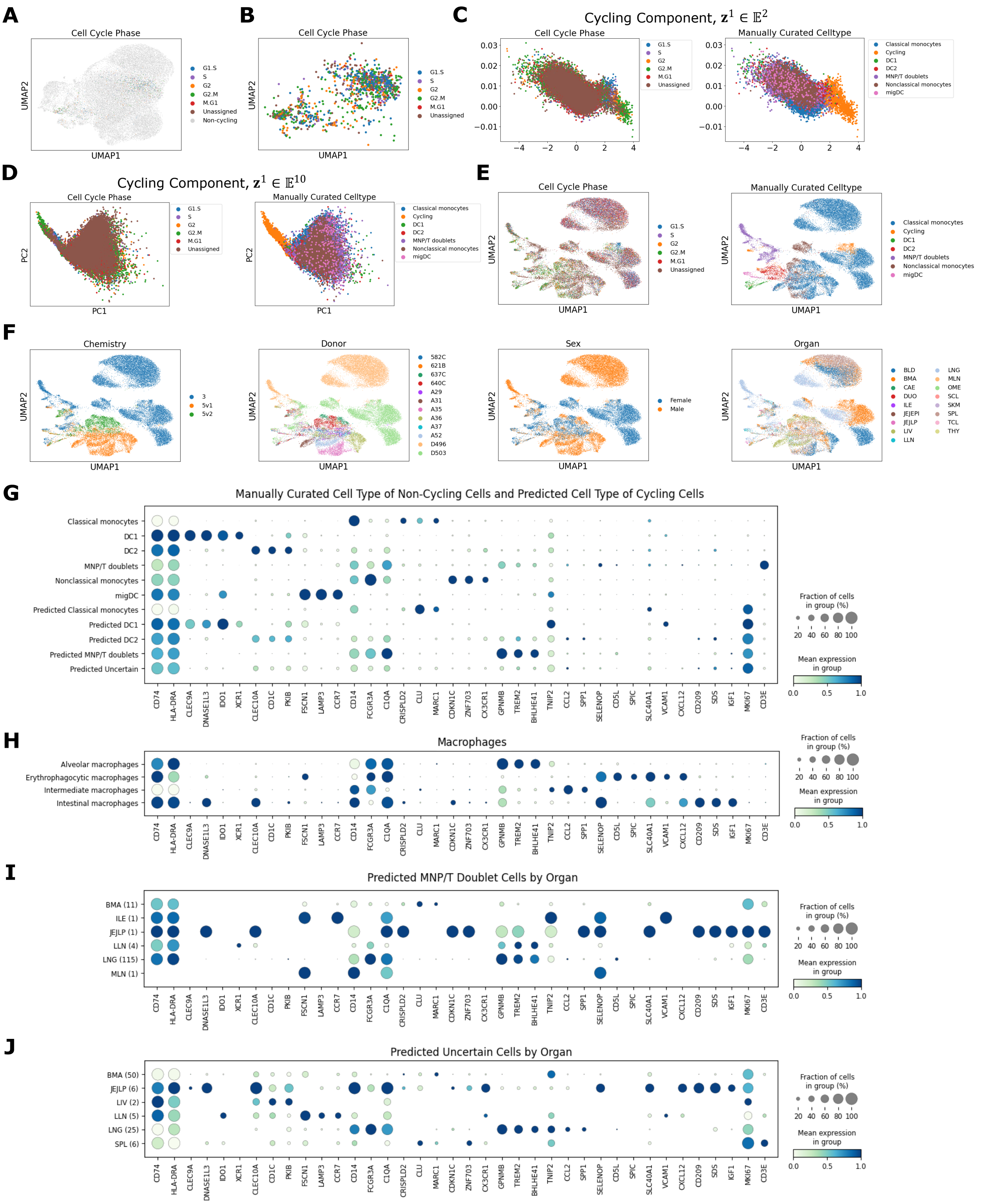
A
B
C
D
E
F
G
H
I
J
Cell Cycle Phase
UMAP1
UMAP2
G1.S
S
G2
G2.M
M.G1
Unassigned
Non-cycling
Cycling Component, $\mathbf{z}^1 \in \mathbb{E}^2$
Manually Curated Celltype
Classical monocytes
Cycling
DC1
DC2
MNP/T doublets
Nonclassical monocytes
migDC
Cycling Component, $\mathbf{z}^1 \in \mathbb{E}^{10}$
PC1
PC2
Chemistry
3
5v1
5v2
Donor
582C
621B
637C
640C
A29
A31
A35
A36
A37
A52
D496
D503
Sex
Female
Male
Organ
BLD
BMA
CAE
DUO
ILE
JEJEPI
JEJLP
LIV
LLN
LNG
MLN
OME
SCL
SKM
SPL
TCL
THY
Manually Curated Cell Type of Non-Cycling Cells and Predicted Cell Type of Cycling Cells
Predicted Classical monocytes
Predicted DC1
Predicted DC2
Predicted MNP/T doublets
Predicted Uncertain
CD74
HLA-DRA
CLEC9A
DNASE1L3
IDO1
XCR1
CLEC10A
CD1C
PKIB
FSCN1
LAMP3
CCR7
CD14
FCGR3A
C1QA
CRISPLD2
CLU
MARC1
CDKN1C
ZNF703
CX3CR1
GPNMB
TREM2
BHLHE41
TNIP2
CCL2
SPP1
SELENOP
CD5L
SPIC
SLC40A1
VCAM1
CXCL12
CD209
SDS
IGF1
MKI67
CD3E
Fraction of cells in group (%)
20 40 60 80 100
Mean expression in group
0.0 0.5 1.0
Macrophages
Alveolar macrophages
Erythrophagocytic macrophages
Intermediate macrophages
Intestinal macrophages
Predicted MNP/T Doublet Cells by Organ
BMA (11)
ILE (1)
JEJLP (1)
LLN (4)
LNG (115)
MLN (1)
Predicted Uncertain Cells by Organ
BMA (50)
JEJLP (6)
LIV (2)
LLN (5)
LNG (25)
SPL (6)

Figure S4: Applying CellUntangler and Scanpy on the immune cells dataset to remove the effects of the cell cycle, related to Figure 3. (**A**) The second component, $\mathbf{z}^2$, of CellUntangler projected to 2D using UMAP when only cycling cells are colored by cell cycle phase while non-cycling cells are in gray. (**B**) The same UMAP as (**A**) but only the cycling cells. (**C**) The first component of CellUntangler when $\mathbf{z}^1 \in \mathbb{E}^2$ and (**D**) when $\mathbf{z}^1 \in \mathbb{E}^{10}$, colored by cell cycle phase (left) and manually curated cell type (right). (**E**) UMAP visualizations colored by cell cycle phase (left) and manually curated cell type (right) after regressing out the cell cycle using Scanpy and performing PCA. (**F**) UMAP visualizations colored by different batches present in the dataset, after regressing out the cell cycle using Scanpy and performing PCA. (**G**) Marker gene expression across cell type of the non-cycling cells and across predicted cell type of the cycling cells. The cell type was considered uncertain if one or more of the *k*-NN classifiers predicted a different cell type from the rest. (**H**) Marker gene expression of the macrophages present in the original dataset of immune cells. (**I**) Marker gene expression of cycling cells predicted to be MNP/T doublets and (**J**) cycling cells whose identity was uncertain. The organs are bone marrow (BMA), ileum (ILE), the lamina propria of the jejunum (JEJLP), lung-draining lymph nodes (LLN), lung (LG), mesenteric lymph nodes (MLN), liver (LIV), and spleen (SPL). The number of cells from each organ is indicated in the parentheses for (**J**) and (**I**).

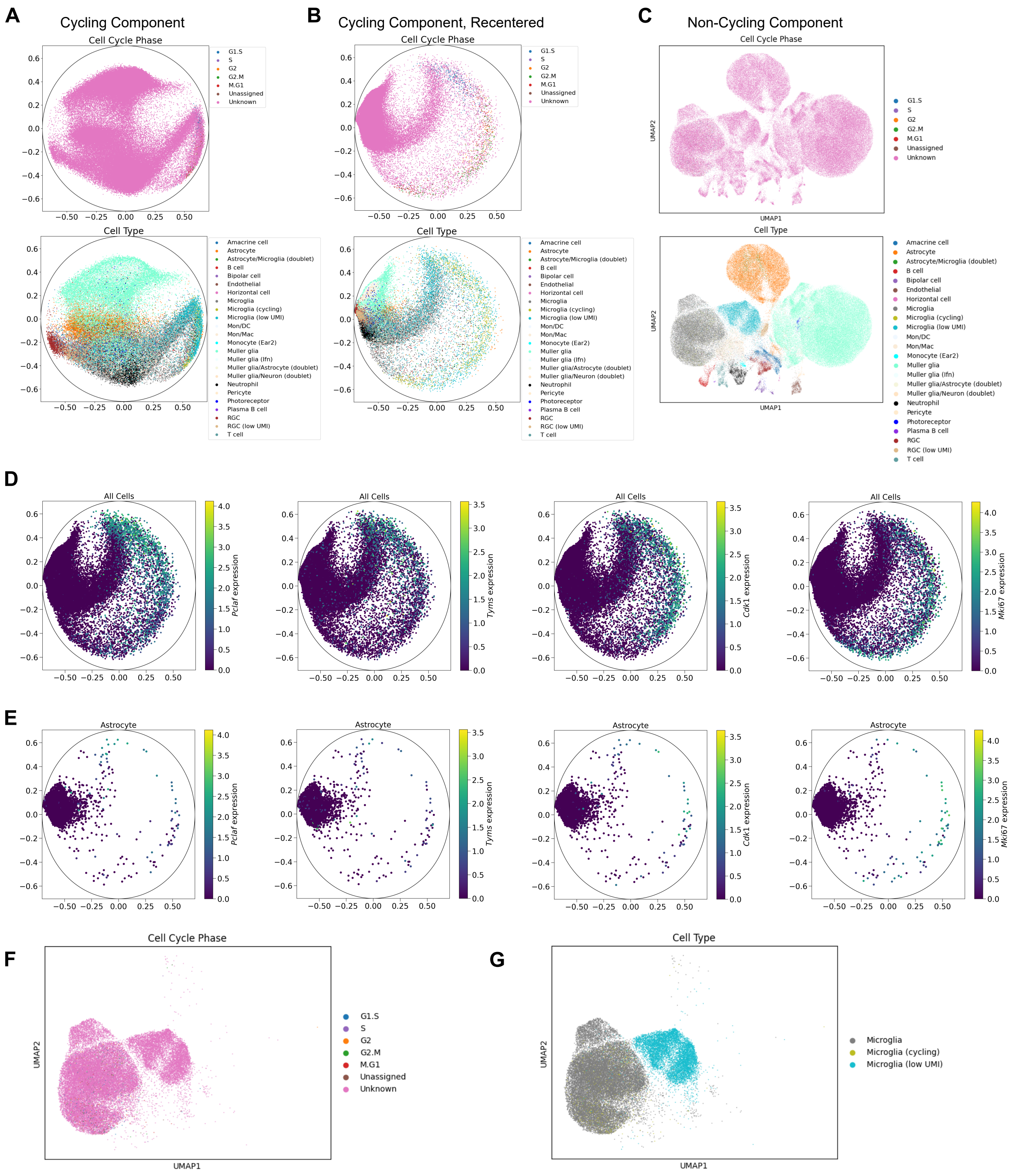
A
Cycling Component
Cell Cycle Phase
Cell Type
B
Cycling Component, Recentered
Cell Cycle Phase
Cell Type
C
Non-Cycling Component
Cell Cycle Phase
Cell Type
UMAP1
UMAP2
G1.S
S
G2
G2.M
M.G1
Unassigned
Unknown
Amacrine cell
Astrocyte
Astrocyte/Microglia (doublet)
B cell
Bipolar cell
Endothelial
Horizontal cell
Microglia
Microglia (cycling)
Microglia (low UMI)
Mon/DC
Mon/Mac
Monocyte (Ear2)
Muller glia
Muller glia (Ifn)
Muller glia/Astrocyte (doublet)
Muller glia/Neuron (doublet)
Neutrophil
Pericyte
Photoreceptor
Plasma B cell
RGC
RGC (low UMI)
T cell
D
All Cells
Pclaf expression
Tyms expression
Cdk1 expression
Mki67 expression
E
Astrocyte
F
Cell Cycle Phase
G
Cell Type
Microglia
Microglia (cycling)
Microglia (low UMI)

Figure S5: Capturing and removing the cell cycle signal in the mouse retinal dataset, related to Figure 3. (**A**) The embeddings, $\mathbf{z}^1$, before recentering and (**B**) after recentering the origin to (-0.5, 0.1). (**C**) The embeddings of the second component, $\mathbf{z}^2$, after projecting to 2D using UMAP. (**D**) The embeddings of the first component, $\mathbf{z}^1$, for all cells and (**E**) only astrocytes colored by *Pclaf* (G1.S), *Tyms* (S), *Cdk1* (G2), and *Mki67* (M) gene expression. Cell cycle stages are in parentheses. (**F**) The projected UMAP with only the microglia colored by cell cycle phase and (**G**) cell state.

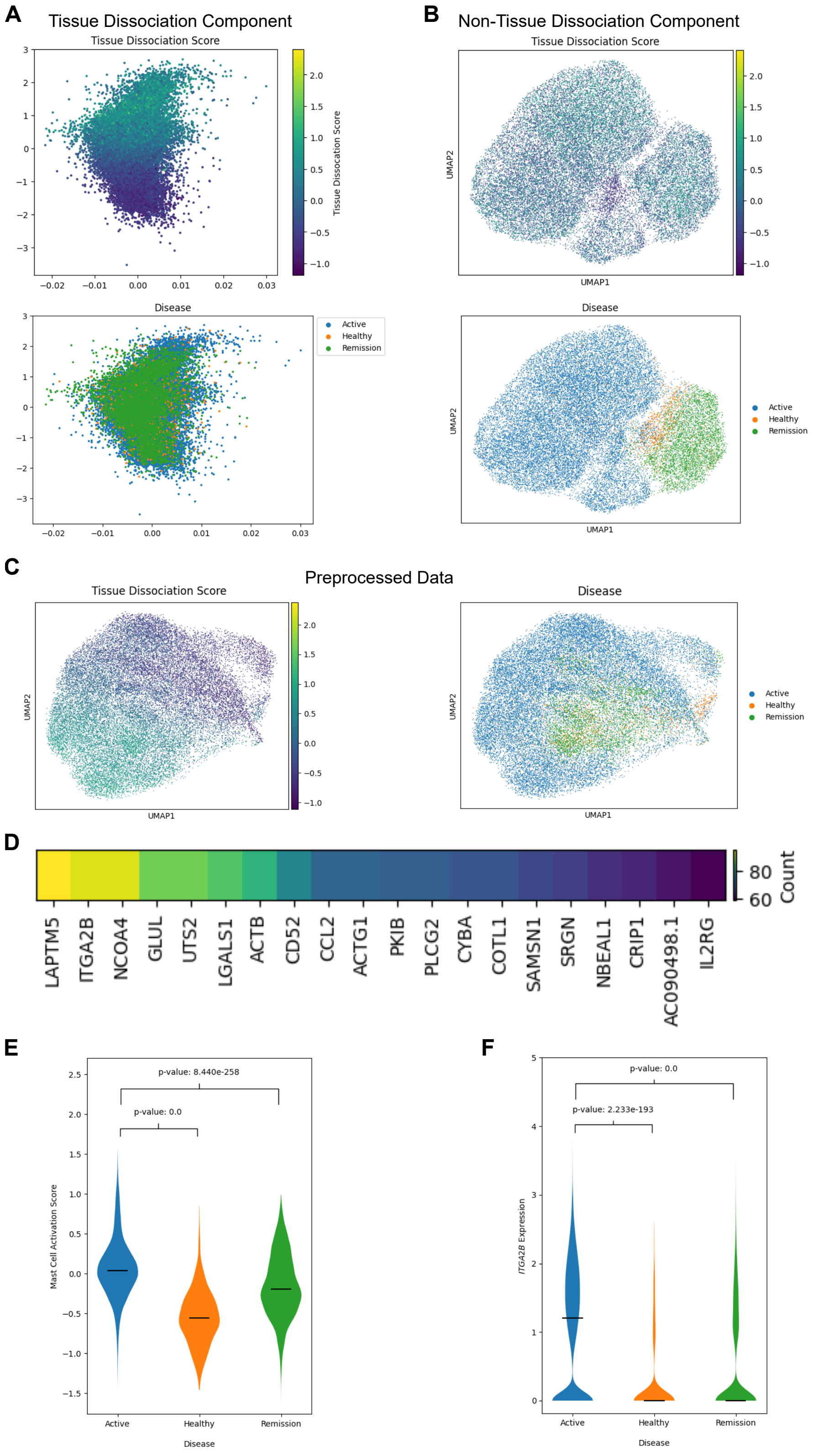

A
Tissue Dissociation Component
Tissue Dissociation Score
Tissue Dissocation Score
Disease
Active
Healthy
Remission
B
Non-Tissue Dissociation Component
Tissue Dissociation Score
UMAP1
UMAP2
Disease
C
Preprocessed Data
Tissue Dissociation Score
Disease
D
Count
LAPTM5
ITGA2B
NCOA4
GLUL
UTS2
LGALS1
ACTB
CD52
CCL2
ACTG1
PKIB
PLCG2
CYBA
COTL1
SAMSN1
SRGN
NBEAL1
CRIP1
AC090498.1
IL2RG
E
p-value: 8.440e-258
p-value: 0.0
Mast Cell Activation Score
Disease
F
p-value: 0.0
p-value: 2.233e-193
ITGA2B Expression

Figure S6: CellUntangler filters the tissue dissociation signal from mast cells, related to Figure 7. (**A**) The embeddings of the first component, $\mathbf{z}^1$, and (**B**) the embeddings of the second component, $\mathbf{z}^2$, after projecting to 2D using UMAP. (**C**) The standard preprocessing pipeline outputs, projected to 2D using UMAP. Batch correction on 10x Genomics chemistry version is performed using Harmony. (**D**) The top 20 genes found that were upregulated in active EoE patients compared to healthy individuals. (**E**) The signature scores computed using the 20 genes from (**D**) and the original preprocessed data (size-factor normalized and log1p-transformed). (**F**) Expression of *ITGA2B* in the original preprocessed data (size-factor normalized and log1p-transformed). Statistical tests were performed using a two-sided Mann-Whitney *U* test between disease statuses (Active and Healthy as well as Active and Remission). Horizontal lines indicate the medians.

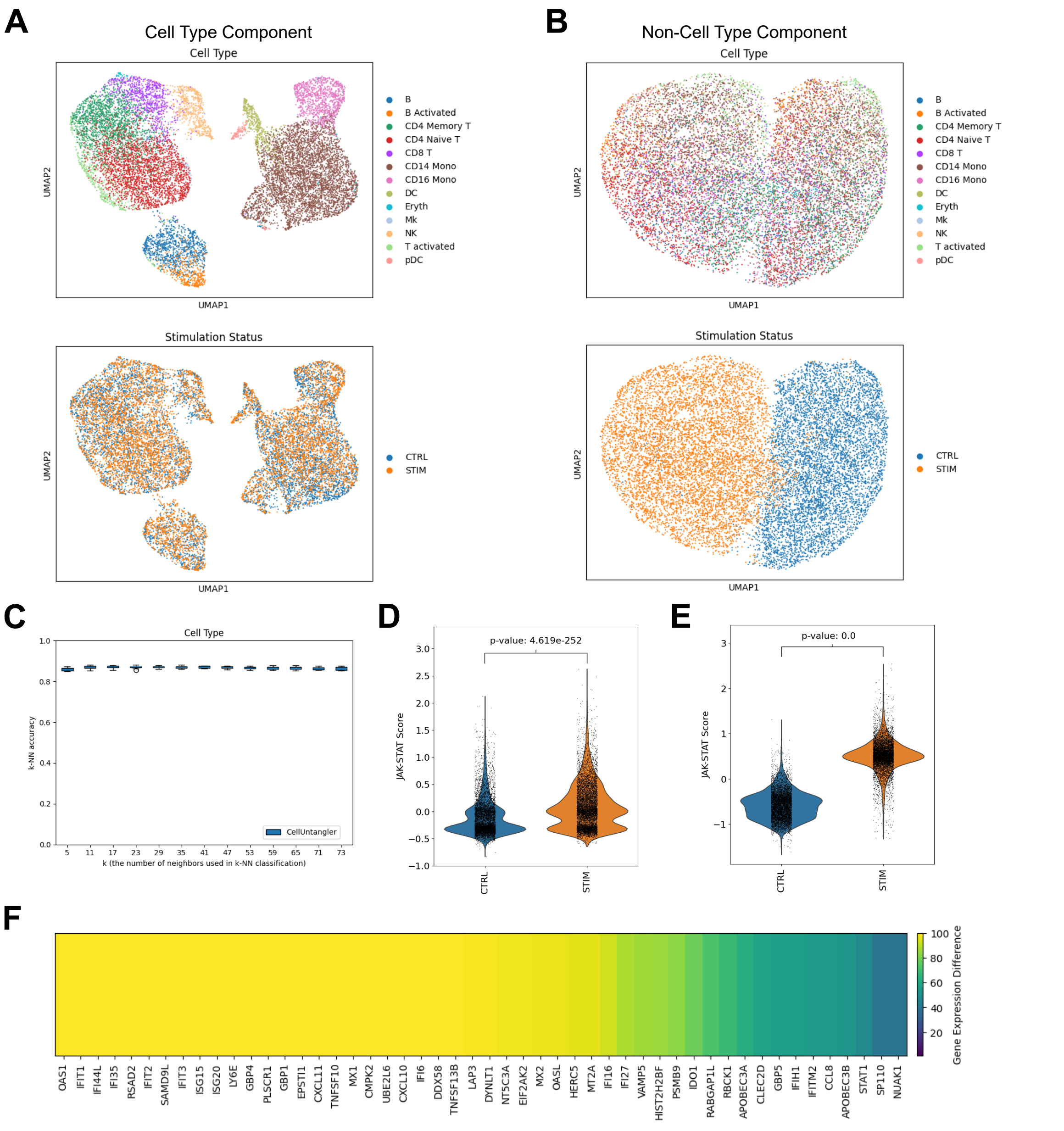

Figure S7: CellUntangler filters cell type to isolate control and stimulation signals, related to Figure 7. (**A**) The embeddings of the first component, $\mathbf{z}^1$, and (**B**) the embeddings of the second component, $\mathbf{z}^2$, both projected to 2D using UMAP. (**C**) The *k*-NN accuracies on cell type using $\mathbf{z}^1$ and 10-fold cross-validation. (**D**) The JAK-STAT scores using the original pre-processed data (size-factor normalized and log1p-transformed), and (**E**) the JAK-STAT scores using the reconstructed gene expression from CellUntangler after decoding $\mathbf{z}^2$. Statistical tests were performed with a two-sided Mann-Whitney *U* test between the control (CTRL) and stimulated (STIM) cells. (**F**) The top 50 upregulated genes in stimulated cells relative to control cells found using the reconstructed gene expression output from CellUntangler after decoding $\mathbf{z}^2$.

Supplemental Tables

	CellUntangler	tricycle	scPrisma	Revelio	Seurat	SiFT	DeepCycle	reCAT	Cyclum	ccRemover	SC1CC
Cell cycle pseudotime or ordering	Yes	Yes	Yes	Yes	No[a]	No	Yes	Yes	Yes	No	Yes
Filter cell cycle	Yes	No	Yes	Yes	Yes	Yes	No	No	Yes	Yes	No
Applicable to non-cyclic signals	Yes	No	Yes	No	Yes	Yes	No	No	No	No	No

Table S2: Scope of application for CellUntangler and other methods, related to Figure 2. We compared the cell cycle pseudotime and cell cycle filtering of CellUntangler with representative and well-maintained models (tricycle, scPrisma, Revelio, Seurat, and SiFT). DeepCycle was not used in our experiments due to the requirement of spliced and unspliced counts. [a]Although Seurat does not provide a continuous cell cycle pseudotime or ordering, it does provide gene signature scores and assign discrete cell cycle phases. scPrisma does not perform well in cases where only a subset of cells are cycling (Figure **??**F).

	CellUntangler			Revelio	scPrisma	SiFT	Seurat	Baseline 1	Baseline 2
	$\mathbf{z}^1 \in \mathbb{H}^2$	$\mathbf{z}^1 \in \mathbb{E}^2$	$\mathbf{z}^1 \in \mathbb{E}^{10}$						
iLISI	0.592	0.611	0.618	**0.654**	0.551	0.646	0.649	0.442	0.457
ARI Leiden (15)	0.472	0.446	0.399	**0.515**	0.280	0.167	0.347	0.148	0.152
NMI Leiden (15)	**0.406**	0.387	0.346	0.400	0.262	0.231	0.334	0.196	0.206
ARI Louvain (15)	**0.468**	0.435	0.348	0.299	0.228	0.142	0.308	0.166	0.167
NMI Louvain (15)	**0.395**	0.364	0.303	0.310	0.217	0.181	0.339	0.162	0.237
ARI Leiden (50)	**0.562**	0.548	0.495	0.313	0.272	0.193	0.281	0.117	0.166
NMI Leiden (50)	**0.467**	0.462	0.412	0.275	0.267	0.227	0.320	0.147	0.223
ARI Louvain (50)	0.489	**0.529**	0.505	0.231	0.271	0.171	0.259	0.080	0.147
NMI Louvain (50)	0.420	**0.442**	0.424	0.275	0.267	0.206	0.319	0.060	0.159
cLISI	**0.834**	0.813	0.812	0.404	0.321	0.270	0.355	0.370	0.452

Table S3: Benchmarking of cell cycle signal removal and revealing of cell identity as a knockout (Ago2KO) cell or wild type (WT) cell on the HeLa data, related to Figure 2. iLISI is reported with respect to cell cycle phase where values closer to 1 indicate better cell cycle removal. Both Leiden and Louvain clustering were used. The number in brackets indicates the number of neighbors used. For the rest of the metrics, the default values were used. The average over ten runs for each of these metrics is reported for CellUntangler. Baseline 1 removes the cell cycle genes in our list and Baseline 2 removes the Revelio genes. iLISI: integration local inverse Simpson's index; ARI: adjusted Rand index; NMI: normalized mutual information; cLISI: normalized cell-type local inverse Simpson's index. The higher the better for these metrics.

	CellUntangler			tricycle	scPrisma	Revelio
	$\mathbf{z}^1 \in \mathbb{H}^2$	$\mathbf{z}^1 \in \mathbb{E}^2$	$\mathbf{z}^1 \in \mathbb{E}^{10}$			
Cell cycle correlation	0.805	**0.833**	0.807	0.516	0.590	0.584

Table S4: Comparison of cell cycle reconstruction for the mESCs data, related to Figure S2. The transcriptional phase output by DeepCycle[1], as analyzed by the original authors of this dataset, is used as the ground truth. The average correlation over ten runs is reported for CellUntangler.

References

1. Riba, A., Oravecz, A., Durik, M., Jiménez, S., Alunni, V., Cerciat, M., Jung, M., Keime, C., Keyes, W. M., and Molina, N. (2022). Cell cycle gene regulation dynamics revealed by RNA velocity and deep-learning. Nature Communications *13*, 2865. 10.1038/s41467-022-30545-8.
