# Supplementary material for: CellUntangler: Separating distinct biological signals in single-cell data with deep generative models
Source: Cell Genom. 2025 Dec 1;6(2):101073. doi: 10.1016/j.xgen.2025.101073 (PMC12903416; doi:10.1016/j.xgen.2025.101073)
Supplement: Document S3. Article plus supplemental information [file mmc4.pdf]

Cell Genomics

CellUntangler: Separating distinct biological signals in single-cell data with deep generative models

Graphical abstract

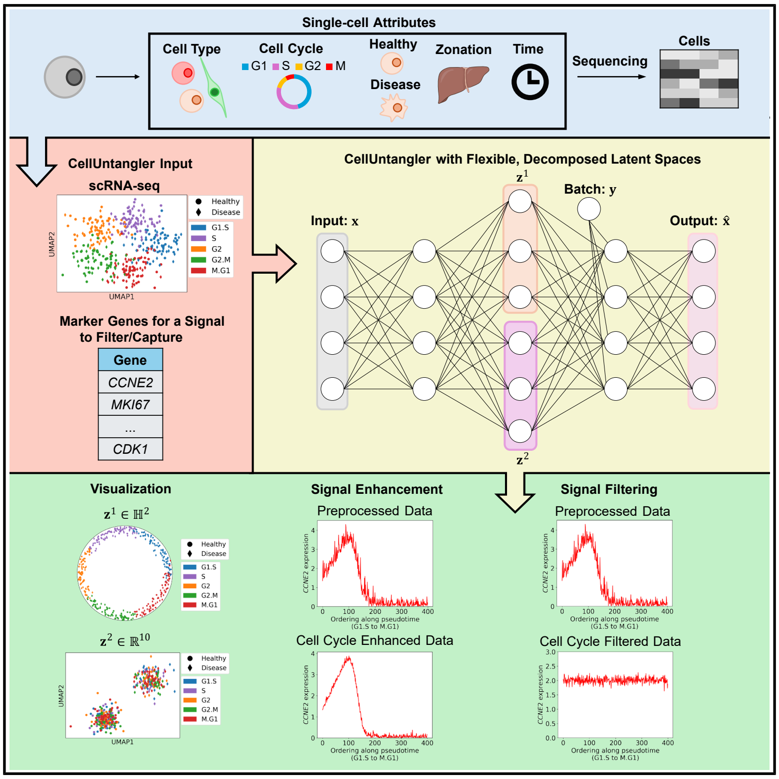

Authors

Sarah Chen, Aviv Regev, Anne Condon, Jiarui Ding

Correspondence

jiarui.ding@ubc.ca

In brief

Chen et al. present CellUntangler, a deep-learning model that utilizes a flexible latent space composed of multiple subspaces, allowing for the capture and filtering of biological signals in single-cell RNA sequencing data. CellUntangler demonstrates broad extensibility across a wide range of signals.

Highlights

- CellUntangler disentangles multiple biological signals in scRNA-seq data
- Applicable to diverse signals such as the cell cycle and spatial zonation
- Captures cell-cycle dynamics in datasets with cycling-only and mixed cells
- Identifies marker genes associated with signals of interest

Chen et al., 2026, Cell Genomics 6, 101073
February 11, 2026 © 2025 The Author(s). Published by Elsevier Inc.
https://doi.org/10.1016/j.xgen.2025.101073

CellPress

Cell Genomics

CellPress
OPEN ACCESS

Technology

CellUntangler: Separating distinct biological signals in single-cell data with deep generative models

Sarah Chen,[1] Aviv Regev,[2,3] Anne Condon,[1] and Jiarui Ding[1,4,*]
[1]Department of Computer Science, University of British Columbia, Vancouver, BC V6T 1Z4, Canada
[2]Broad Institute of MIT and Harvard, Cambridge, MA, USA
[3]Present address: Genentech, South San Francisco, CA, USA
[4]Lead contact
*Correspondence: jiarui.ding@ubc.ca
https://doi.org/10.1016/j.xgen.2025.101073

SUMMARY

Single-cell RNA sequencing has provided new insights into both intracellular and intercellular processes. However, multiple processes, such as cell-type programs, differentiation, and the cell cycle, often occur simultaneously within one cell. Existing methods typically target a single process and impose restrictive assumptions, risking the loss of valuable biological information. We introduce CellUntangler, a deep generative model that embeds cells into a latent space composed of multiple subspaces, each tailored with an appropriate geometry to capture a distinct signal. Applied to datasets of cycling-only and mixed cycling/non-cycling cells, CellUntangler disentangles the cell cycle from other processes such as cell type. The framework generalizes to disentangle additional signals, including spatial, tissue dissociation, interferon response, and cell-type identity. By providing flexible embeddings to capture various signals, CellUntangler enables selective enhancement or filtering of signals at the gene-expression level, offering a powerful tool for disentangling complex biological processes in single-cell data.

INTRODUCTION

Single-cell RNA sequencing (scRNA-seq) measurements of gene-expression levels in individual cells have facilitated the identification of new cell types, the discovery of gene regulatory networks, and an enhanced understanding of spatial and temporal processes within and across cells.[1] Multiple processes are simultaneously active within each cell, including the cell cycle, cell differentiation, the cell's type, and environmental responses. Each of these processes relies on different gene activities, and the cells may relate to each other differently depending on the context (e.g., as separable clusters for cell types, continuous trajectories and bifurcations for differentiation, or a circle for the cell cycle).

This poses two analysis challenges. First, one or more prominent processes (e.g., cell cycle or cell type) may confound or obscure others during analysis, whereas an ideal analysis would uncover each of the processes and the cell relations in its own context. Second, identifying and filtering out stronger signals (e.g., the cell cycle) to reveal any other residual ones is often a bespoke procedure that does not generalize well. Poor filtering of the effects from confounding factors can negatively impact downstream analysis of scRNA-seq data, but removal of a signal can also lead to the loss of biological information. For example, consider the cell cycle. Removing cell-cycle effects can uncover hidden signals, enabling the identification of distinct cell subpopulations.[2] Without proper removal, variability in gene expression among cells of the same type may be attributed to the cell cycle, causing cells to cluster by their cell-cycle stage rather than their true biological identity.[3] In addition to being a confounder of biological signals, the cell cycle is itself a critical biological process and can differ between cell populations at different differentiation states.[4] Multiple methods have been developed for cell-cycle inference and filtering. Some methods focus solely on removing the effect (e.g., ccRemover[3]) as a preprocessing step for downstream analyses, whereas others (e.g., tricycle,[5] DeepCycle,[6] reCAT,[7] and SC1CC[8]) infer cell-cycle pseudotime but do not remove its effects. Methods that perform both inference and filtering often rely on assumptions that may limit their applicability. For instance, Cyclum[9] embeds all cells onto a one-dimensional circle, which can incorrectly position non-cycling cells along the circle manifold. Moreover, while many existing methods are tailored exclusively to the cell cycle (or cyclic processes), other biological processes of interest remain largely unaddressed. For example, Cyclum is applicable only to cyclic processes; scPrisma[10] can work for different signals and utilizes different topologies, but struggles with datasets that include non-cycling cells; and SiFT[11] requires multiple steps, including calculating the similarity between cells, and does not produce an embedding of cells for each signal of interest. All methods described usually require separate enhancing and filtering steps, followed by dimensionality reduction. Batch-effect removal would be another step as well.

© 2025 The Author(s). Published by Elsevier Inc.
This is an open access article under the CC BY license (http://creativecommons.org/licenses/by/4.0/).

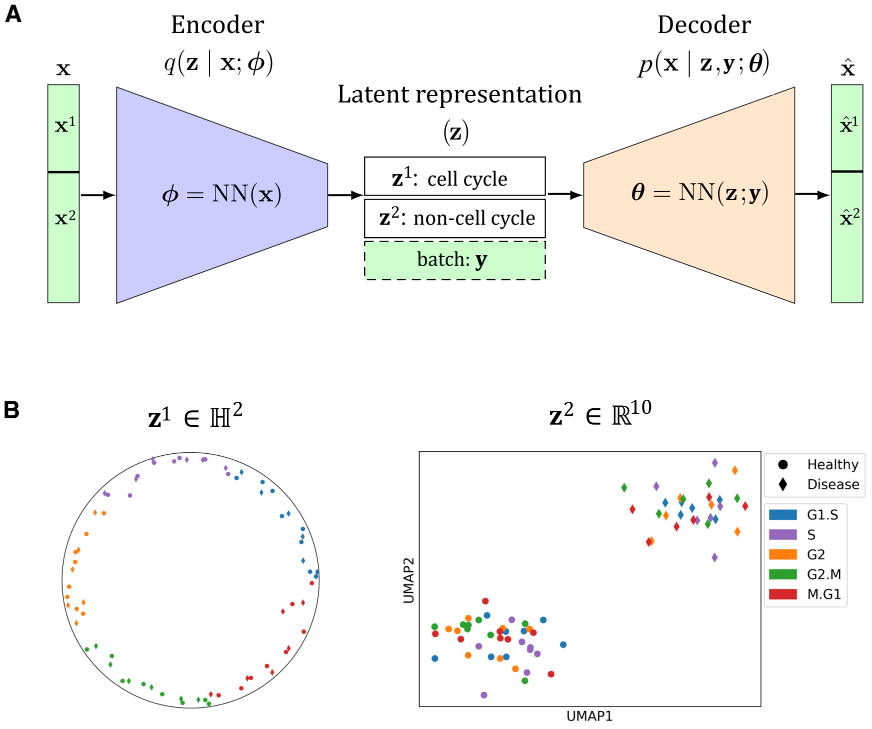

Figure 1. Method overview

(A) CellUntangler takes as input scRNA-seq measurements and one or more batch effects and embeds cells into decomposed latent subspaces with geometries tailored to separate distinct biological signals into individual representations. The CellUntangler model with two latent subspaces: one to capture the cell cycle, $\mathbf{z}^1$, and the other to capture non-cell-cycle-specific signals, $\mathbf{z}^2$. The parameters of $q(\mathbf{z}|\mathbf{x};\boldsymbol{\phi})$ and $p(\mathbf{x}|\mathbf{z},\mathbf{y};\boldsymbol{\theta})$ are represented by $\boldsymbol{\phi}$ and $\boldsymbol{\theta}$, respectively.

(B) A manually created illustration of how CellUntangler might disentangle two biological signals. The cell cycle (cell-cycle stage indicated by color) obscures the control and disease signal (indicated by shape). CellUntangler disentangles these signals by embedding the cell-cycle signal in $\mathbf{z}^1$, revealing the control and disease signal in $\mathbf{z}^2$. $\mathbf{z}^2$ is projected to 2D using UMAP for visualization.

Here, we develop CellUntangler, a deep-learning model trained end-to-end to output separate latent representations for different biological processes while simultaneously addressing batch effects, eliminating the need for multiple, separate steps. CellUntangler leverages variational autoencoders (VAEs),[12–14] which have been shown to perform effectively in scRNA-seq analysis.[15–19] We demonstrate CellUntangler's effectiveness by applying it in several contexts. With a cycling cell line dataset and a mouse embryonic stem cell dataset, CellUntangler produced a latent representation that accurately captured the cell-cycle signal and removed its confounding effects in a second latent representation. With a dataset of cycling and non-cycling immune cells, non-cycling cells clustered together in the latent subspace designed for the cell-cycle signal, and the cell type identities of cycling cells were discernible in the latent subspace encoding cell-cycle-independent information. With mouse pancreatic cells, CellUntangler separately captured cell-cycle and differentiation trajectory signals in two non-Euclidean subspaces. In mouse liver hepatocytes, it successfully captured and separated the spatial zonation signal from temporal signals. Finally, CellUntangler disentangled the interferon (IFN)-response signal, tissue-dissociation signal, and cell-type identity from other signals, as demonstrated in analyses of approximately 1 million cells from high-grade serous ovarian cancer patients, mast cells from healthy and eosinophilic esophagitis patients, and human peripheral blood mononuclear cells treated with interferon beta (IFN-β).

DESIGN

CellUntangler (Figure 1A) is a deep-learning model that leverages VAEs to embed high-dimensional scRNA-seq profiles into low-dimensional latent spaces. Unlike previous approaches, which typically use one latent space, resulting in a single representation per cell, CellUntangler's latent space is composed of multiple subspaces, each capturing a distinct signal, allowing CellUntangler to simultaneously capture and filter multiple biological signals. Such decomposed subspaces have been used to encode drug treatments and have proved effective in predicting gene-expression profiles under unseen drug combinations.[20]

CellUntangler takes as input an scRNA-seq dataset $\mathcal{D} = (\mathbf{x}_i, \mathbf{y}_i)_{i=1}^{N}$ with N cells, where $\mathbf{x}_i$ is the unique molecular identifier (UMI) count vector of G genes in cell i, and $\mathbf{y}_i$ is a categorical vector indicating the batch or batches in which $\mathbf{x}_i$ is measured. We model $\mathbf{x}_i$ as being governed by a latent low-dimensional vector $\mathbf{z}_i$ composed of k distinct components, $\mathbf{z}_i = \left(\mathbf{z}_i^1, \mathbf{z}_i^2, \ldots, \mathbf{z}_i^k\right) \in \mathbb{R}^d$. The final component, $\mathbf{z}_i^k$, is used to capture additional signals not accounted for by the first k-1 latent components. For example, two components may be used: $\mathbf{z}_i^1$ to capture the desired signal (e.g., cell cycle) and $\mathbf{z}_i^2$ to capture signal-independent processes. Importantly, the geometry of each component can be Euclidean or non-Euclidean[21,22] and users can select the geometry that best aligns with the nature of the signal of interest. It has previously been shown that non-Euclidean latent spaces can be beneficial for scRNA-seq data analysis.[23,24]

To disentangle the cell cycle or other desired signals into individual components $\mathbf{z}_i^j$, CellUntangler also requires a set of marker genes for the target signal. Based on the marker genes for each component, CellUntangler decomposes $\mathbf{x}_i$ into k UMI count vectors, $\mathbf{x}_i = \left(\mathbf{x}_i^1, \mathbf{x}_i^2, \ldots, \mathbf{x}_i^k\right) \in \mathbb{R}^G$, and uses a neural network decoder to model each UMI count vector $\mathbf{x}_i^j$ given $\mathbf{z}_i$. When reconstructing $\mathbf{x}_i^j$ using $\mathbf{z}_i$, the gradient for $\mathbf{z}_i^l$ where $l \neq j$ may be stopped (Figure S1). By default, the gradient is not stopped for $\mathbf{z}^j$. The batch vector $\mathbf{y}_i$ is also passed to the decoder to correct for batch effects. The CellUntangler decoder allows us to obtain data enhanced or filtered for a specific signal at the gene-expression level, which is useful for downstream analyses.

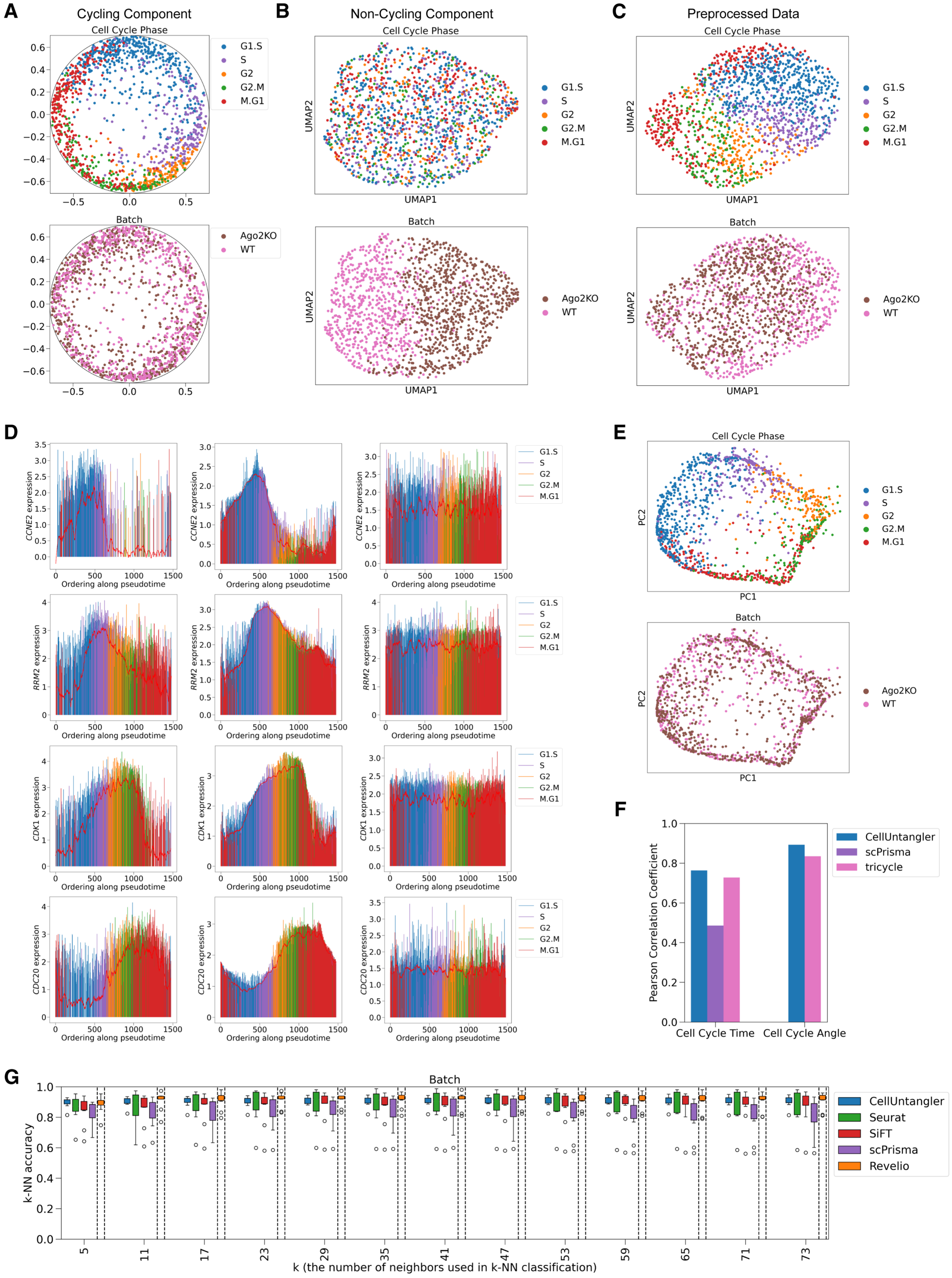

Figure 2. CellUntangler allows us to separate the cell-cycle signal from the WT and knockout, Ago2KO, signal present in the HeLa dataset

(A) The first component of CellUntangler, $\mathbf{z}^1$, captures the cell cycle.

(B) The second component, $\mathbf{z}^2$, projected to 2D using UMAP, filters out the cell cycle and separates WT cells and knockout cells.

(C) When using the standard preprocessing pipeline, cells separate by cell-cycle stage. WT and knockout cells are mixed together.

(legend continued on next page)

To estimate the posterior distributions for each component $\mathbf{z}_i^j$, which are intractable to compute directly, we turn to variational inference and use a neural network encoder to output the parameters of the posterior distributions. We provide full details in the STAR Methods section.

RESULTS

CellUntangler captures and removes the cell cycle to reveal hidden biological signals

We initially focused on separating the cell cycle from other processes, as cycling cell profiles are expected to exhibit a circular structure (when considering cell-cycle genes) due to the cyclical increase and decrease of gene expression during cell-cycle progression.[25–27] We modeled the latent distribution of the cell cycle in a hyperbolic space of dimension two, using the rotated hyperbolic wrapped normal distribution (RoWN)[28] in the Lorentz model of the hyperbolic space. A hyperbolic space of dimension two provides a convenient embedding into the Poincaré disk when the Lorentz coordinates are projected to Poincaré coordinates, and the RoWN models local variation in the radial direction. We relied on a curated set of cell-cycle marker genes (Table S1). For cell-cycle-independent processes, either the Euclidean or the hyperbolic space may be more suitable, depending on the nature of the signal. Importantly, using separate latent subspaces allows us to capture and remove the cell-cycle signal from other signals (Figure 1B).

We initially applied CellUntangler to scRNA-seq of wild-type (WT) and Ago2 knockout (KO) cells,[29] showing that it effectively captured signals in two spaces: one ($\mathbf{z}^1$) reflecting the cell cycle (and mixing the genotypes) (Figure 2A) and the other ($\mathbf{z}^2$) reflecting the genotype (and removing the cell cycle) (Figure 2B). The cell-cycle representation was in the hyperbolic space with the RoWN, $\mathbf{z}^1 \in \mathbb{H}^2$, and the cell-cycle-independent one was in Euclidean space, $\mathbf{z}^2 \in \mathbb{R}^{10}$. In $\mathbf{z}^1$ (Figure 2A), cells also followed the correct progression of the cell-cycle stages: G1.S → S → G2 → G2.M → M.G1 and back to G1.S. Conversely, a standard scRNA-seq processing and visualization pipeline[30–35] entangled the two signals (Figure 2C), with the cell cycle dominating over the genotype.

We validated the cell-cycle signal by examining the expression of individual genes in cells ordered along a pseudotime, obtained by projecting $\mathbf{z}^1$ from Lorentz to Poincaré coordinates, then measuring the angle of the projected Poincaré coordinates for each cell relative to the origin of the Poincaré disk, (0, 0) (Figure 2A; STAR Methods) (Figure 2D, left; Figure S2A, left). Cell-cycle-related genes exhibited expected expression peaks. For example, *CCNE2* peaked shortly after the G1.S transition; *RRM2* peaked during S phase, followed by *CDK1* peaking at the G2.M transition; and *CDC20* peaked at M phase (Figure 2D, left).

By passing $\mathbf{z}^1$ to CellUntangler's decoder, we enhanced the cell-cycle signal present in the data at the gene-expression level, yielding smoother gene-expression trends with reduced noise (Figure 2D, middle). This enhancement was consistent across other putative cell-cycle marker genes used by Schwabe et al.[29] to capture the cell-cycle signal present in the same dataset (Figure S2A, middle), even though these genes were not included as marker genes for our model.

Similarly, passing $\mathbf{z}^2$ to CellUntangler's decoder filtered out the cell-cycle signal at the gene-expression level. Expression of cell-cycle-related genes was flattened (Figure 2D, right; Figure S2A, right). Performing principal-component analysis (PCA) on the enhanced and filtered data further confirmed the effectiveness of CellUntangler: the cell-cycle signal was either enhanced or filtered out as intended (Figures 2E and S2B), and vice versa for the genotype.

We compared CellUntangler to other recent or widely used methods for cell-cycle reconstruction and removal (Table S2). The quality of our cell-cycle reconstruction compared favorably to other methods (Figure 2F; STAR Methods), even on this small dataset of only 1,477 cells. To assess the effectiveness of cell-cycle removal, we used *k*-nearest neighbor (*k*-NN) classification accuracy (Figure 2G) to distinguish between knockout and WT cells, using 10-fold cross-validation. CellUntangler was among the top-performing methods for smaller values of *k*. Although Revelio performed better, it relies on a larger number of marker genes and requires prior knowledge of the association between genes and specific cell-cycle phases (STAR Methods). When using the same cell-cycle genes as Revelio, CellUntangler tended to perform similarly to or better than Revelio (Figure S2C).

We further compared the performance of CellUntangler when using the Euclidean space for the cell-cycle component instead of the hyperbolic space with the RoWN. We used the Euclidean space with a dimension of two, $\mathbf{z}^1 \in \mathbb{E}^2$, and the Euclidean space with a dimension of 10, $\mathbf{z}^1 \in \mathbb{E}^{10}$. When using the hyperbolic space, cells were placed near the edge of the Poincaré disk. However, for the Euclidean space, due to the Kullback-Leibler (KL)-divergence term, cells were placed closer to the origin, making the gap in the circle of cycling cells harder to observe (Figures S2D and S2E). The cell-cycle correlations were highest when using the hyperbolic space with the RoWN (Figure S2F). Regarding the classification accuracy of WT and knockout cells, CellUntangler with the hyperbolic space for the cell-cycle component outperformed using a Euclidean space of dimension 10 and had a similar performance to using a Euclidean space of dimension two (Figure S2G).

(D) Gene expression of *CCNE2* (G1.S), *RRM2* (S), *CDK1* (G2), and *CDC20* (M) as a function of pseudotime obtained with the embeddings from CellUntangler using the original preprocessed data (size-factor normalized and log1p transformed, left) and the reconstructed gene-expression output from CellUntangler after decoding $\mathbf{z}^1$ (middle) and $\mathbf{z}^2$ (right). Cell-cycle stages (G1.S, S, G2, M) are in parentheses.
(E) PCA representation of the reconstructed data obtained by running $\mathbf{z}^1$ through CellUntangler's decoder.
(F) Comparison of cell-cycle reconstruction.
(G) Accuracy of *k*-nearest neighbors on WT and knockout cells using $\mathbf{z}^2$ from CellUntangler and different methods. Boxplots depict the medians and the interquartile ranges (IQRs). The whiskers show the lowest datum still within 1.5 IQR of the lower quartile and the highest datum still within 1.5 IQR of the upper quartile. Individual points below and above the whiskers indicate outliers.

We evaluated additional metrics[36] for cell-cycle effect removal and the ability to reveal cell identity (knockout or WT) (Table S3) and found that CellUntangler performed well across these metrics, achieving the best scores based on adjusted Rand index, normalized mutual information, and cell-type integration local inverse Simpson's index.

CellUntangler captures and removes the cell cycle in mouse embryonic stem cells

We applied CellUntangler to a mouse embryonic stem cells dataset (using all available genes), originally presented by Riba et al.[6] to study the cell cycle in this context. We used the hyperbolic space with the RoWN, $\mathbf{z}^1 \in \mathbb{H}^2$, to capture the cell cycle and the Euclidean space, $\mathbf{z}^2 \in \mathbb{E}^{10}$, to capture non-cell-cycle-specific signals. The results from CellUntangler agreed with the expression phase from the original study, as the $\mathbf{z}^1$ vectors corresponding to the cells formed a circle, where the initial expression phase, $\theta = 0.0$, progressed to the final expression phase, $\theta = 1.0$ (Figure S3A). Additionally, the cell-cycle effects were nondiscernible in $\mathbf{z}^2$ (Figure S3B).

We validated the results by examining the expression of cell-cycle genes in cells ordered by pseudotime (Figure S3C, left), calculated by measuring the angular position of cells relative to the origin in $\mathbf{z}^1$ (Figure S3A; G1 at $\theta = 0.0$ to M at $\theta = 1.0$, STAR Methods). Expression patterns follow expected trends for *Ccne2* (peaks at G1.S transition), *Rrm2* (peaks at S phase), *Cdk1* (peaks at G2.M transition), and *Cdc20* (peaks at M phase). CellUntangler's data reconstructions based on $\mathbf{z}^1$ showed reduced expression noise in cell-cycle genes, while the reconstruction from $\mathbf{z}^2$ exhibited flattened expression in these genes (Figure S3C, middle and right). The cell-cycle signal was enhanced in the PCA representation of the data reconstruction obtained with $\mathbf{z}^1$ and effectively filtered on the data reconstruction with $\mathbf{z}^2$ (Figures S3D and S3E).

We additionally used the Euclidean space with a dimension of two (Figure S3F) and the Euclidean space with a dimension of 10 (Figure S3G) to capture the cell-cycle signal. Again, cells were placed close to the origin in the Euclidean spaces. The cell-cycle pseudotime obtained with CellUntangler had a higher correlation with the expression-phase output from the original study than other methods (Table S4).

CellUntangler provides insight into the cell-cycle populations containing both cycling and non-cycling cells

Single-cell RNA sequencing datasets typically contain both cycling and non-cycling cells. Although the cycling cells may belong to different types or subsets, they will often all group together in analysis due to the prominent signal from one program (cell cycle) dominating others (cell-type programs), making it difficult to determine their cell-type identities. Conversely, non-cycling cells can make capturing the cell cycle more challenging, as methods may try to assign a phase to each cell assuming it is cycling, or rely on a topological prior, causing them to fail as they attempt to place all cells on a circular manifold. Datasets may also contain technical and biological batch effects, further complicating the analysis.

To assess the ability of CellUntangler to tackle such challenges, we first analyzed a myeloid cell atlas collected across multiple tissues, donors (of both biological sexes), ages, and lab techniques and previously annotated as classical and nonclassical monocytes, conventional type-one dendritic cells (DC1s), conventional type-two dendritic cells (DC2s), migratory dendritic cells (migDCs), mononuclear phagocyte/T cell (MNP/T) doublets, and cycling cells (without a specific cell-type assignment).[37] As above, we used a hyperbolic space with the RoWN; $\mathbf{z}^1 \in \mathbb{H}^2$ to capture the cell cycle; and a Euclidean space, $\mathbf{z}^2 \in \mathbb{R}^{10}$, to capture the non-cell-cycle-specific information.

In the cycling component ($\mathbf{z}^1$), the cycling cells were initially placed along the edge of the Poincaré disk, while the other cells were distributed throughout the interior of the disk (Figure 3A). Following recentering the origin of the disk, we obtained the expected circular arrangement of cycling cells ordered along the disk circumference in the expected sequential order (Figure 3B, top), surrounding non-cycling cells (Figure 3B; STAR Methods). In both the original and recentered disks, non-cycling cells were clearly mixed with respect to cell type (Figure 3A, bottom; Figure 3B, bottom), confirming that $\mathbf{z}^1$ selectively captured cell-cycle information.

Conversely, in the second component ($\mathbf{z}^2$), all cells, including cycling cells, were separated by cell type (Figure 3C). Notably, cycling cells of all phases were integrated with other cell types (Figures S4A and S4B), especially monocytes and DC2s. The small patch of cycling cells likely represents macrophages (discussed below). To validate that the second component, $\mathbf{z}^2$, successfully captured cell-type information, we performed k-NN classification on the manually curated cell types (Figure 3D), achieving a high accuracy of 0.94 for different ks, even though, as expected, these myeloid cells do not form very discrete clusters. (Cycling cells were excluded from the k-NN experiments.)

Moreover, the second component mitigated the technical batch effects well, such as different sequencing techniques, while preserving biological variation, such as organ (Figure 3E). When we used the Euclidean space with a dimension of two to capture the cell-cycle signal, $\mathbf{z}^1 \in \mathbb{E}^2$, cycling and non-cycling cells were only separated from each other (Figure S4C), but the cycling cells did not form a circle. Results were similar when using the Euclidean space with a dimension of 10 to capture the cell-cycle signal (Figure S4D). Other methods struggled with this use case as well. For example, scPrisma incorrectly placed non-cycling cells on the circular manifold when attempting to enhance the cell-cycle signal (Figure 3F); the standard Scanpy pipeline, even after regressing out the cell-cycle signal, placed cycling cells into distinct clusters (albeit in proximity to specific cell types) (Figure S4E), and strong batch effects remained (Figure S4F).

We used the k-NN classifier to determine the identity of the cycling cells (STAR Methods) and confirmed those predictions by examining known marker-gene expression (Figures 3G and S4G). Cycling cells predicted to be classical monocytes expressed *CD14*, predicted DC1s expressed *XCR1* and *CLEC9A*, and predicted DC2s expressed *CD1C* and *CLEC10A*. We hypothesized that many cycling cells predicted as MNP/T doublets or uncertain (when classifiers with different values of k did not predict the same cell type) were macrophages, because the original dataset contained macrophages, which we filtered out prior to running CellUntangler. The macrophages in the

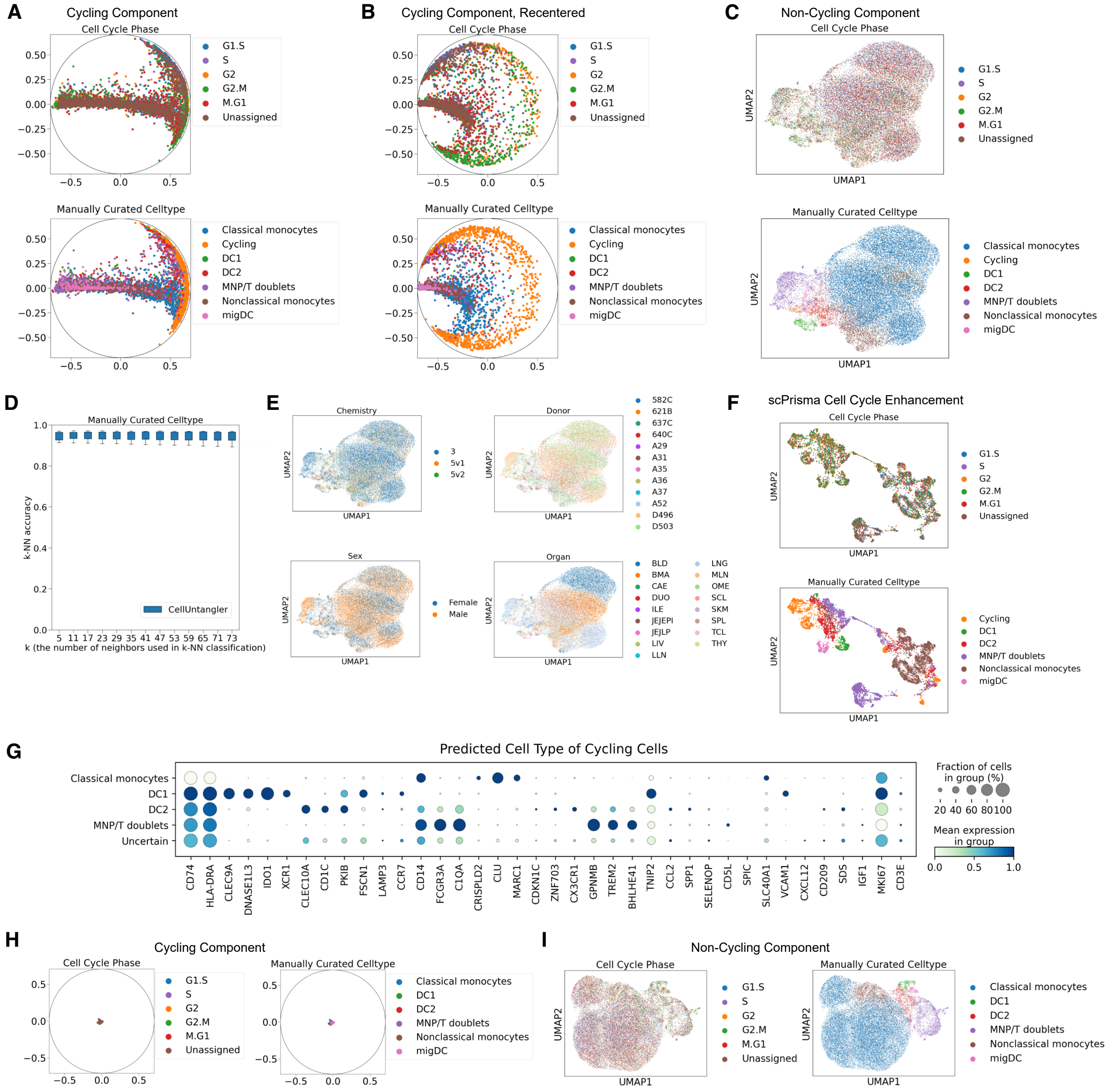

Figure 3. CellUntangler outputs embeddings that separately capture the cell cycle and cell type

Embeddings capturing cell-type information no longer contain the effects of the cell cycle.

(A and B) (A) The embeddings of the first component, $\mathbf{z}^1$, before recentering, and (B) after recentering the origin to (−0.55, 0.0).

(C) The embeddings of the second component, $\mathbf{z}^2$, after projecting to 2D using UMAP.

(D) The *k*-NN accuracies on the manually curated cell type using $\mathbf{z}^2$, excluding cycling cells. Boxplots depict the medians and the interquartile ranges (IQRs). The whiskers are the lowest datum still within 1.5 IQR of the lower quartile and the highest datum still within 1.5 IQR of the upper quartile.

(E) The embeddings of the second component, $\mathbf{z}^2$, colored by the batches present in the dataset.

(F) UMAP of the cells after enhancing the data for the cell-cycle signal using a cyclic topological prior with scPrisma.

(G) Marker gene expression for predicted cell type of the cycling cells.

(H and I) (H) The embeddings of the first component, $\mathbf{z}^1$, and (I) the second component, $\mathbf{z}^2$, when the dataset is composed of only non-cycling cells.

original dataset included alveolar and intermediate macrophages (from the lung), intestinal macrophages (jejunum), and erythrophagocytic macrophages (spleen, liver, mesenteric lymph nodes) (Figure S4H).[37] These cells did not express the expected T cell marker genes for MNP/T doublets. Most of these predicted MNP/T doublets were found in the lung and

expressed markers of alveolar macrophages, including *GPNMB* and *TREM2* (Figure S4I). These cycling cells may have been classified as MNP/T doublets because some of the MNP/T doublets may contain macrophages, and the MNP/T doublets also expressed *GPNMB* and *TREM2* (Figure S4A). When examining the distribution of uncertain cells by organ (Figure S4J), we observed that cycling cells present in the jejunum expressed intestinal macrophage markers *CD209* and *IGF1*. Similarly, cycling cells present in the lung expressed *GPNMB* and *TREM2*, markers of alveolar macrophages.

Datasets may consist entirely of non-cycling cells. To evaluate this scenario, we ran CellUntangler on the myeloid cell dataset after excluding all cells annotated as cycling, using the same model settings. In $\mathbf{z}^1$, the cells no longer formed a circle, as the non-cycling cells were placed in the origin (Figure 3H), whereas cell-type information was still well captured in $\mathbf{z}^2$ (Figure 3I).

We also ran CellUntangler on a retina cell dataset consisting of 121,309 cells.[38] The dataset contains various cell types, primarily immune cells and macroglia, including both cycling and non-cycling microglia, as well as a very rare cycling astrocyte subset that was only identified through sub-clustering of the astrocytes in the original study.[38] We used CellUntangler to capture the cell cycle in the hyperbolic space with the rotated wrapped normal distribution, $\mathbf{z}^1 \in \mathbb{H}^2$, and the Euclidean space to capture non-cell-cycle-specific signals, $\mathbf{z}^2 \in \mathbb{E}^{10}$. In the cell-cycle component, cycling microglia were placed along the edge of the Poincaré disk (Figure S5A). Importantly, the rare cycling astrocytes also lay along the edge of the Poincaré disk. After recentering the origin, the cycling microglia formed a circle surrounding the non-cycling cells (Figure S5B). In the non-cell-cycle component, cells separated by cell type (Figure S5C). As expected, both cycling microglia and cycling astrocytes expressed canonical cell-cycle genes (Figures S5D and S5E). After removing the cell-cycle effects, the cycling microglia were integrated with the non-cycling microglia (Figures S5F and S5G).

CellUntangler captures differentiation trajectories

Thus far, we have used the Euclidean space to capture the non-cycling component. However, some programs, such as those that govern cell differentiation trajectories, may be better represented in the hyperbolic space.[23,24] To demonstrate the versatility of CellUntangler, we tested it on a dataset of mouse pancreatic cells,[39] which included both cycling and non-cycling cells, undergoing differentiation. This dataset exhibited two distinct signals: a cell-cycle signal from the cycling cells and a strong differentiation trajectory signal for endocrine cells (alpha, beta, delta, and epsilon cells).

To capture these signals, we employed CellUntangler with two hyperbolic spaces: one for the cell cycle ($\mathbf{z} \in \mathbb{H}^2$, with the RoWN) and another for differentiation trajectories ($\mathbf{z} \in \mathbb{H}^2$). As expected, the first component ($\mathbf{z}^1$) successfully captured the cell-cycle signal (Figure 4A). After recentering, the cycling cells were arranged in a circular pattern around the non-cycling cells (Figure 4B; STAR Methods). Importantly, the second component ($\mathbf{z}^2$) effectively captured the differentiation trajectory. In the pancreas, ductal cells gradually induce *Ngn3* first to become *Ngn3*-low cells, followed by *Ngn3*-high cells, and, finally, the *Ngn3*-high cells express the transcription factor *Fev* (pre-endocrine cells) and differentiate into alpha, beta, delta, or epsilon cells (Figure 4C).

Further analysis of the pre-endocrine cells[39] determined which cell type the cells were committed to. As expected, the *Fev*$^+$ alpha cells were placed before the alpha cells and, similarly, for the beta and delta cells. However, in the uniform manifold approximation and projection (UMAP) embeddings from studying pancreas cell development,[40] the differentiation signal is entangled with the cell-cycle signal, and, notably, the fine-grained developmental trajectories are more clearly delineated in CellUntangler's $\mathbf{z}^2$ embeddings compared to the UMAP embeddings (Figure 4D). For example, the *Fev*$^+$ delta cells were placed before the mature delta cells in the hyperbolic embeddings but separated by the *Fev*$^+$ beta cells in the UMAP embeddings (Figures 4C and 4D). Importantly, the epsilon cells were split into two groups: *Acsl1*$^+$ epsilon cells[41] and *Acsl1*$^-$ epsilon cells (Figure 4E). The *Acsl1*$^+$ epsilon cells were placed following the *Fev*$^+$ epsilon cells and the *Acsl1*$^-$ epsilon cells were placed following the *Fev*$^+$ Pyy cells. Previous studies[39,40,42] also place the *Fev*$^+$ epsilon and *Fev*$^+$ Pyy cells before the epsilon cells, but the pattern is much clearer in CellUntangler's embeddings.

CellUntangler captures and filters spatial zonation signals

While the cell cycle is an illustrative and common form of a predominant signal, CellUntangler can generalize to other cases where we wish to capture, distinguish, or remove different processes in single cells.

To demonstrate this broader applicability, we applied CellUntangler to a dataset of hepatocytes profiled from the mouse liver.[43] Hepatocytes in the liver have both zonation programs, reflecting their spatial location (and distinct functional requirements) in the liver lobules,[44] and circadian programs, reflecting timing in the light-dark cycle. In this dataset, cells from across the lobules were sampled at four evenly spaced time points throughout the day. Using the standard preprocessing pipeline, the spatial and temporal signals are entangled (Figure 5A). CellUntangler can capture and filter the spatial signal so that it is separated from the temporal signal.

We used the Euclidean space to capture zonation in the first component, $\mathbf{z}^1 \in \mathbb{E}^2$, relying on 50 spatial marker genes[43] (Table S1) and the second component, $\mathbf{z}^2 \in \mathbb{E}^{10}$, for the non-zonal (circadian) signal. When viewing $\mathbf{z}^1$ individually by the different zonation layers, the organization followed the expected progression from layer 1 to layer 8 (Figure 5B) based on the second dimension coordinate (the *y* coordinates). Layer 1 cells were placed near the bottom of the plot, layer 2 cells were placed slightly above, and so on, all the way to layer 8 cells. By adding the median position of each layer in the *y* axis of $\mathbf{z}^1$, we could clearly see the monotonic increase in the *y* coordinates from layer 1 all the way to layer 8. Furthermore, the zonated genes *Cyp2f2* and *Cyp2e1* were expressed in opposite regions, since layers 1–8 were organized along the central-portal axis and *Cyp2f2* was portally expressed while *Cyp2e1* was centrally expressed (Figure 5C). The spatial and temporal signals were separated, since the cells from different times (zeitgeber time, 0, 6, 12, 18 h) were mixed in $\mathbf{z}^1$ (Figure 5D), whereas, in $\mathbf{z}^2$, the cells separated by time point only and not by layer (Figure 5E).

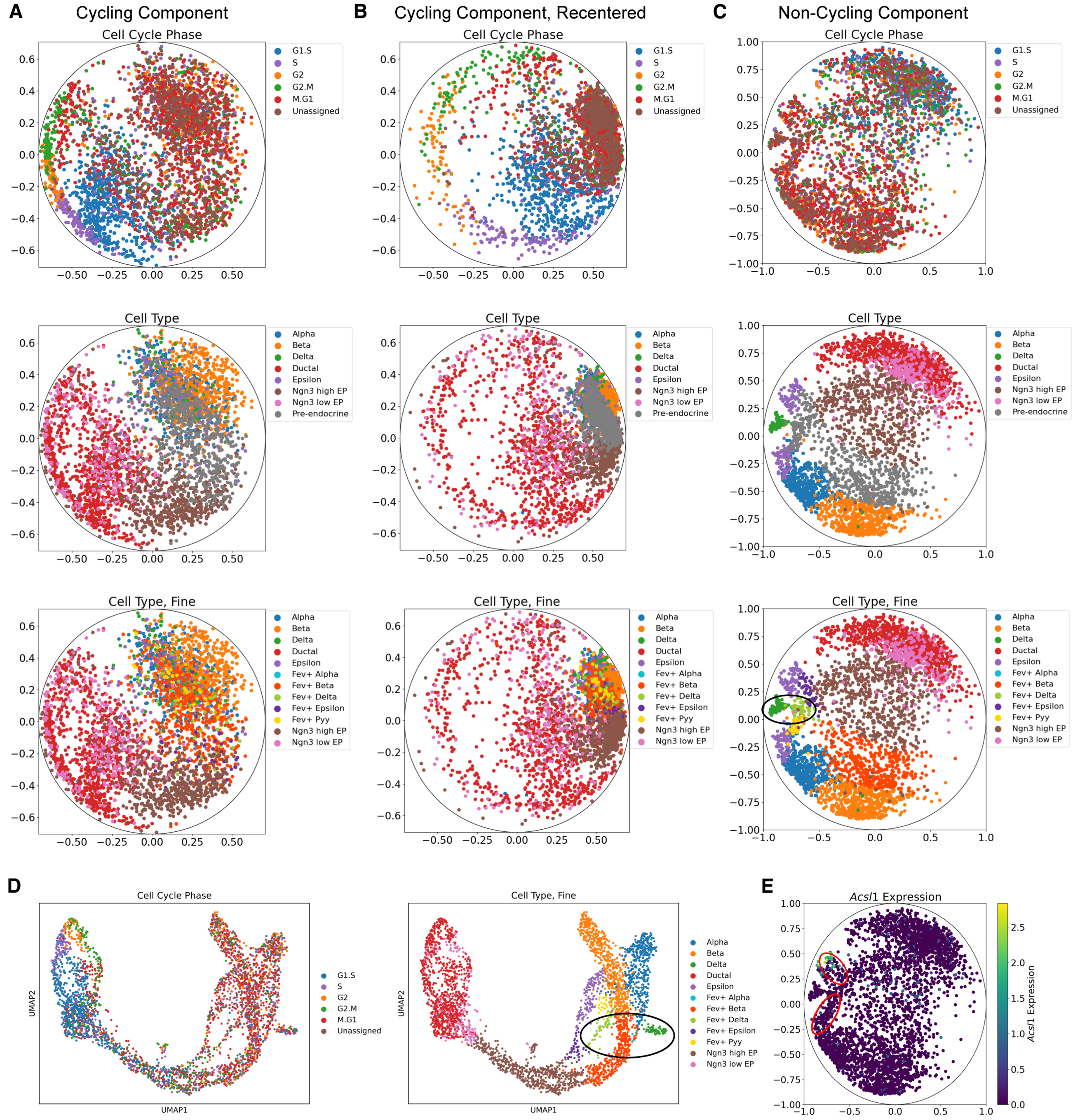

Figure 4. CellUntangler separates the cell cycle into one component and the differentiation trajectory into the other component

(A and B) (A) The embeddings of the first component, $\mathbf{z}^1$, before recentering and (B) after recentering the origin to be (0.4, 0.1).

(C) The differentiation trajectory is captured in the second component, $\mathbf{z}^2$.

(D) A previously published UMAP of the same cells input to CellUntangler from the mouse pancreas dataset.

(E) Cells are colored by *Acsl1* gene expression (size-factor normalized and log1p transformed). The *Acsl1*$^+$ epsilon cells and *Acsl1*$^-$ epsilon cells are circled in red (above and below).

In (C) and (D), the transition from Fev$^+$ delta to mature delta cells is circled in black.

We examined the expression of spatiotemporal informative genes (*Elovl3* and *Pck1*) in the preprocessed data, the data enhanced for zonation (passing $\mathbf{z}^1$ to the decoder), and the data with the zonation signal filtered (passing $\mathbf{z}^2$ to the decoder). For *Elovl3*, differential expression across zonation layers (Figure 5F, left) was retained for the zonation-enhanced data

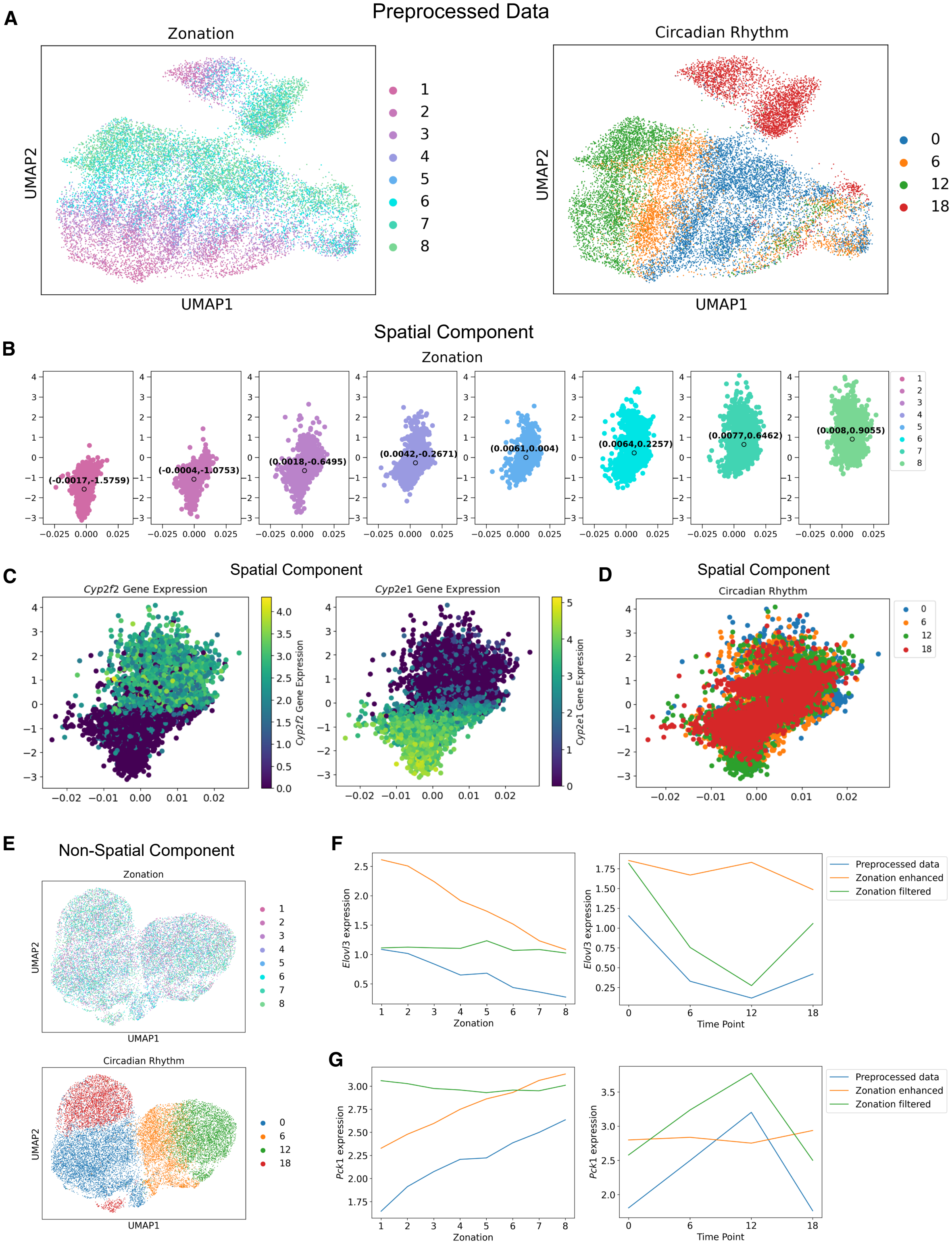

Figure 5. Spatial and temporal signals are captured and separated with CellUntangler

(A) When using the standard preprocessing pipeline, the spatial and temporal signals are entangled.

(B) The embeddings of the first component, $\mathbf{z}^1$, plotted by individual layer. The median coordinates of each layer are circled in black and labeled.

(legend continued on next page)

and flattened for the zonation-filtered data. Across circadian-rhythm time points (Figure 5G, right), *Elovl3* expression was flattened for the zonation-enhanced data and retained for the zonation-filtered data. Gene expression varied similarly for *Pck1* across zonation layers (Figure 5G, left) and circadian-rhythm time points (Figure 5G, right).

We next analyzed another scRNA-seq dataset of liver hepatocytes from mice in fed, fasted, and starvation states.[45] Again, we used the Euclidean space to capture zonation in the first component, $\mathbf{z}^1 \in \mathbb{E}^2$, relying on 50 spatial marker genes[43] (Table S1) and the second component, $\mathbf{z}^2 \in \mathbb{E}^{10}$, for the non-zonal (time after feeding) signal. The *y* axis of the 2D Euclidean latent space ($\mathbf{z}_2^1$, where the subscript 2 represents the second dimensional coordinate) effectively captured the spatial zonation signal (Figures 6A and 6B), with central hepatocytes (expressing marker gene *Cyp2e1*) located at the top and portal hepatocytes (expressing marker gene *Cyp2f2*) at the bottom. Latent dimensions 5 ($\mathbf{z}_5^2$) and 6 ($\mathbf{z}_6^2$) captured the time after feeding (Figure 6C), while the spatial zonation information was removed from $\mathbf{z}^2$, as shown by the lack of *Cyp2e1* expression variation in the $\mathbf{z}^2$ UMAP (Figure 6D). Importantly, by disentangling the spatial-zonation signal, we were able to identify a small IFN-response hepatocyte subset that had been missed in the original study (Figure 6E).

The second coordinate of the first component, $\mathbf{z}_2^1$, captured not only the portal-central axis but also more detailed spatial zonation information with the liver lobule. For example, we can see a gradient in *Slc1a2* expression at the very top, followed by the expression of *Cyp2a5*, and then that of the portal gene *Aldh1b1* at the bottom (Figure 6F). This pattern was consistent for the hepatocytes from different time points. Only at the starvation stage, nearly no hepatocytes expressed *Slc1a2*. The results align with previous findings that, under starvation, central hepatocytes take on gene-expression characteristics of portal hepatocytes.[45] Thus, $\mathbf{z}_2^1$ captured fine-grained spatial-zonation information and can be considered as the liver lobule's "pseudospace."

CellUntangler can be used for counterfactual reasoning by setting $\mathbf{z}_2^1$ of cells to represent central (e.g., $\mathbf{z}_2^1 = 2.6$) or portal (e.g., $\mathbf{z}_2^1 = -2.6$) regions and using the CellUntangler decoders to estimate the expression of genes. For each gene, we calculated the ratio of its expression in the central region to the total expression in both central and portal regions. This ratio was used to classify genes as central or portal markers (Figure 6G). For the 50 known marker genes used for CellUntangler analysis, the calculated ratios perfectly classified these 50 genes as central or portal markers, consistent with the ground-truth classification from a mixed-effects model[43] (*x* axis: the μ parameter from the model, where negative values indicate central markers and positive values indicate portal markers; Figure 6G). Using these ratios, we identified 44 central genes (ratio < 0.2) and 25 portal genes (ratio > 0.8) and used these genes to calculate gene-signature scores for each cell. These gene-signature scores showed gradients along the $\mathbf{z}_2^1$ axis (Figure 6H). Based on the CellUntangler-decoded expression, we identified genes that were upregulated in the starvation state (Figure 6I). The top two genes, *Cyp4a10* and *Cyp4a14*, are known to play central roles in the ω-oxidation of fatty acids and have previously been shown to be upregulated in fasting.[46]

CellUntangler effectively captures and removes diverse biological signals and scales to large datasets

We next used CellUntangler to analyze mast cells in the context of eosinophilic esophagitis (EoE), a chronic inflammatory disease of the esophagus.[47] Mast cells are enriched in the esophageal mucosa of patients with active EoE compared to healthy individuals. However, as tissue-resident cells, mast cells also exhibit a strong tissue-dissociation signal, complicating the analysis of their function. Here, we used CellUntangler to capture and remove the tissue-dissociation signal using a set of 34 tissue-dissociation signature genes[48] (Table S1). We used $\mathbf{z}^1 \in \mathbb{E}^2$ to capture the tissue-dissociation signal and $\mathbf{z}^2 \in \mathbb{E}^{10}$ to capture other signals such as disease. As expected, we observed that $\mathbf{z}^1$ was correlated with the tissue-dissociation signature score[49] (calculated by Scanpy) (Figure S6A). In $\mathbf{z}^2$, cells were clearly grouped by disease status but not by the tissue-dissociation signal (Figure S6B). In contrast, using the standard pipeline, with batch correction on version (10x Chemistry) using Harmony,[50] we did not observe this pattern because of the strong tissue-dissociation signal in the dataset (Figure S6C). We next wanted to detect the upregulated genes in mast cells from active EoE patients compared to those from healthy individuals.

Using $\mathbf{z}^2$ and CellUntangler's decoder, we obtained the top 20 genes upregulated in active cells compared to healthy cells (Figure S6D; STAR Methods). These top upregulated genes include *ITGA2B*. *ITGA2B* is a well-known marker of megakaryocytes/platelets. It has also been shown to be expressed in bone marrow-derived mast cells and modulates chronic inflammation.[51] We used the top 20 genes obtained with CellUntangler to compute "mast cell activation" signature scores[49] in individual cells. We found that mast cells from active EoE patients upregulated this gene signature compared to those from healthy or remission patients (Figure S6E). Similarly, *ITGA2B* expression was higher in active EoE patients compared to healthy or remission patients (Figure S6F).

Furthermore, we examined a dataset of peripheral blood mononuclear cells (PBMCs) stimulated with IFN-β.[52] By filtering out the cell-type signal from the PBMCs, we can focus on studying the differences between control and stimulated cells across cell types. We used cell-type marker genes[31,53] (Table S1) to separate the cell-type and stimulation signals into different components, $\mathbf{z}^1 \in \mathbb{E}^{10}$ and $\mathbf{z}^2 \in \mathbb{E}^5$, respectively. We visualized $\mathbf{z}^1$ (Figure S7A) and $\mathbf{z}^2$ (Figure S7B) to confirm that the cell-type and stimulation signals were separately captured. The *k*-NN accuracies on cell type using $\mathbf{z}^1$ were >0.85 across *k*s (Figure S7C). IFN-β stimulation activates the JAK-STAT signaling pathway in cells. However, this can be obscured by other factors (e.g., cell

(C and D) (C) The embeddings of the first component, $\mathbf{z}^1$, colored by *Cyp2f2* and *Cyp2e1* expression and (D) colored by time.
(E) The embeddings of the second component, $\mathbf{z}^2$, projected to 2D using UMAP.
(F) Gene expression of *Elovl3* across zonation (left) and circadian time point (right).
(G) Gene expression of *Pck1* across zonation (left) and circadian time point (right).

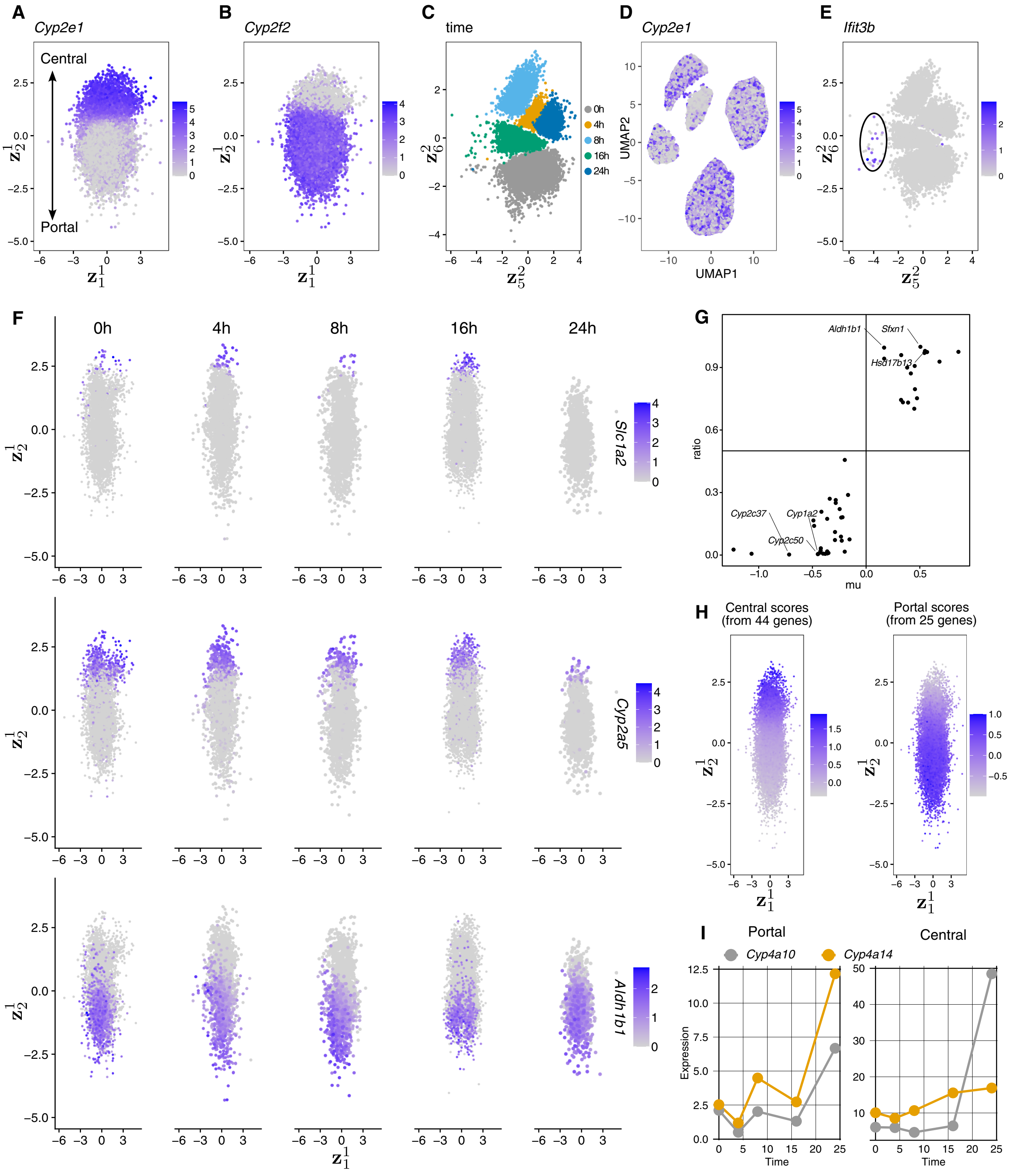

Figure 6. CellUntangler analysis of hepatocytes from mice in fed, fasted, and starvation states

(A and B) (A) We use $\mathbf{z}^1$ to capture hepatocyte spatial-zonation information (from portal to central regions) and $\mathbf{z}^2$ to capture other signals, such as time after feeding. The second coordinate ($\mathbf{z}^1_2$) of $\mathbf{z}^1$ captures the spatial zonation, as shown by the expression of central marker gene *Cyp2e1* and (B) the portal marker gene *Cyp2f2*.

(C) The fifth and sixth coordinates of $\mathbf{z}^2$ capture the temporal information.

(D) The second component $\mathbf{z}^2$ UMAP is colored by *Cyp2e1* expression.

(legend continued on next page)

types), resulting in control and stimulated cells having similar JAK-STAT scores (mean of −0.111 in the unstimulated cells and 0.0972 in the stimulated cells), although statistically significantly different (Mann-Whitney U test p value [two-sided]: 4.619e−252) (Figure S7D). We used CellUntangler to obtain a gene reconstruction with the cell-type signal filtered out by passing $\mathbf{z}^2$ to CellUntangler's decoder. Using this reconstruction, the stimulated cells had a JAK-STAT score[54] more noticeably higher than the control cells (Figure S7E) (mean of −0.587 in the unstimulated cells and 0.516 in the stimulated cells; Mann-Whitney U test p value [two-sided]: 0.0).

We then examined the genes upregulated due to IFN-β stimulation using $\mathbf{z}^2$ (STAR Methods). One-hundred control cells and 100 stimulated cells were grouped into 100 pairs, and we subtracted the reconstructed control expression from the reconstructed stimulated expression for each pair. We obtained the top 50 genes with the largest positive difference for each pair and counted the frequency of these genes. As expected, genes such as *ISG20* and *IFIT1* were present in the top 50 genes with the highest frequency (Figure 6F).

Finally, to assess the efficiency and scalability of CellUntangler, we used a large high-grade serous ovarian cancer dataset[55] with 927,205 cells and 3,038 genes. We subsampled an increasing number of cells (i.e., 5,000, 10,000, 20,000, 40,000, 80,000, 160,000, 500,000, 750,000, 927,205). For subsamples with fewer than 100,000 cells, we ran CellUntangler for 500 epochs. As datasets with a large number of cells may require fewer epochs, for subsamples with more than 100,000 cells, we ran CellUntangler for 50 epochs. We measured the runtime of CellUntangler on a central processing unit (CPU) (Figure 7A) and an A100 graphics processing unit (GPU) (Figure 7B). The average runtime, over three runs, was 264.16 min on CPU and 94.84 min on the GPU (STAR Methods).

We found that CellUntangler achieved strong performance even after only 50 epochs on the full dataset (Figures 7C and 7D). For the experiments, we used CellUntangler to capture and remove the IFN-response signal in the ovarian cancer tumor microenvironment with a set of 65 upregulated genes in human PBMCs after IFN-β stimulation[52] (Table S1). We used the Euclidean space of dimension two to capture the IFN-β response signal, $\mathbf{z}^1 \in \mathbb{E}^2$, and the Euclidean space of dimension 10 to capture the cell type, $\mathbf{z}^2 \in \mathbb{E}^{10}$. As expected, the first component, $\mathbf{z}^1$, captured the IFN-response signal (Figure 7C), while the second component, $\mathbf{z}^2$, captured the cell type (Figure 7D). Visualization of $\mathbf{z}^1$ by individual cell type further revealed that different cell types within the tumor microenvironment exhibited IFN-β responses (Figure 7E). These results suggest that a broader range of cell types, beyond the lymphocytes originally reported,[55] respond to IFN stimulation in this context.

DISCUSSION

We introduced CellUntangler, a deep generative model designed to capture and separate biological signals in cells by embedding them into a decomposed latent space, where each subspace can be Euclidean, hyperspherical, or hyperbolic. CellUntangler allows us to obtain separate embeddings for each signal, and we demonstrated its effectiveness on datasets consisting of the cell-cycle signal along with genetic or cell-type differences or differentiation trajectories, zonation and circadian signals, cell dissociation effects and disease states, or IFN response and cell types.

CellUntangler works well on datasets composed of only cycling cells. On the HeLa cell dataset,[29] it successfully separated cells by cell-cycle stage and by genotype, and, when comparing k-NN accuracies, it showed favorable performance for smaller values of k and comparable results for larger values. Although Revelio[29] performed similarly well, it required more cycling genes and prior knowledge of the cell-cycle stage for each marker gene. CellUntangler only requires knowledge of the marker genes for the signal. scPrisma,[10] which does not require any prior knowledge of marker genes, demands more hyperparameter tuning for the test dataset. On the dataset of mouse embryonic stem cells,[6] the cells proceeded in the order of pseudotime, while no such ordering was observed in the second latent subspace. Visualizing the data in the Poincaré disk also provides a convenient measure of pseudotime, based on the angle from the origin. We found that our pseudotime estimation correlated well with the ground truth, demonstrating the effectiveness of the hyperbolic space with the RoWN[28] in capturing the cell-cycle signal. A key strength of CellUntangler is its ability to handle both cycling and non-cycling cells within the same dataset. Other methods may struggle in this regard, as they typically assume all cells follow the cell-cycle trajectory.[9,10] CellUntangler, however, distinguishes cycling cells from non-cycling cells by positioning cycling cells along the edge of the Poincaré disk.[22] In addition, CellUntangler performs well in specifically capturing signals of interest, enabling the identification of rare cycling astrocytes alongside other cycling microglia in mouse retinal cells.[38] In the mouse liver hepatocyte datasets, CellUntangler accurately recovered the spatial zonation signal in $\mathbf{z}^1$, which can be considered as pseudospace and can be useful for studying development and tissue biology.[34] In the high-grade serous ovarian cancer dataset,[55] CellUntangler revealed that a broad range of cell

(E) Notably, a small set of hepatocytes expressing IFN-response genes is readily detected but missed in the original study.
(F) We also show the expression of central marker genes *Slc1a2* and *Cyp2a5*, and the portal marker gene *Aldh1b1*, in hepatocytes from mice in the fed (0 h), fasted (4 h, 8 h, and 16h), and starvation (24 h) states. Counterfactual gene expression predicted by CellUntangler (by setting $\mathbf{z}_2^1$ to portal or central values) enables computation of central vs portal expression ratios.
(G) These ratios recapitulate known zonation markers (based on mixed-effects modeling); x axis: mu, negative values indicate central markers, positive values indicate portal marker; these are used to classify central (ratio < 0.2) and portal (ratio > 0.8) genes.
(H) Gene signature scores from 44 predicted central and 25 predicted portal genes show consistent gradients along $\mathbf{z}_2^1$.
(I) Counterfactual decoding identifies genes upregulated in starvation, with top two genes *Cyp4a10* and *Cyp4a14*, which are key enzymes in fatty acid ω-oxidation and are known to be induced by fasting.

CellPress
OPEN ACCESS

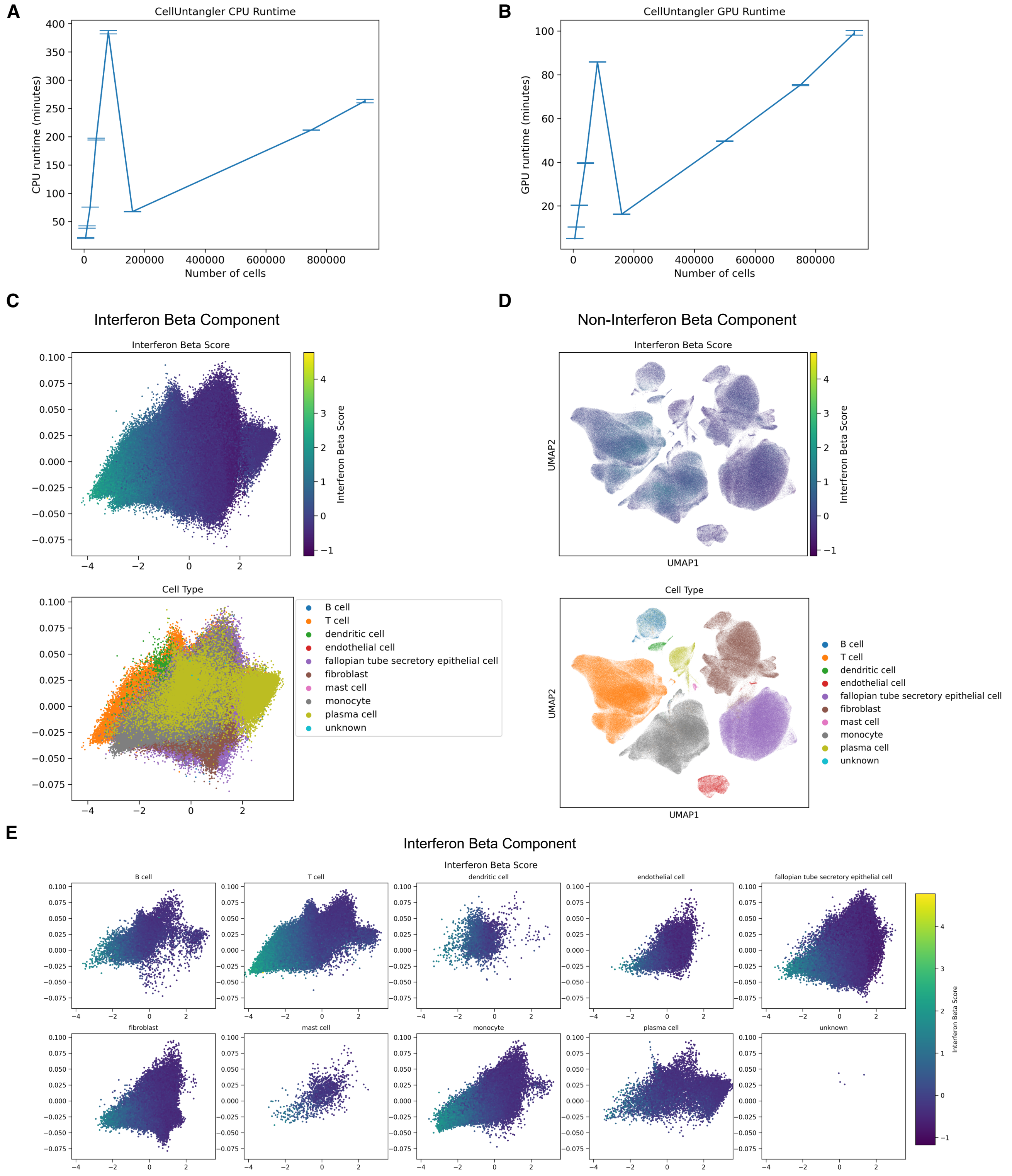

Figure 7. CellUntangler identifies broad cell types of the high-grade serous ovarian cancer tumor microenvironment that respond to IFN and are scalable to large numbers of cells

(A and B) (A) The average runtime over three runs of CellUntangler on CPU and (B) an A100 GPU. Datasets with less than 100,000 cells were run for 500 epochs, and datasets with more than 100,000 cells were run for 50 epochs.

(C and D) (C) The embeddings of the first component, $\mathbf{z}^1$, and (D) the embeddings of the second component, $\mathbf{z}^2$, projected to 2D using UMAP.

(E) The embeddings of the first component, $\mathbf{z}^1$, plotted individually by cell type.

types of the tumor microenvironment respond to IFN beyond just lymphocytes reported in the original study.[55]

Importantly, CellUntangler captures multiple signals in simultaneous representations that capture and filter out the desired signals, reducing the number of steps required, and the second component can retain relevant cell information while filtering out the prominent effects of another process, such as the cell cycle, zonation,[43,45] or cell dissociation signal.[47] In the immune cell dataset,[37] cell-type information is preserved, allowing for accurate identification of the cell types of cycling cells, which can help interpret scRNA-seq datasets. We can readily use CellUntangler to identify the origin of heterogeneous cell populations, e.g., migratory dendritic cells, thereby facilitating biological discovery. In the pancreatic cell dataset,[39] it captures a differentiation trajectory. In the immune cell dataset, it also addresses batch effects, ensuring that irrelevant information is not captured in the latent spaces, eliminating the need for a separate step to remove such noise.

Crucially, CellUntangler can distinguish, capture, and filter signals other than the cell cycle, such as zonation and circadian rhythms in the mouse liver cell dataset,[43,45] and only knowledge of some marker genes for the primary signal is required. Additionally, CellUntangler can be extended to use more complex latent spaces with multiple subspaces. For example, the cell cycle could be represented by embedding cells along a cylinder, the product space of a circle and a line. Analyses with markers for organ-specific features and multiple subspaces could also be used to tackle variation from the organ in the immune cells dataset. As a deep generative model, CellUntangler is scalable to process large datasets. We anticipate that CellUntangler will be a powerful tool for extracting and filtering various signals for downstream analysis, and its flexibility in latent space will enable us to gain deep insights into single-cell data.

Limitations of the study

Compared to unsupervised methods, CellUntangler requires a set of marker genes for each signal as input. The marker gene sets used in this study are provided in Table S1. However, marker genes for certain signals of interest may not be readily available. In such cases, users can identify a small set of highly specific marker genes through differential-expression analysis or a literature search, potentially augmented with larger language models. In the future, we plan to compile and release more marker gene sets for broader use. In this study, we focused on scRNA-seq data analysis; applications of CellUntangler to other single-cell data modalities, such as scATAC-seq, spatial transcriptomics data, or multi-omics data,[31,56,57] has not been explored and represent promising directions for future research.

RESOURCE AVAILABILITY

Lead contact

Further information and requests for resources should be directed to and will be fulfilled by the lead contact, Jiarui Ding (jiarui.ding@ubc.ca).

Materials availability

This study did not generate new unique reagents.

Data and code availability

- All data used in this study is publicly available. The datasets used are available through their sources in the key resources table.
- All original code has been deposited in GitHub (https://github.com/Ding-Group/CellUntangler) and achieved in Zenodo (https://doi.org/10.5281/zenodo.17375139).
- Any additional information required to reanalyze the data reported in this paper is available from the lead contact upon request.

ACKNOWLEDGMENTS

This work was supported by a Discovery grant from the Natural Sciences and Engineering Research Council of Canada (NSERC) and a department startup fund from the University of British Columbia (to J.D.) as well as the Klarman Cell Observatory (A.R.). J.D. is a Canada Research Chair and is supported by the Canadian Institutes of Health Research through the Canada Research Chair Program. The computational resource is partially supported by the Canada Foundation for Innovation and John. R. Evans Leader Fund (to J.D.). This research was supported in part through the computational resources and services provided by Advanced Research Computing at the University of British Columbia.

AUTHOR CONTRIBUTIONS

J.D. and A.R. conceived the study and developed the prototype model. J.D. and S.C. developed the model. S.C. conducted experimental analyses and interpreted the results with supervision from J.D. and A.C. S.C., J.D., A.R., and A.C. wrote the manuscript.

DECLARATION OF INTERESTS

A.R. is an employee of Genentech, a member of the Roche Group, and has equity in Roche. A.R. is a co-founder and equity holder of Celsius Therapeutics, an equity holder in Immunitas, and until 31 July 2020 was on the scientific advisory board of Thermo Fisher Scientific, Syros Pharmaceuticals, Neogene Therapeutics, and Asimov.

STAR★METHODS

Detailed methods are provided in the online version of this paper and include the following:

- KEY RESOURCES TABLE
- METHOD DETAILS
  - The CellUntangler model
  - The rotated hyperbolic wrapped normal distribution
  - Recentering the origin
  - The general CellUntangler model
  - Model structure
  - Cell-cycle genes and other marker genes
  - Cell-cycle pseudotime
  - Cell-cycle reconstruction
  - Cell identity of cycling cells
  - Batch effect removal
  - Discovering upregulated genes
  - Parameter setting for other methods
  - Datasets

SUPPLEMENTAL INFORMATION

Supplemental information can be found online at https://doi.org/10.1016/j.xgen.2025.101073.

Received: January 23, 2025
Revised: August 12, 2025
Accepted: October 28, 2025
Published: December 1, 2025

REFERENCES

1. Stegle, O., Teichmann, S.A., and Marioni, J.C. (2015). Computational and analytical challenges in single-cell transcriptomics. Nat. Rev. Genet. *16*, 133–145. https://doi.org/10.1038/nrg3833.
2. Buettner, F., Natarajan, K.N., Casale, F.P., Proserpio, V., Scialdone, A., Theis, F.J., Teichmann, S.A., Marioni, J.C., and Stegle, O. (2015). Computational analysis of cell-to-cell heterogeneity in single-cell RNA-sequencing data reveals hidden subpopulations of cells. Nat. Biotechnol. *33*, 155–160. https://doi.org/10.1038/nbt.3102.
3. Barron, M., and Li, J. (2016). Identifying and removing the cell-cycle effect from single-cell RNA-Sequencing data. Sci. Rep. *6*, 33892. https://doi.org/10.1038/srep33892.
4. Kowalczyk, M.S., Tirosh, I., Heckl, D., Rao, T.N., Dixit, A., Haas, B.J., Schneider, R.K., Wagers, A.J., Ebert, B.L., and Regev, A. (2015). Single-cell RNA-seq reveals changes in cell cycle and differentiation programs upon aging of hematopoietic stem cells. Genome Res. *25*, 1860–1872. https://doi.org/10.1101/gr.192237.115.
5. Zheng, S.C., Stein-O'Brien, G., Augustin, J.J., Slosberg, J., Carosso, G.A., Winer, B., Shin, G., Bjornsson, H.T., Goff, L.A., and Hansen, K.D. (2022). Universal prediction of cell-cycle position using transfer learning. Genome Biol. *23*, 41. https://doi.org/10.1186/s13059-021-02581-y.
6. Riba, A., Oravecz, A., Durik, M., Jiménez, S., Alunni, V., Cerciat, M., Jung, M., Keime, C., Keyes, W.M., and Molina, N. (2022). Cell cycle gene regulation dynamics revealed by RNA velocity and deep-learning. Nat. Commun. *13*, 2865. https://doi.org/10.1038/s41467-022-30545-8.
7. Liu, Z., Lou, H., Xie, K., Wang, H., Chen, N., Aparicio, O.M., Zhang, M.Q., Jiang, R., and Chen, T. (2017). Reconstructing cell cycle pseudo time-series via single-cell transcriptome data. Nat. Commun. *8*, 22. https://doi.org/10.1038/s41467-017-00039-z.
8. Moussa, M., and Măndoiu, I.I. (2021). Computational Cell Cycle Analysis of Single Cell RNA-seq Data. In Computational Advances in Bio and Medical Sciences (Springer International Publishing), pp. 71–87. https://doi.org/10.1007/978-3-030-79290-9_7.
9. Liang, S., Wang, F., Han, J., and Chen, K. (2020). Latent periodic process inference from single-cell RNA-seq data. Nat. Commun. *11*, 1441. https://doi.org/10.1038/s41467-020-15295-9.
10. Karin, J., Bornfeld, Y., and Nitzan, M. (2023). scPrisma infers, filters and enhances topological signals in single-cell data using spectral template matching. Nat. Biotechnol. *41*, 1645–1654. https://doi.org/10.1038/s41587-023-01663-5.
11. Piran, Z., and Nitzan, M. (2024). SiFT: uncovering hidden biological processes by probabilistic filtering of single-cell data. Nat. Commun. *15*, 760. https://doi.org/10.1038/s41467-024-44757-7.
12. Kingma, D.P., and Welling, M. (2014). Auto-encoding variational Bayes. In International Conference on Learning Representations. https://arxiv.org/abs/1312.6114.
13. Rezende, D.J., Mohamed, S., and Wierstra, D. (2014). Stochastic backpropagation and approximate inference in deep generative models. In In: Xing E.P., Jebara T., editors. Proceedings of the 31st International Conference on Machine Learning. PMLR, pp. 1278–1286.
14. Kingma, D.P., Rezende, D.J., Mohamed, S., and Welling, M. (2014). Semi-supervised learning with deep generative model. In In: Ghahramani Z., Welling M., Cortes C., Lawrence N., Weinberger K.Q., editors. Advances in Neural Information Processing Systems (Curran Associates, Inc.), pp. 3581–3589.
15. Ding, J., Condon, A., and Shah, S.P. (2018). Interpretable dimensionality reduction of single cell transcriptome data with deep generative models. Nat. Commun. *9*, 2002. https://doi.org/10.1038/s41467-018-04368-5.
16. Lopez, R., Regier, J., Cole, M.B., Jordan, M.I., and Yosef, N. (2018). Deep generative modeling for single-cell transcriptomics. Nat. Methods *15*, 1053–1058. https://doi.org/10.1038/s41592-018-0229-2.
17. Wang, D., and Gu, J. (2018). VASC: dimension reduction and visualization of single-cell RNA-seq data by deep variational autoencoder. Genom. Proteom. Bioinform. *16*, 320–331. https://doi.org/10.1016/j.gpb.2018.08.003.
18. Lotfollahi, M., Wolf, F.A., and Theis, F.J. (2019). scGen predicts single-cell perturbation responses. Nat. Methods *16*, 715–721. https://doi.org/10.1016/j.gpb.2018.08.003.
19. Grønbech, C.H., Vording, M.F., Timshel, P.N., Sønderby, C.K., Pers, T.H., and Winther, O. (2020). scVAE: variational auto-encoders for single-cell gene expression data. Bioinformatics *36*, 4415–4422. https://doi.org/10.1093/bioinformatics/btaa293.
20. Wang, Z., Yeo, G.H., Sherwood, R., and Gifford, D. (2019). Disentangled representations of cellular identity. In Research in Computational Molecular Biology, L.J. Cowen, ed. (Springer International Publishing), pp. 256–271. https://doi.org/10.1007/978-3-030-17083-7_16.
21. Davidson, T.R., Falorsi, L., De Cao, N., Kipf, T., and Tomczak, J.M. (2018). Hyperspherical variational auto-encoders. In Proceedings of the 34th Conference on Uncertainty in Artificial Intelligence, A. Globerson and R. Silva, eds. (AUAI Press Corvallis). https://auai.org/uai2018/proceedings/papers/309.pdf.
22. Nagano, Y., Yamaguchi, S., Fujita, Y., and Koyama, M. (2019). A wrapped normal distribution on hyperbolic space for gradient-based learning. In Proceedings of the 36th International Conference on Machine Learning. PMLR, K. Chaudhuri and R. Salakhutdinov, eds., pp. 4693–4702. https://proceedings.mlr.press/v97/nagano19a/nagano19a.pdf.
23. Ding, J., and Regev, A. (2021). Deep generative model embedding of single-cell RNA-Seq profiles on hyperspheres and hyperbolic spaces. Nat. Commun. *12*, 2554. https://doi.org/10.1038/s41467-021-22851-4.
24. Klimovskaia, A., Lopez-Paz, D., Bottou, L., and Nickel, M. (2020). Poincaré maps for analyzing complex hierarchies in single-cell data. Nat. Commun. *11*, 2966. https://doi.org/10.1038/s41467-020-16822-4.
25. Whitfield, M.L., Sherlock, G., Saldanha, A.J., Murray, J.I., Ball, C.A., Alexander, K.E., Matese, J.C., Perou, C.M., Hurt, M.M., Brown, P.O., and Botstein, D. (2002). Identification of genes periodically expressed in the human cell cycle and their expression in tumors. Mol. Biol. Cell *13*, 1977–2000. https://doi.org/10.1091/mbc.02-02-0030.
26. Fernandez, M.A., Rueda, C., and Peddada, S.D. (2012). Identification of a core set of signature cell cycle genes whose relative order of time to peak expression is conserved across species. Nucleic Acids Res. *40*, 2823–2832. https://doi.org/10.1093/nar/gkr1077.
27. Hodis, E., Torlai Triglia, E., Kwon, J.Y.H., Biancalani, T., Zakka, L.R., Parkar, S., Hütter, J.-C., Buffoni, L., Delorey, T.M., Phillips, D., et al. (2022). Stepwise-edited, human melanoma models reveal mutations' effect on tumor and microenvironment. Science *376*, eabi8175. https://doi.org/10.1126/science.abi8175.
28. Cho, S., Lee, J., Park, J., and Kim, D. (2022). A rotated hyperbolic wrapped normal distribution for hierarchical representation learning. In Advances in Neural Information Processing Systems. Curran Associates, Inc., A.H. Oh, A. Agarwal, D. Belgrave, and K. Cho, eds., pp. 17831–17843. https://proceedings.neurips.cc/paper_files/paper/2022/file/71ad539a57b1fd49b19e5c80070cb8b9-Paper-Conference.pdf.
29. Schwabe, D., Formichetti, S., Junker, J.P., Falcke, M., and Rajewsky, N. (2020). The transcriptome dynamics of single cells during the cell cycle. Mol. Syst. Biol. *16*, e9946. https://doi.org/10.15252/msb.20209946.
30. Hao, Y., Stuart, T., Kowalski, M.H., Choudhary, S., Hoffman, P., Hartman, A., Srivastava, A., Molla, G., Madad, S., Fernandez-Granda, C., and Satija, R. (2024). Dictionary learning for integrative, multimodal and scalable single-cell analysis. Nat. Biotechnol. *42*, 293–304. https://doi.org/10.1038/s41587-023-01767-y.
31. Hao, Y., Hao, S., Andersen-Nissen, E., Mauck, W.M., 3rd, Zheng, S., Butler, A., Lee, M.J., Wilk, A.J., Darby, C., Zager, M., et al. (2021). Integrated analysis of multimodal single-cell data. Cell *184*, 3573–3587.e29. https://doi.org/10.1016/j.cell.2021.04.048.

32. Stuart, T., Butler, A., Hoffman, P., Hafemeister, C., Papalexi, E., Mauck, W.M., 3rd, Hao, Y., Stoeckius, M., Smibert, P., and Satija, R. (2019). Comprehensive integration of single-cell data. Cell *177*, 1888–1902.e21. https://doi.org/10.1016/j.cell.2019.05.031.

33. Butler, A., Hoffman, P., Smibert, P., Papalexi, E., and Satija, R. (2018). Integrating single-cell transcriptomic data across different conditions, technologies, and species. Nat. Biotechnol. *36*, 411–420. https://doi.org/10.1038/nbt.4096.

34. Satija, R., Farrell, J.A., Gennert, D., Schier, A.F., and Regev, A. (2015). Spatial reconstruction of single-cell gene expression data. Nat. Biotechnol. *33*, 495–502. https://doi.org/10.1038/nbt.3192.

35. Wolf, F.A., Angerer, P., and Theis, F.J. (2018). SCANPY: large-scale single-cell gene expression data analysis. Genome Biol. *19*, 15. https://doi.org/10.1186/s13059-017-1382-0.

36. Luecken, M.D., Büttner, M., Chaichoompu, K., Danese, A., Interlandi, M., Müller, M.F., Strobl, D.C., Zappia, L., Dugas, M., Colomé-Tatché, M., and Theis, F.J. (2022). Benchmarking atlas-level data integration in single-cell genomics. Nat. Methods *19*, 41–50. https://doi.org/10.1038/s41592-021-01336-8.

37. Domínguez Conde, C., Xu, C., Jarvis, L.B., Rainbow, D.B., Wells, S.B., Gomes, T., Howlett, S.K., Suchanek, O., Polanski, K., King, H.W., et al. (2022). Cross-tissue immune cell analysis reveals tissue-specific features in humans. Science *376*, eabl5197. https://doi.org/10.1126/science.abl5197.

38. Benhar, I., Ding, J., Yan, W., Whitney, I.E., Jacobi, A., Sud, M., Burgin, G., Shekhar, K., Tran, N.M., Wang, C., et al. (2023). Temporal single-cell atlas of non-neuronal retinal cells reveals dynamic, coordinated multicellular responses to central nervous system injury. Nat. Immunol. *24*, 700–713. https://doi.org/10.1038/s41590-023-01437-w.

39. Bastidas-Ponce, A., Tritschler, S., Dony, L., Scheibner, K., Tarquis-Medina, M., Salinno, C., Schirge, S., Burtscher, I., Böttcher, A., Theis, F.J., et al. (2019). Comprehensive single cell mRNA profiling reveals a detailed roadmap for pancreatic endocrinogenesis. Development *146*, dev173849. https://doi.org/10.1242/dev.173849.

40. Bergen, V., Lange, M., Peidli, S., Wolf, F.A., and Theis, F.J. (2020). Generalizing RNA velocity to transient cell states through dynamical modeling. Nat. Biotechnol. *38*, 1408–1414. https://doi.org/10.1038/s41587-020-0591-3.

41. Baron, M., Veres, A., Wolock, S.L., Faust, A.L., Gaujoux, R., Vetere, A., Ryu, J.H., Wagner, B.K., Shen-Orr, S.S., Klein, A.M., et al. (2016). A single-cell transcriptomic map of the human and mouse pancreas reveals inter- and intra-cell population structure. Cell Syst. *3*, 346–360.e4. https://doi.org/10.1016/j.cels.2016.08.011.

42. Lange, M., Bergen, V., Klein, M., Setty, M., Reuter, B., Bakhti, M., Lickert, H., Ansari, M., Schniering, J., Schiller, H.B., et al. (2022). CellRank for directed single-cell fate mapping. Nat. Methods *19*, 159–170. https://doi.org/10.1038/s41592-021-01346-6.

43. Droin, C., Kholtei, J.E., Bahar Halpern, K., Hurni, C., Rozenberg, M., Muvkadi, S., Itzkovitz, S., and Naef, F. (2021). Space-time logic of liver gene expression at sub-lobular scale. Nat. Metab. *3*, 43–58. https://doi.org/10.1038/s42255-020-00323-1.

44. Ben-Moshe, S., and Itzkovitz, S. (2019). Spatial heterogeneity in the mammalian liver. Nat. Rev. Gastroenterol. Hepatol. *16*, 395–410. https://doi.org/10.1038/s41575-019-0134-x.

45. Okada, J., Landgraf, A., Xiaoli, A.M., Liu, L., Horton, M., Schuster, V.L., Yang, F., Sidoli, S., Qiu, Y., Kurland, I.J., et al. (2025). Spatial hepatocyte plasticity of gluconeogenesis during the metabolic transitions between fed, fasted and starvation states. Nat. Metab. *7*, 1073–1091. https://doi.org/10.1038/s42255-025-01269-y.

46. Patsouris, D., Reddy, J.K., Müller, M., and Kersten, S. (2006). Peroxisome proliferator-activated receptorα mediates the effects of high-fat diet on hepatic gene expression. Endocrinology *147*, 1508–1516. https://doi.org/10.1210/en.2005-1132.

47. Ding, J., Garber, J.J., Uchida, A., Lefkovith, A., Carter, G.T., Vimalathas, P., Canha, L., Dougan, M., Staller, K., Yarze, J., et al. (2024). An esophagus cell atlas reveals dynamic rewiring during active eosinophilic esophagitis and remission. Nat. Commun. *15*, 3344. https://doi.org/10.1038/s41467-024-47647-0.

48. van den Brink, S.C., Sage, F., Vértesy, Á., Spanjaard, B., Peterson-Maduro, J., Baron, C.S., Robin, C., and van Oudenaarden, A. (2017). Single-cell sequencing reveals dissociation-induced gene expression in tissue subpopulations. Nat. Methods *14*, 935–936. https://doi.org/10.1038/nmeth.4437.

49. Tirosh, I., Izar, B., Prakadan, S.M., Wadsworth, M.H., 2nd, Treacy, D., Trombetta, J.J., Rotem, A., Rodman, C., Lian, C., Murphy, G., et al. (2016). Dissecting the multicellular ecosystem of metastatic melanoma by single-cell RNA-seq. Science *352*, 189–196. https://doi.org/10.1126/science.aad0501.

50. Korsunsky, I., Millard, N., Fan, J., Slowikowski, K., Zhang, F., Wei, K., Baglaenko, Y., Brenner, M., Loh, P.R., and Raychaudhuri, S. (2019). Fast, sensitive and accurate integration of single-cell data with Harmony. Nat. Methods *16*, 1289–1296. https://doi.org/10.1038/s41592-019-0619-0.

51. Oki, T., Eto, K., Izawa, K., Yamanishi, Y., Inagaki, N., Frampton, J., Kitamura, T., and Kitaura, J. (2009). Evidence that integrin αIIbβ3-dependent interaction of mast cells with fibrinogen exacerbates chronic inflammation. J. Biol. Chem. *284*, 31463–31472. https://doi.org/10.1074/jbc.M109.030213.

52. Kang, H.M., Subramaniam, M., Targ, S., Nguyen, M., Maliskova, L., McCarthy, E., Wan, E., Wong, S., Byrnes, L., Lanata, C.M., et al. (2018). Multiplexed droplet single-cell RNA-sequencing using natural genetic variation. Nat. Biotechnol. *36*, 89–94. https://doi.org/10.1038/nbt.4042.

53. Hu, C., Li, T., Xu, Y., Zhang, X., Li, F., Bai, J., Chen, J., Jiang, W., Yang, K., Ou, Q., et al. (2023). CellMarker 2.0: an updated database of manually curated cell markers in human/mouse and web tools based on scRNA-seq data. Nucleic Acids Res. *51*, D870–D876. https://doi.org/10.1093/nar/gkac947.

54. Hu, X., Li, J., Fu, M., Zhao, X., and Wang, W. (2021). The JAK/STAT signaling pathway: from bench to clinic. Signal Transduct. Target. Ther. *6*, 402. https://doi.org/10.1038/s41392-021-00791-1.

55. Vázquez-García, I., Uhlitz, F., Ceglia, N., Lim, J.L.P., Wu, M., Mohibullah, N., Niyazov, J., Ruiz, A.E.B., Boehm, K.M., Bojilova, V., et al. (2022). Ovarian cancer mutational processes drive site-specific immune evasion. Nature *612*, 778–786. https://doi.org/10.1038/s41586-022-05496-1.

56. Cao, Z.-J., and Gao, G. (2022). Multi-omics single-cell data integration and regulatory inference with graph-linked embedding. Nat. Biotechnol. *40*, 1458–1466. https://doi.org/10.1038/s41587-022-01284-4.

57. Yang, X., Mann, K.K., Wu, H., and Ding, J. (2024). scCross: a deep generative model for unifying single-cell multi-omics with seamless integration, cross-modal generation, and in silico exploration. Genome Biol. *25*, 198. https://doi.org/10.1186/s13059-024-03338-z.

58. Skopek, O., Ganea, O.-E., and Bécigneul, G. (2020). Mixed-curvature variational autoencoders. In International Conference on Learning Representations. https://openreview.net/pdf?id=S1g6xeSKDS.

59. Gavish, A., Tyler, M., Greenwald, A.C., Hoefflin, R., Simkin, D., Tschernichovsky, R., Galili Darnell, N., Somech, E., Barbolin, C., Antman, T., et al. (2023). Hallmarks of transcriptional intratumour heterogeneity across a thousand tumours. Nature *618*, 598–606. https://doi.org/10.1038/s41586-023-06130-4.

60. Vieth, B., Ziegenhain, C., Parekh, S., Enard, W., and Hellmann, I. (2017). powsimR: power analysis for bulk and single cell RNA-seq experiments. Bioinformatics *33*, 3486–3488. https://doi.org/10.1093/bioinformatics/btx435.

61. Svensson, V. (2020). Droplet scRNA-seq is not zero-inflated. Nat. Biotechnol. *38*, 147–150. https://doi.org/10.1038/s41587-019-0379-5.

62. Hafemeister, C., and Satija, R. (2019). Normalization and variance stabilization of single-cell RNA-seq data using regularized negative binomial regression. Genome Biol. *20*, 296. https://doi.org/10.1186/s13059-019-1874-1.

63. Hendrycks, D., and Gimpel, K. (2016). Bridging nonlinearities and stochastic regularizers with Gaussian Error Linear Units. Preprint at arXiv. https://doi.org/10.48550/arXiv.1606.08415.

64. Glorot, X., and Bengio, Y. (2010). Understanding the difficulty of training deep feedforward neural networks. In Proceedings of the 13th International Conference on Artificial Intelligence and Statistics (PMLR), pp. 249–256. http://proceedings.mlr.press/v9/glorot10a/glorot10a.pdf.

65. Loshchilov, I., and Hutter, F. (2019). Decoupled weight decay regularization. In International Conference on Learning Representations. https://openreview.net/pdf/5963886abef941684ffc0cf670297e47fb1e5155.pdf.

66. Baldarelli, R.M., Smith, C.L., Ringwald, M., Richardson, J.E., and Bult, C.J.; Mouse Genome Informatics Group (2024). Mouse Genome Informatics: an integrated knowledgebase system for the laboratory mouse. Genetics *227*, iyae031. https://doi.org/10.1093/genetics/iyae031.

67. Macosko, E.Z., Basu, A., Satija, R., Nemesh, J., Shekhar, K., Goldman, M., Tirosh, I., Bialas, A.R., Kamitaki, N., Martersteck, E.M., et al. (2015). Highly parallel genome-wide expression profiling of individual cells using nanoliter droplets. Cell *161*, 1202–1214. https://doi.org/10.1016/j.cell.2015.05.002.

STAR★METHODS

KEY RESOURCES TABLE

REAGENT or RESOURCE	SOURCE	IDENTIFIER
Deposited data		
HeLa	Schwabe, D. et al.[29]	GEO: GSE142277
Mouse Embryonic Stem Cells	Riba, A. et al.[6]	GEO: GSE167609
Immune Cells	Conde, C. et al.[37]	https://www.tissueimmunecellatlas.org/
Mouse Retina Cells	Benhar, I. et al.[38]	GEO: GSE199317
Mouse Pancreas Cells	Bastidas-Ponce, A. et al.[39]	GEO: GSE132188
Mouse Liver Cells	Droin, C. et al.[43]	GEO: GSE145197
Mouse hepatocytes after feeding	Okada, J. et al.[45]	GEO: GSE263418
Mast Cells	Ding, J. et al.[47]	https://singlecell.broadinstitute.org/single_cell/study/SCP1242/
Peripheral Blood Mononuclear Cells	Kang, H. et al.[52]	GEO: GSE96853
Ovarian Cancer Cells	Vázquez-Garcìa, I. et al.[55]	syn25569736
Software and algorithms		
CellUntangler	This paper	https://github.com/Ding-Group/CellUntangler
Scanpy	Wolf, F. et al.[35]	https://github.com/scverse/scanpy
Tricycle	Zheng, S. et al.[5]	https://github.com/hansenlab/tricycle
scPrisma	Karin, J. et al.[10]	https://github.com/nitzanlab/scPrisma/
Revelio	Schwabe, D. et al.[29]	https://github.com/danielschw188/Revelio
Seurat	Hao, Y. et al.[30]	https://satijalab.org/seurat/
SiFT	Piran, Z. et al.[11]	https://github.com/nitzanlab/sift-sc
Scib	Luecken, M. et al.[36]	https://github.com/theislab/scib

METHOD DETAILS

The CellUntangler model

CellUntangler isolates and reveals biological processes that might obscure one another. It takes as input a gene expression count matrix and a set of marker genes for a known signal. It separates the known signal from other signals present in the dataset, by embedding each cell into a latent space consisting of several subspaces, with each subspace having the appropriate geometry to capture the signal of interest.

A scRNA-seq dataset $\mathcal{D} = \{(\mathbf{x_i}, \mathbf{y_i})\}_{i=1}^{N}$, where N is the number of cells, $\mathbf{x}_i \in \mathbb{R}^G$ is a Unique Molecular Identifier (UMI) count vector, G is the number of genes, and $\mathbf{y}_i$ is a categorical vector of the batch or batches associated with $\mathbf{x}_i$, is provided as input to CellUntangler. $\mathbf{x}_i$ is log-transformed prior to input into the model. Single-cell data intrinsically has low-dimensionality,[23] so we assume that the distribution of $\mathbf{x}_i$ is governed by a lower-dimensional vector $\mathbf{z}_i$. Accordingly, the joint distribution can be factorized as follows

$$p(\mathbf{x}_i, \mathbf{y}_i, \mathbf{z}_i|\boldsymbol{\theta}_i) = p(\mathbf{y}_i|\boldsymbol{\theta}_i)\cdot p(\mathbf{z}_i|\boldsymbol{\theta}_i)\cdot p(\mathbf{x}_i|\mathbf{y}_i, \mathbf{z}_i, \boldsymbol{\theta}_i).$$

Here, $p(\mathbf{y}_i|\boldsymbol{\theta}_i)$ is the categorical distribution, and $p(\mathbf{z}_i|\boldsymbol{\theta}_i)$ is the prior distribution for $\mathbf{z}_i$. The parameter set $\boldsymbol{\theta}_i$ represents the parameters of each distribution. Since the distributions are different, $\boldsymbol{\theta}_i$ will differ for each distribution. For example, if $p(\mathbf{z}_i|\boldsymbol{\theta}_i)$ follows a multivariate normal distribution, $\boldsymbol{\theta}_i$ would be $\boldsymbol{\mu}_i$ and $\boldsymbol{\Sigma}_i$. To capture the complex nonlinear relationship between $\mathbf{z}_i$ and $\mathbf{x}_i$, CellUntangler uses neural networks to parametrize the distribution $p(\mathbf{x}_i|\mathbf{y}_i,\mathbf{z}_i,\boldsymbol{\theta}_i)$. The use of a neural network for $p(\mathbf{x}_i|\mathbf{y}_i,\mathbf{z}_i,\boldsymbol{\theta}_i)$ makes the computation of the posterior $p(\mathbf{z}_i|\mathbf{x}_i,\boldsymbol{\theta}_i)$ intractable, so variational inference is used with the variational distribution $q(\mathbf{z}_i|\mathbf{x}_i,\boldsymbol{\phi}_i)$, an approximation of the true posterior where the variational parameters $\boldsymbol{\phi}_i$ are output by a neural network.

CellUntangler consists of two neural networks: the inference network (encoder) and the model network (decoder), forming a variational autoencoder (VAE) architecture. In a conventional VAE,[12] the latent space is typically a single space with a constant curvature, such as the Euclidean, hyperspherical,[21] or hyperbolic latent spaces.[22] Different dimensions of the latent space are treated equally, and they jointly learn representations of the input data, without explicitly encoding distinct signals into different dimensions of the latent space. In contrast, CellUntangler allows for a product of spaces, where each subspace can have a distinct curvature, either Euclidean, hyperspherical, or hyperbolic with varying dimensionality for k subspaces, as in the mixed-curvature variational autoencoder (MVAE)[58] framework. This design enables CellUntangler to potentially capture and disentangle distinct signals. To help capture biological processes of interest, e.g., cell-cycle, stress, and cell-type information, we decompose the latent vector

$\mathbf{z}_i = (\mathbf{z}_i^1, \mathbf{z}_i^2, \ldots, \mathbf{z}_i^k)$, the concatenation of vectors $\mathbf{z}_i^1$, $\mathbf{z}_i^2$, and $\mathbf{z}_i^k$, such that each compartment $\mathbf{z}_i^j$ presents a biological process, e.g., cell cycle. The posterior can now be written as $p(\mathbf{z}_i|\mathbf{x}_i, \boldsymbol{\theta}_\mathbf{i}) = p(\mathbf{z}_i^1, \mathbf{z}_i^2 \ldots, \mathbf{z}_i^k|\mathbf{x}_i, \boldsymbol{\theta}_i)$. The choice of geometry for each latent subspace is tailored to the specific signal being modeled. For example, a hyperbolic space might be selected to capture differentiation trajectories, which often exhibit tree-like structures.

To disentangle the confounding signal from other signals in the dataset, known marker genes for that signal are required as input. For many biological processes of interest, this prior knowledge (e.g., stress, interferon response)[38,59] is available or can be a set of marker genes that can be defined by differential expression analysis. For instance, cell-cycle marker genes can be obtained from databases, by literature curation (manually or with a large language model), or as genes upregulated in cycling vs. non-cycling cells (data-driven). For simplicity of discussion, $\mathbf{z}_i$ is decomposed into two latent components. However, the $\mathbf{z}_i$ in CellUntangler can be decomposed into multiple components (see the "the general CellUntangler model" subsection).

To use $\mathbf{z}_i^1$ to capture the known signal (e.g., cell cycle) and $\mathbf{z}_i^2$ to capture the other signals (e.g., cell type) that may be confounded by the known signal, $\mathbf{x}_i$ is decomposed into two UMI count vectors, one for the marker genes, $\mathbf{x}_i^1$, and one for the non-marker genes, $\mathbf{x}_i^2$. From $\mathbf{x}_i^1$ and $\mathbf{x}_i^2$, the encoder outputs the parameters of $q(\mathbf{z}_i^1|\mathbf{x}_i^1, \mathbf{x}_i^2, \boldsymbol{\phi}_i)$ and $q(\mathbf{z}_i^2|\mathbf{x}_i^1, \mathbf{x}_i^2, \boldsymbol{\phi}_i)$. When computing $q(\mathbf{z}_i^1|\mathbf{x}_i^1, \mathbf{x}_i^2, \boldsymbol{\phi}_i)$, $\mathbf{x}_i^2$ is masked (set to zero), while when computing $q(\mathbf{z}_i^2|\mathbf{x}_i^1, \mathbf{x}_i^2, \boldsymbol{\phi}_i)$, $\mathbf{x}_i^1$ is masked.

The UMI count of gene j in cell i, x_{ij}, is assumed to follow a negative-binomial distribution, based on previous work.[60–62] When decomposing $\mathbf{z}_i = (\mathbf{z}_i^1, \mathbf{z}_i^2)$ and assuming that there are m marker genes associated with the signal encoded in $\mathbf{z}_i^1$,

$$p(\mathbf{x}_i^1, \mathbf{x}_i^2|\mathbf{y}_i, \mathbf{z}_i^1, \mathbf{z}_i^2, \boldsymbol{\theta}_i) = \prod_{j=1}^{m} \mathrm{NB}\left(x_{ij}^1 \middle| \mu_{\mathbf{y}_i, \mathbf{z}_i^1}, \sigma_{\mathbf{y}_i, \mathbf{z}_i^1}\right) \prod_{j=m+1}^{G} \mathrm{NB}\left(x_{ij}^2 \middle| \mu_{\mathbf{y}_i, \mathbf{z}_i^1, \mathbf{z}_i^2}, \sigma_{\mathbf{y}_i, \mathbf{z}_i^1, \mathbf{z}_i^2}\right).$$

Only $\mathbf{z}_i^1$ is used to reconstruct $\mathbf{x}_i^1$, while both $\mathbf{z}_i^1$ and $\mathbf{z}_i^2$ are used to reconstruct $\mathbf{x}_i^2$, as $\mathbf{z}_i^1$ may be informative of $\mathbf{x}_i^2$.

Independence between the latent representations would allow better separation of biological signals into $\mathbf{z}_i^1$ and $\mathbf{z}_i^2$. However, backpropagation may cause information from $\mathbf{x}_i^2$ to be captured in $\mathbf{z}_i^1$, as $\mathbf{z}_i^1$ and $\mathbf{z}_i^2$ are not conditionally independent given $\mathbf{x}_i^2$. As the same gene may have activities related to more than one process, this matters particularly when using a larger set of marker genes, since some may be less specific to the desired signal. To obtain more independent representations, when the decoder is trained, $\mathbf{x}_i^2$ is reconstructed in two ways. The first uses $\mathbf{z}_i^1$ and $\mathbf{z}_i^2$ to reconstruct $\mathbf{x}_i^2$ (Figure S1A). The second stops the gradient for $\mathbf{z}_i^1$ so that it is fixed before reconstructing $\mathbf{x}_i^2$ (Figure S1B). The number of epochs for training with stop gradient for $\mathbf{z}_i^1$ in reconstructing $\mathbf{x}_i^2$ is a hyper-parameter in the model (see the "model structure" subsection).

To find the parameters for the inference neural network and model network that maximize the objective function, the evidence lower bounds (ELBO) is used,

$$\mathcal{L}(\boldsymbol{\theta}_i, \boldsymbol{\phi}_i) = -\mathbb{KL}(q(\mathbf{z}_i|\mathbf{x}_i, \boldsymbol{\phi}_i) \parallel p(\mathbf{z}_i|\boldsymbol{\theta}_i)) + \mathbb{E}_{q(\mathbf{z}_i|\mathbf{x}_i, \boldsymbol{\phi}_i)}[\log p(\mathbf{x}_i|\mathbf{y}_i, \mathbf{z}_i, \boldsymbol{\theta}_i)].$$

The ELBO requires sampling from the variational distribution and evaluating the density of samples from distributions of the Euclidean, hyperspherical, and hyperbolic spaces. The Euclidean space is defined as $\mathbb{E}^d = \mathbb{R}^d$ for $K = 0$ where d is the dimension and K is the curvature. The hyperspherical space has constant positive curvature and is defined as

$$\mathbb{S}_K^d = \left\{\mathbf{x} \in \mathbb{R}^{d+1} : \langle \mathbf{x}, \mathbf{x} \rangle_2 = \frac{1}{K}\right\}$$

for $K > 0$, where $\langle \mathbf{x}, \mathbf{y} \rangle_2 = \sum_{i=0}^{d} x_i y_i$ is the standard inner product. The hyperbolic space has constant negative curvature and is defined as

$$\mathbb{H}_K^d = \left\{\mathbf{x} \in \mathbb{R}^{d+1} : \langle \mathbf{x}, \mathbf{x} \rangle_\mathcal{L} = \frac{1}{K}\right\}$$

for $K < 0$ where $\langle \mathbf{x}, \mathbf{y} \rangle_\mathcal{L} = -x_0 y_0 + \sum_{i=1}^{d} x_i y_i$ is the Lorentz inner product, and the first elements $x_0 > 0$ and $y_0 > 0$.

For each of the three constant curvature spaces, a tangent vector space is defined at every point of the space, so we use wrapping to obtain the wrapped normal distribution[22] for the prior distribution $p(\mathbf{z}_i|\boldsymbol{\theta}_i)$ and the variational distribution $q(\mathbf{z}_i|\mathbf{x}_i, \boldsymbol{\phi}_i)$. To sample from the wrapped normal distribution with mean $\boldsymbol{\mu}$ and covariance matrix $\boldsymbol{\Sigma}$, denoted by $\mathcal{WN}(\boldsymbol{\mu}, \boldsymbol{\Sigma})$, the origin $\boldsymbol{\mu}_0$ for each space is defined as: For the Euclidean space, $\boldsymbol{\mu}_0 = \mathbf{0} \in \mathbb{R}^d$. For the hyperspherical and hyperbolic space, $\boldsymbol{\mu}_0 = \left(\frac{1}{\sqrt{|K|}}, 0, \ldots, 0\right) \in \mathbb{R}^{d+1}$. Next, $\tilde{\mathbf{v}} \sim \mathcal{N}(\mathbf{0}, \boldsymbol{\Sigma})$ where $\tilde{\mathbf{v}} \in \mathbb{R}^d$ is sampled, and, for the hyperspherical and hyperbolic space, concatenated so that the first element is 0, $\mathbf{v} = [0, \tilde{\mathbf{v}}] \in \mathbb{R}^{d+1}$. From the definition of the origin for each space, $\langle \boldsymbol{\mu}_0, \mathbf{v} \rangle_2 = 0$ and $\langle \boldsymbol{\mu}_0, \mathbf{v} \rangle_\mathcal{L} = 0$, therefore $\mathbf{v} \in \mathcal{T}_{\boldsymbol{\mu}_0}\mathcal{M}_K$ where $\mathcal{T}_{\boldsymbol{\mu}_0}\mathcal{M}_K$ is the tangent space at $\boldsymbol{\mu}_0$ of the manifold with curvature K. Parallel transport is used to transport $\mathbf{v}$ to the tangent space at $\boldsymbol{\mu}$: $\mathbf{u} = \mathrm{PT}_{\boldsymbol{\mu}_0 \to \boldsymbol{\mu}}(\mathbf{v}) \in \mathcal{T}_{\boldsymbol{\mu}}\mathcal{M}_K$. Finally, the exponential map is used to map $\mathbf{u}$ from the tangent space to the manifold: $\mathbf{z} = \exp_{\boldsymbol{\mu}}^K(\mathbf{u}) \in \mathcal{M}_K$.

To calculate the probability density of the samples, the reverse procedure is used. Given a sample $\mathbf{z}$, the logarithmic map is used to map the vector from the manifold to the tangent space at $\boldsymbol{\mu}$: $\mathbf{u} = \log_{\boldsymbol{\mu}}^K(\mathbf{z}) \in \mathcal{T}_{\boldsymbol{\mu}}\mathcal{M}_K$. Parallel transport is used to transport $\mathbf{u}$ from the

tangent space at $\boldsymbol{\mu}$ to the tangent space at $\boldsymbol{\mu}_0$: $\mathbf{v} = \mathrm{PT}^K_{\boldsymbol{\mu}\to\boldsymbol{\mu}_0}(\mathbf{u}) \in \mathcal{T}_{\boldsymbol{\mu}_0}\mathcal{M}_K$. The density is computed as $\log \mathcal{WN}(\mathbf{z}|\boldsymbol{\mu},\boldsymbol{\Sigma}) = \log \mathcal{N}(\mathbf{v};\boldsymbol{\mu}_0,\boldsymbol{\Sigma}) - \operatorname{logdet}\left(\frac{\delta f}{\delta \mathbf{v}}\right)$, where $f = \exp^K_{\boldsymbol{\mu}} \circ \mathrm{PT}^K_{\boldsymbol{\mu}_0\to\boldsymbol{\mu}}$. The KL divergence can be estimated using Monte Carlo integration.

The rotated hyperbolic wrapped normal distribution

When using the hyperbolic space to capture the cell-cycle signal, the rotated hyperbolic wrapped normal distribution (RoWN) is used as the variational distribution for the latent cell-cycle signal of each cell. To sample from $\mathcal{WN}(\boldsymbol{\mu},\boldsymbol{\Sigma})$, sampling must first be done from $\mathcal{N}(\mathbf{0},\boldsymbol{\Sigma})$, typically with a diagonal covariance matrix $\boldsymbol{\Sigma} = \operatorname{diag}(\boldsymbol{\sigma})$. As the covariance matrix is diagonal, the covariance structure will have principal axes parallel to the standard bases. The principal axes will also go through the origin since sampling is from a normal distribution with a mean of 0. As a result, the principal axes can be viewed as straight lines that pass through the origin, $l_{\mathbf{s}}(t) = t\mathbf{s} \in \mathbb{R}^d$, where $\mathbf{s} \in \mathbb{R}^d$ is a directional vector. Straight lines that pass through the origin become geodesics in the hyperbolic space (using the exponential map) and remain geodesics when projected to a Poincaré ball.[28] Thus, the principal axes for the diagonal covariance matrix, for the normal distribution with a mean of 0, in the Euclidean space become geodesics in the hyperbolic space. The vector $\mathbf{s}$ will become a tangent vector of the geodesic resulting from projecting the principal axis to the Poincaré ball on the projected point $\boldsymbol{\mu}$. As $\mathbf{s}$ is parallel to the standard bases and a tangent vector of the projected principal axes, the projected principal axes in the hyperbolic space are also locally parallel to the standard bases. The principal axes determine the covariance structure, so local variation can only be modeled parallel to the standard bases, instead of pointing in the radial direction. This is problematic because cycling cells should form a circle of approximately equal distance to the origin in Poincaré disk, such that their angular distance reflects their cell-cycle phase, and two cells of the same cell-cycle phase should have approximately similar angles. Variations in the radial direction of the Poincaré disk should model cell-cycle-independent factors, such as noise. In contrast, the hyperbolic space with the RoWN rotates the principal axes of the covariance matrix so that local variation can be modeled in the radial direction.

Given $\boldsymbol{\mu} \in \mathbb{H}^d$ so $\boldsymbol{\mu} \in \mathbb{R}^{d+1}$, two vectors are obtained: $\mathbf{x} = [\pm 1,\ldots,0] \in \mathbb{R}^d$, where $\pm$ is determined by the sign of the first element of $\boldsymbol{\mu}$ and $\mathbf{y} = \frac{[\mu_2,\mu_3,\ldots,\mu_{d+1}]}{\|[\mu_2,\mu_3,\ldots,\mu_{d+1}]\|} \in \mathbb{R}^d$, where μ_i is the ith element of $\boldsymbol{\mu}$. Given the two row vectors, $\mathbf{x}$ and $\mathbf{y}$, the rotation matrix is computed as:

$$\mathbf{R} = \mathbf{I} + \left(\mathbf{y}^{\mathrm{T}}\mathbf{x} - \mathbf{x}^{\mathrm{T}}\mathbf{y}\right) + \frac{\left(\mathbf{y}^{\mathrm{T}}\mathbf{x} - \mathbf{x}^{\mathrm{T}}\mathbf{y}\right)^2}{\left(1 + \langle \mathbf{x},\mathbf{y}\rangle_2\right)}.$$

$\langle \mathbf{x},\mathbf{y}\rangle_2$ is the standard inner product. Next, $\boldsymbol{\Sigma}$ is rotated to obtain $\widehat{\boldsymbol{\Sigma}} = \mathbf{R}\boldsymbol{\Sigma}\mathbf{R}^{\mathrm{T}}$. The same steps as before are followed but with sampling from $\mathcal{WN}(\boldsymbol{\mu},\widehat{\boldsymbol{\Sigma}})$ instead of $\mathcal{WN}(\boldsymbol{\mu},\boldsymbol{\Sigma})$, allowing sampling to remain efficient and probability density evaluation to remain tractable.

Recentering the origin

The origin is recentered by the Mobius addition, which for two vectors $\mathbf{x},\mathbf{y} \in \mathbb{H}^d_K$ with curvature K is defined as:

$$\mathbf{x} \oplus_K \mathbf{y} = \frac{\left(1 - 2K\langle \mathbf{x},\mathbf{y}\rangle_2 - K\|\mathbf{y}\|_2^2\right)\mathbf{x} + \left(1 + K\|\mathbf{x}\|_2^2\,\mathbf{y}\right)}{1 - 2K\langle \mathbf{x},\mathbf{y}\rangle_2 + K^2\|\mathbf{x}\|_2^2\|\mathbf{y}\|_2^2}.$$

When embedding datasets containing cycling and non-cycling cells, cycling cells are embedded along the edge of Poincaré disk, while non-cycling cells are placed through the rest of the disk. After locating cycling cells along the edge of the disk, the leftmost cycling cell is estimated to have coordinates $[x_l, y_l]^{\mathrm{T}}$, and the rightmost cycling cell to have coordinates $[x_r, y_r]^{\mathrm{T}}$. The estimated topmost cycling cell has coordinates $[x_t, y_t]^{\mathrm{T}}$, and the bottom-most cycling cell has coordinates $[x_b, y_b]^{\mathrm{T}}$. The center of cycling cells can then be estimated as $p_c = \left[x_l + \frac{(x_r - x_l)}{2}, y_b + \frac{(y_t - y_b)}{2}\right]^{\mathrm{T}}$. We recommend choosing the new origin, o_n, as the point diagonally across from p_c near the edge of the Poincaré disk, so that when a line is drawn from p_c to o_n, approximately half of the cycling cells line above and below the line. The line should be approximately perpendicular to the curve of the cycling cells.

The general CellUntangler model

We explain the general CellUntangler model. Assuming we divide the latent space into k subspaces, $\mathbf{z}_i = \left(\mathbf{z}_i^1,\mathbf{z}_i^2,\ldots,\mathbf{z}_i^k\right) \in \mathbb{R}^d$. We also decompose $\mathbf{x}_i$ into k components, $\mathbf{x}_i = \left(\mathbf{x}_i^1,\mathbf{x}_i^2,\ldots,\mathbf{x}_i^k\right) \in \mathbb{R}^G$, where G is the number of genes. Each component $\mathbf{x}_i^j$ is passed to an encoder to output the parameters for $p\left(\mathbf{z}_i^j \middle| \mathbf{x}_i^j,\boldsymbol{\theta}\right)$. The decomposition of $\mathbf{x}_i$ will be based on which signal we want $\mathbf{z}_i^j$ to capture. The final component, $\mathbf{z}_i^k$, is an exception and always captures signals not accounted for by the first k-1 latent components. For example, assuming we have three components and we want both $\mathbf{z}_i^1$ and $\mathbf{z}_i^2$ to capture the cell-cycle signal then both $\mathbf{x}_i^1$ and $\mathbf{x}_i^2$ would be the UMI counts of the cell-cycle marker genes. Non-cell-cycle-specific signals would be captured in $\mathbf{z}_i^3$. Alternatively, if we wanted $\mathbf{z}_i^1$ to capture the cell cycle and $\mathbf{z}_i^2$ to capture cell type, $\mathbf{x}_i^1$ would be the UMI counts for cell-cycle marker genes and $\mathbf{x}_i^2$ would be the UMI counts for cell-type marker genes. Again, $\mathbf{z}_i^3$ would capture any remaining signals so $\mathbf{x}_i^3$ would be the UMI counts for the remaining genes which are neither cell-cycle marker genes nor cell-type marker genes. When decomposing $\mathbf{x}_i$, for any two components, $\mathbf{x}_i^j$

and $\mathbf{x}_i^l$, the marker genes must be either the same or disjoint. In our example of capturing the cell cycle, cell type, and remaining signals, the cell-cycle marker genes would have to be disjoint from the cell-type marker genes and the remaining genes.

When using the decoder to model $\mathbf{x}^j$, we leverage the latent components specifically designed to capture the intended signal. For $\mathbf{x}_i^k$, however, all latent components are utilized. For example, assuming that both $\mathbf{z}_i^1$ and $\mathbf{z}_i^2$ are used to capture the cell cycle and $\mathbf{z}_i^3$ captures the non-cell-cycle signals, we would model $\mathbf{x}_i^1$ as $p\left(\mathbf{x}_i^1 \middle| \mathbf{z}_i^1, \mathbf{z}_i^2, \boldsymbol{\theta}\right)$. As $\mathbf{x}_i^1$ would be the same as $\mathbf{x}_i^2$, we do not model $\mathbf{x}_i^2$. $\mathbf{x}_i^3$ would be modeled as $p\left(\mathbf{x}_i^3 \middle| \mathbf{z}_i^1, \mathbf{z}_i^2, \mathbf{z}_i^3, \boldsymbol{\theta}\right)$. In our example of cell cycle, cell type, and remaining signals, we would model $\mathbf{x}_i^1$, $\mathbf{x}_i^2$, and $\mathbf{x}_i^3$ as $p\left(\mathbf{x}_i^1 \middle| \mathbf{z}_i^1, \boldsymbol{\theta}\right)$, $p\left(\mathbf{x}_i^2 \middle| \mathbf{z}_i^2, \boldsymbol{\theta}\right)$, and $p\left(\mathbf{x}_i^3 \middle| \mathbf{z}_i^1, \mathbf{z}_i^2, \mathbf{z}_i^3, \boldsymbol{\theta}\right)$.

Model structure

Due to the sparsity of single-cell data, the softmax activation function is used on the outputs of the decoder to output a vector of positive numbers that sum to one and encourage sparse outputs. The softmax outputs are multiplied by the sum of UMI counts for each cell to obtain the final means of the negative binomial distributions for that cell. The Gaussian Error Linear Unit (GELU)[63] is used as the activation function for the hidden layers.

The encoder consists of three layers (128-64-32) and the decoder consists of two layers (64–128). The weights of the layer that outputs the parameters of the cell-cycle component are set with Xavier normal initialization,[64] while the remaining weights are set with the default initialization. A curvature of -2 is used for the cell-cycle component. The dimensionality of the cell-cycle component is set to two to facilitate visualization and to embed the cycling cells in a circular structure. The model is trained for 500 epochs using mini-batches of size 128 with the AdamW[65] optimization algorithm, a learning rate of 0.001 and a weight decay of 0.01.

The number of epochs during which the gradient is stopped for $\mathbf{z}_i^1$ in the reconstruction of $\mathbf{x}_i^2$ is a hyperparameter. For the mouse pancreas dataset, it was stopped for all 500 epochs due to the presence of a strong differentiation signal, which could be partially captured by the cell-cycle marker genes. For all other datasets, the gradient for $\mathbf{z}_i^1$ was not stopped at any point. By default, CellUntangler does not use stop-gradient for two reasons. First, for our case with only 229 cell-cycle genes, many genes that can also contribute are not in our 229-marker gene list, e.g., Revelio uses a set of 800 cell-cycle marker genes. This could potentially lead to inferior performance, especially in cases without enough marker genes to capture the signal of interest, because the non-cell-cycle marker genes are not informative for capturing the cell-cycle signal by using stop-gradient in our setting. Second, as the number of cell-cycle marker genes is relatively small, it is efficient to learn $\mathbf{z}^1$ and thus after a number of epochs, $\mathbf{z}^1$ changes very little (essentially becomes 'constant') compared to $\mathbf{z}^2$. However, because of the strong cell differentiation signal in the mouse pancreas dataset, the cell-cycle marker genes also partially capture the cell differentiation signal. Without using stop-gradient for $\mathbf{z}^1$, it could potentially lead to $\mathbf{z}^2$ not adequately capturing the cell differentiation signal (as it is partially captured by $\mathbf{z}^1$). By using stop-gradient for $\mathbf{z}^1$, we ensure that $\mathbf{z}^1$ primarily captures the cell-cycle signal (which is still dominant in the cell-cycle marker genes), and that $\mathbf{z}^2$ captures the cell differentiation signal.

CPU experiments were run on a server with 64 GB RAM, eight available AMD EPYC 7B12 CPUs (2.25 GHz and 512 KB L2 cache). GPU experiments were run on an Nvidia A100 GPU with 80 GB RAM.

Cell-cycle genes and other marker genes

An input list of 229 human cell-cycle genes was derived from a single-cell esophageal mucosal cell atlas[47] as follows. For 10 of 11 cycling cell subsets, including cycling fibroblasts, endothelial cells, pericytes, mast cells, macrophages, dendritic cells, B cells, $CD4^+$ T cells, $CD8^+$, and NK cells, differential expression analysis was performed to find genes differentially expressed between cycling cells and their non-cycling counterparts in the same cell type, e.g., cycling B cells compared to non-cycling B cells. Cycling signature genes were defined as genes that were upregulated (Bonferroni adjusted *p* values <0.01, log2 fold-change >0.5, expressed in at most 15% of the non-cycling cell subsets) in at least five of the cycling subsets, to a total of 194 genes. These 194 cycling signature genes were combined with 97 cell-cycle marker genes from an earlier study[49] (62 overlapping genes), resulting in a final list of 229 cell-cycle signature genes. For differential expression analysis, we used the Mann-Whitney *U* test from Seurat.[30]

For mouse datasets, orthologs of the human cell-cycle genes were identified. Orthologs were obtained from the Mouse Genome Informatics (MGI) Resource.[66] Of the 229 cell-cycle signature genes, 16 were not present in the table used. Orthologs were found for 15 of the absent genes by using the search bar of the Mouse Genome Informatics (MGI) Resource. No orthologs were found for *FAM111B*. This resulted in a total of 256 mouse cell-cycle signature genes as some human genes had more than one mouse ortholog.

Similarly, we used a set of 65 marker genes to capture the interferon response signature in analyzing the high-grade serous ovarian cancer dataset.[55] These 65 genes were derived from differential expression analysis of a scRNA-seq dataset comparing control human PBMCs and PBMCs after interferon beta stimulation.[52] For the 13 PBMC cell types identified by Seurat,[30] we performed differential expression analysis (Mann-Whitney *U* test) between stimulated cells and control cells. Interferon response signature genes were defined as those upregulated in stimulated cells compared to control cells (Bonferroni adjusted *p* values <0.01, log2 fold-change >0.5, and expressed in at most 30% of control cell subsets) in at least nine cell types.

When selecting highly variable genes in preprocessing, any cell-cycle genes (or other gene signature marker genes used for capturing the signal of interest) that are initially filtered out are added back.

Cell-cycle pseudotime

A cell's pseudotime ranges from [0°,360°] or [0,2π]. Pseudotime was obtained by first projecting $\mathbf{z}_i^1 \in \mathbb{H}_K^2$ from Lorentz to Poincaré coordinates using the equation $\frac{\left[z_{i1}^1, z_{i2}^1\right]^T}{1+\sqrt{|K|}z_{i0}^1}$. The pseudotime was measured as the angle of the projected Poincaré coordinates for each cell, relative to the edge from origin of the Poincaré disk, (0,0), to point (1,0).

Cell-cycle reconstruction

To assess the quality of the cell-cycle reconstruction, Pearson's correlation coefficient of the pseudotime obtained from CellUntangler and the ground truth pseudotime was calculated. For the HeLa cell dataset, cell-cycle time was used as the ground-truth pseudotime. As the ground truth pseudotime and computed pseudotime may not be aligned, a rotation was applied, and, if necessary, the direction of the estimated pseudotime was flipped. The pseudotime with the highest Pearson correlation coefficient with the ground truth pseudotime was used to measure CellUntangler's performance and for comparison of different methods. As the cell-cycle angle was available for the HeLa cell dataset, the correlation between the *sin* of the ground truth angle and the *sin* of the measured pseudotime was also calculated. This additional correlation was used because cell-cycle pseudotime is circular, meaning that values near 0 and 2π should be considered similar. Using the Pearson correlation coefficient directly on these values could result in a lower correlation. The use of the *sin* function helps to address this issue by mapping 0 and 2π to the same value, thereby improving correlation measurement.

Cell identity of cycling cells

After separating the non-cycling cells from the cycling cells based on the manually curated cell type,[37] *k*-NN was used to determine the identity of the cycling cells. The identity of a cell was labeled as uncertain if any one of the classifiers for different values of *k* did not predict the same cell type. We used values of 5, 11, 17, 23, 29, 35, 41, 47, 53, 59, 65, 71, and 73 for *k*.

Batch effect removal

To address batch effects in the datasets, CellUntangler takes as input, the batch or batches. We did not use any batches for the HeLa dataset, mouse embryonic stem cells dataset, and mouse pancreas dataset. For the immune cells dataset, we used chemistry (Chemistry) and donor (Donor) as the batches. For the mouse retinal dataset, we used time as the batch. We used replicate (rep) as the batch for the mouse liver dataset. For the liver hepatocytes from mice after feeding, we used time as the batch only to $\mathbf{z}^1$, but not to $\mathbf{z}^2$. For the eosinophilic esophagitis dataset, we used 10x Genomics chemistry version (v2, v3, or dual-index v3) as the batch. To aid in better integration of stimulated and control cells for the peripheral blood mononuclear cells dataset, we used the stimulation status (stim) as the batch effect for only the cell-type embeddings, $\mathbf{z}^1$. Donor ID (donor_id) and tissue were used as the batches for the ovarian cancer dataset.

Discovering upregulated genes

To find upregulated genes in the peripheral blood mononuclear cells stimulated with interferon beta, we first performed PCA on the non-cell-type embeddings, $\mathbf{z}^2$, then selected one hundred stimulated cells and one hundred control cells based on PC1 and PC2. PC1 and PC2 captured the variation between control and stimulated cells. We reconstructed $\mathbf{z}^2$ for each of these cells using four PCs and passed the reconstructed $\mathbf{z}^2$ to CellUntangler's decoder to obtain the reconstructed gene expression. $\mathbf{z}^1$ was set to 0. The one hundred stimulated cells and one hundred control cells were grouped into one hundred pairs, and we subtracted the reconstructed control expression from reconstructed stimulated expression for each pair. We ranked the genes by the largest positive difference for each pair and picked the top 50 genes. Cell-type marker genes in our list were filtered prior to picking the top 50 genes for each pair. We then counted the frequency of the genes in the top 50 genes for each pair.

Similarly, for the mast cells, we first did a PCA analysis of the non-tissue dissociation embeddings, $\mathbf{z}^2$, and found that PC1, PC3, and PC4 captured the disease signal. We thus picked one hundred points from the 'healthy' region based on PC1-4 (we set PC2 to 0) and one hundred points from the 'Active' region based on PC1-4 (we set PC2 to 0). We grouped these two hundred points into one hundred pairs composed of one healthy cell and one active cell and then used the CellUntangler decoder to decode the reconstructed latent coordinates for each pair, and calculated their difference. When decoding, $\mathbf{z}^1$ was set to coordinates with a low tissue dissociation score, (0.005, −2.5). We obtained the top 50 genes with the largest positive difference (active - healthy) for each pair and counted the frequency of these genes.

Parameter setting for other methods

When using the Scanpy preprocessing pipeline, counts were normalized to 10,000 and then log1p-transformed. To obtain the cell-cycle stages for cells of a dataset, Revelio[29] was run with the 800 genes included in the Revelio package, categorized into five groups: G1, G1.S, G2, G2.M, M.G1. Seurat[30–33] for cell-cycle analysis only requires S and G2.M phase genes, and Seurat was run using all S and G2.M genes present in the cell-cycle gene list we used for CellUntangler. Genes were assigned S or G2.M based on the gene list from Revelio. SiFT was run by first normalizing the counts to 10,000 and then log1p-transforming the normalized counts with the 229 gene list defined above and a *k*-NN kernel based on the SiFT tutorial. scPrisma was run using the default parameters in their *de novo*

reconstruction tutorial for cyclic signals. Classical monocytes were filtered out due to memory constraints in running scPrisma. Default parameter settings were used for Harmony.

Datasets

The HeLa dataset consists of 1,477 human cells profiled by Drop-seq[67] and 4,545 genes were used based on the Revelio pipeline.[29]

The mouse embryonic stem cells dataset consists of 5,637 cells and 11,625 genes profiled by 10x Chromium. The cells were filtered for quality by Riba et al.[6] Genes were those present in both the raw and author-processed data.[6]

Human myeloid cells[37] were profiled using a Chromium chip from 10x Genomics, the Single Cell 5′ Reagent (v1 and v2), and the 3′ Reagent (v3) Kit from twelve different donors. The dataset consists of 51,552 cells, and 29,376 cells of them were included in the analysis, selected as dendritic cells, monocytes, cycling cells, and MNP/T doublets. Highly variable genes were selected with Scanpy and any cycling genes not included in the highly variable gene list were added, resulting in 1,903 genes.

Mouse retinal cells[38] were profiled using the 10x Chromium platform. The dataset consists of 121,309 cells. We used highly variable genes selected with Scanpy and added any cycling genes not included, resulting in 2,684 genes.

Mouse pancreatic stem cells were profiled using the 10x Chromium platform. The original dataset consists of 36,361 cells across four different embryonic stages, and the 3,559 cells from the E15.5 embryos selected in Zheng et al.[5] were used, with the highly variable genes in the original study,[39] along with any cell-cycle genes not included for a total of 4,123 genes.

The mouse liver dataset was profiled using the 10x Chromium platform. 18,378 cells were used and 5,058 genes consisting of those remaining after filtering based on the consistency of the replicates,[43] along with any other zonation genes.

The liver hepatocytes[45] from mice in fed, fasted, and starvation states were profiled using the 10x Chromium platform (v3 chemistry). The cells were from five time points: 0, 4, 8, 16, and 24 h after feeding. After filtering non-hepatocytes (macrophages, endothelial cells) and low-quality cells (e.g., those with a low number of detected genes), we retained 18,715 hepatocytes and 3,167 highly variable genes (including the 50 spatial zonation marker genes).

Cells from the esophageal mucosa[47] were profiled using the 10x Chromium platform (v2 and v3 chemistry), yielding a total of 421,312 individual cells. We restricted our analysis to mast cells, resulting in 27,248 cells. All 33,694 genes from the dataset were included in the analysis.

Peripheral blood mononuclear cells were divided into a control group and a stimulated group, with the stimulated group cells treated with interferon beta.[52] The 10x Chromium platform was used for single-cell library preparation. The dataset consists of 13,999 cells and 14,053 genes, all of which were included in analysis.

The ovarian cancer dataset[55] was profiled using the 10x Chromium platform (v3 chemistry). All 927,205 high-quality cells from the study were used for analysis. Genes that were not filtered out in the original study were used, as well as any interferon beta marker genes, resulting in 3,038 genes.

Cell Genomics, Volume *6*

Supplemental information

CellUntangler: Separating distinct biological signals in single-cell data with deep generative models

Sarah Chen, Aviv Regev, Anne Condon, and Jiarui Ding

Supplementary Information

Supplemental Figures

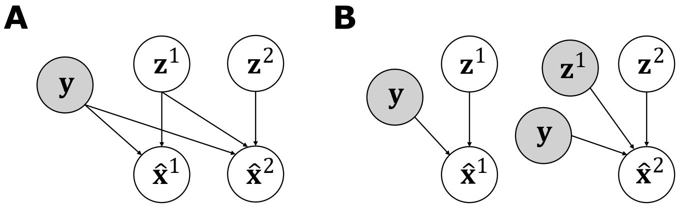

Figure S1: Probabilistic graphical models for CellUntangler, related to Figure 1. The probabilistic graphical model for CellUntangler when the latent representation is decomposed into $\mathbf{z}^1$ and $\mathbf{z}^2$, (**A**) when stop gradient is not used, and (**B**) when stop gradient is used.

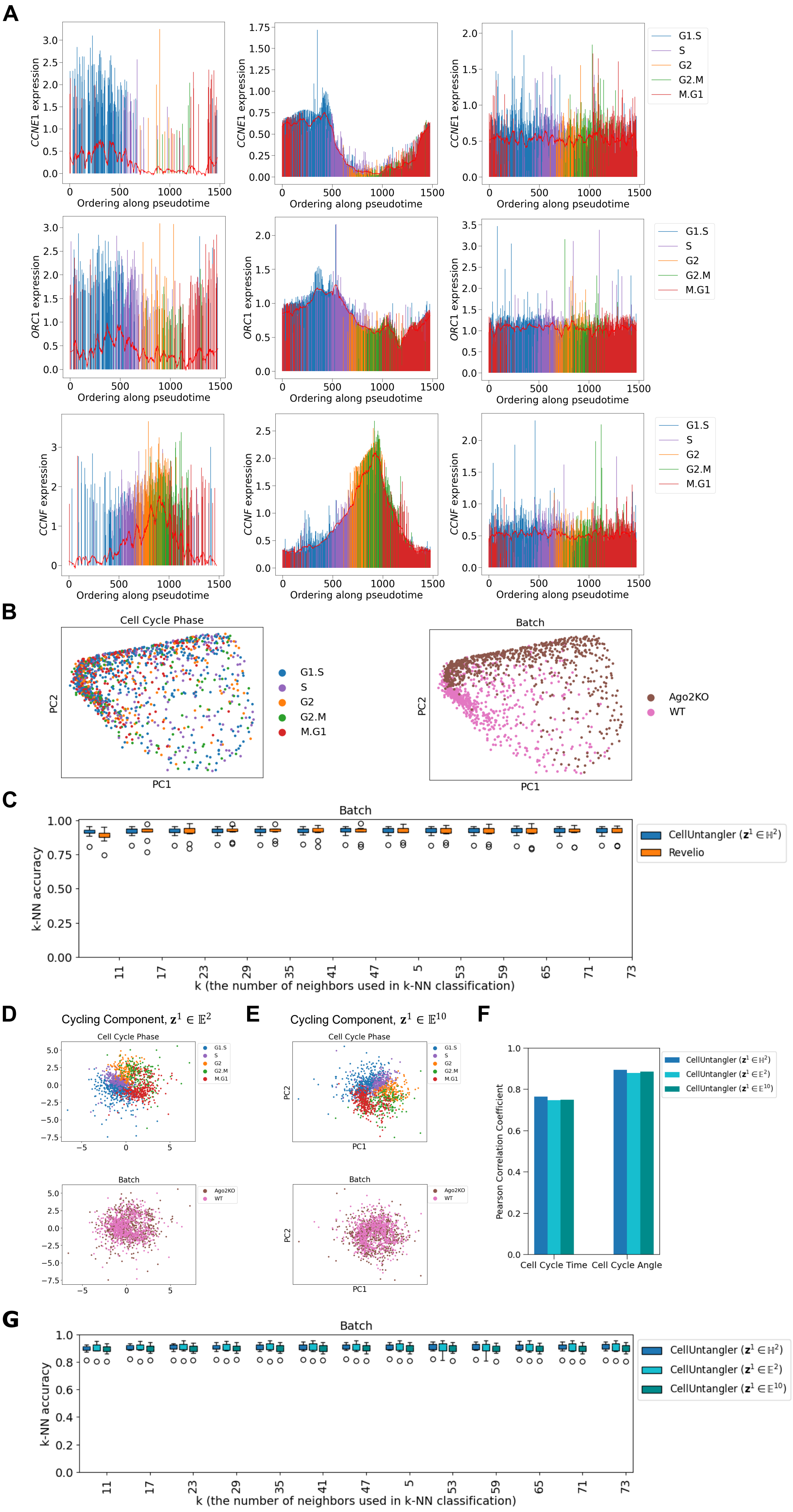

A
CCNE1 expression
ORC1 expression
CCNF expression
Ordering along pseudotime
G1.S
S
G2
G2.M
M.G1
B
Cell Cycle Phase
Batch
PC1
PC2
Ago2KO
WT
C
Batch
k-NN accuracy
k (the number of neighbors used in k-NN classification)
CellUntangler ($\mathbf{z}^1 \in \mathbb{H}^2$)
Revelio
D
Cycling Component, $\mathbf{z}^1 \in \mathbb{E}^2$
E
Cycling Component, $\mathbf{z}^1 \in \mathbb{E}^{10}$
F
Pearson Correlation Coefficient
Cell Cycle Time
Cell Cycle Angle
CellUntangler ($\mathbf{z}^1 \in \mathbb{H}^2$)
CellUntangler ($\mathbf{z}^1 \in \mathbb{E}^2$)
CellUntangler ($\mathbf{z}^1 \in \mathbb{E}^{10}$)
G
Batch
k-NN accuracy
k (the number of neighbors used in k-NN classification)

Figure S2: CellUntangler captures and removes the cell cycle from HeLa cells, related to Figure 2. (**A**) Gene expression of *CCNE1* (G1.S), *ORC1* (G1.S), and *CCNF* (G2) as a function of pseudotime obtained with the embeddings from CellUntangler using the original preprocessed data (size-factor normalized and log1p-transformed, left) and the reconstructed gene expression output from CellUntangler after decoding $\mathbf{z}^1$ and $\mathbf{z}^2$ (right). Cell cycle stages (G1.S, G2) are in parentheses. (**B**) PCA representation of the reconstructed data obtained by running $\mathbf{z}^2$ through CellUntangler's decoder. (**C**) Accuracy of k-nearest neighbors on wild type and knockout cells using $\mathbf{z}^2$ from CellUntangler and PCA embeddings from Revelio, when using the Revelio genes for CellUntangler and Revelio. Boxplots depict the medians and the interquartile ranges (IQR). The whiskers are the lowest datum still within 1.5IQR of the lower quartile and the highest datum still within 1.5IQR of the upper quartile. Individual points below and above the whiskers indicate outliers. (**D**) The first component of CellUntangler when $\mathbf{z}^1 \in \mathbb{E}^2$ and (**E**) when $\mathbf{z}^1 \in \mathbb{E}^{10}$. (**F**) Comparison of cell cycle reconstruction when using the hyperbolic space with the RoWN or the Euclidean space to capture the cell cycle. (**G**) Accuracy of k-nearest neighbors on wild type and knockout cells using $\mathbf{z}^2$ from CellUntangler and either the hyperbolic space with the RoWN or the Euclidean space to capture the cell cycle. Boxplots depict the medians and the interquartile ranges (IQRs). The whiskers are the lowest datum still within 1.5 IQR of the lower quartile and the highest datum still within 1.5 IQR of the upper quartile. Individual points below and above the whiskers indicate outliers.

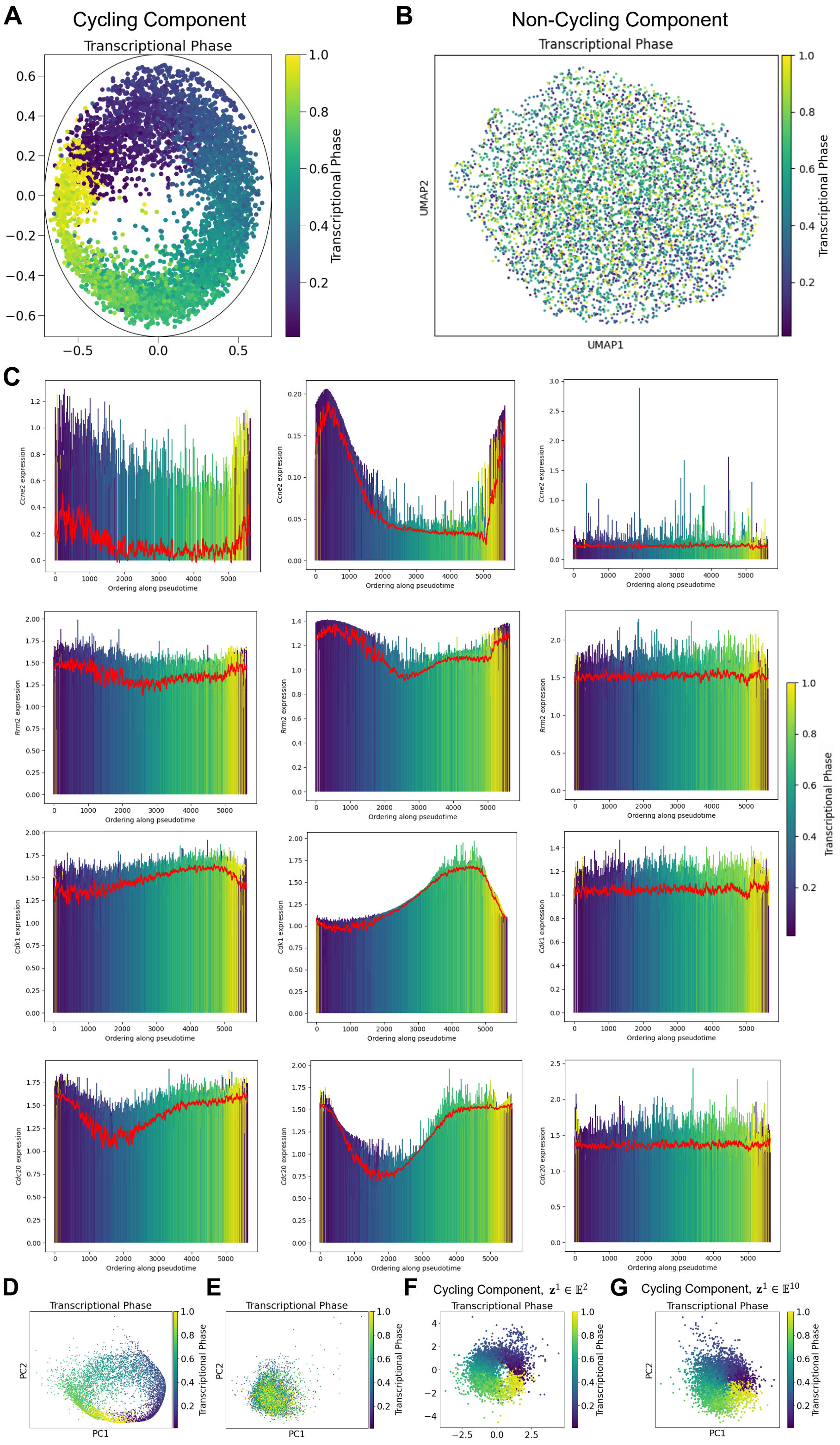

A
Cycling Component
Transcriptional Phase
B
Non-Cycling Component
Transcriptional Phase
UMAP1
UMAP2
C
Ccne2 expression
Rrm2 expression
Cdk1 expression
Cdc20 expression
Ordering along pseudotime
Transcriptional Phase
D
Transcriptional Phase
PC1
PC2
E
Transcriptional Phase
PC1
PC2
F
Cycling Component, $\mathbf{z}^1 \in \mathbb{E}^2$
Transcriptional Phase
G
Cycling Component, $\mathbf{z}^1 \in \mathbb{E}^{10}$
Transcriptional Phase
PC1
PC2

Figure S3: Analysis of the embeddings from CellUntangler on the mESCs demonstrates the capturing and removal of the cell cycle signal, related to Figure 2. (**A**) Visualization of the embeddings for the first component, $\mathbf{z}^1$, where cells were colored by the transcriptional phase output from the original study, and (**B**) embeddings for the second component $\mathbf{z}^2$. (**C**) Marker gene expression across cells when ordered by pseudotime obtained using $\mathbf{z}^1$ and colored using the transcriptional phase from DeepCycle of the original preprocessed data (size-factor normalized and log1p-transformed, left), and the reconstructed gene expression output from CellUntangler after decoding $\mathbf{z}^1$ (middle) and $\mathbf{z}^2$ (right). From top to bottom, *Ccne2* (G1.S), *Rrm2* (S), *Cdk1* (G2), and *Cdc20* (M). Cell cycle stages (G1.S, S, G2, M) are in parentheses. (**D**, **E**) PCA representations of the data reconstructions obtained from $\mathbf{z}^1$ (**A**) and $\mathbf{z}^2$ (**B**), respectively. (**F**) The first component of CellUntangler when $\mathbf{z}^1 \in \mathbb{E}^2$ and (**G**) when $\mathbf{z}^1 \in \mathbb{E}^{10}$.

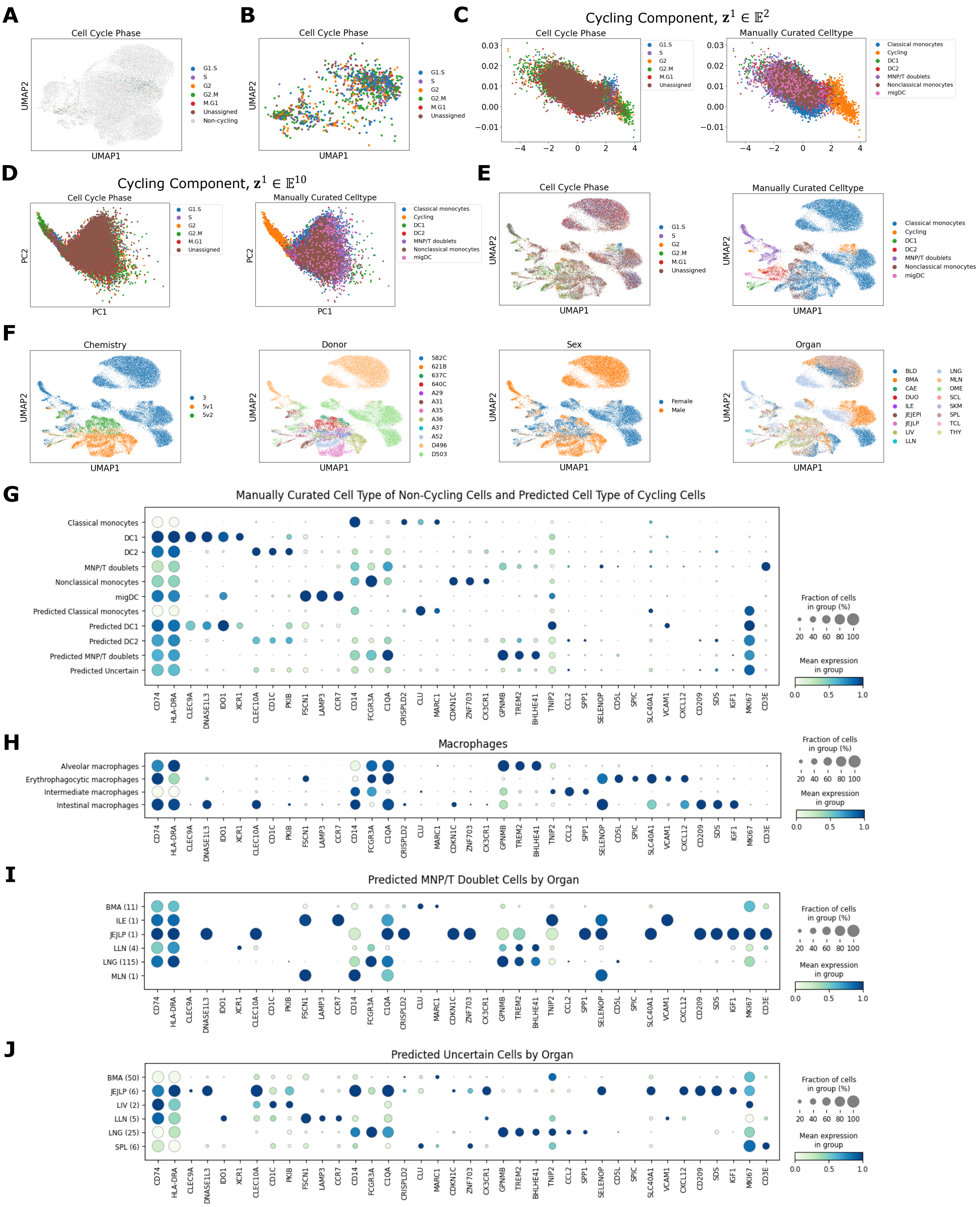
A
Cell Cycle Phase
UMAP1
UMAP2
G1.S
S
G2
G2.M
M.G1
Unassigned
Non-cycling
B
Cell Cycle Phase
C
Cycling Component, $\mathbf{z}^1 \in \mathbb{E}^2$
Manually Curated Celltype
Classical monocytes
Cycling
DC1
DC2
MNP/T doublets
Nonclassical monocytes
migDC
D
Cycling Component, $\mathbf{z}^1 \in \mathbb{E}^{10}$
PC1
PC2
E
F
Chemistry
3
5v1
5v2
Donor
582C
621B
637C
640C
A29
A31
A35
A36
A37
A52
D496
D503
Sex
Female
Male
Organ
BLD
BMA
CAE
DUO
ILE
JEJEPI
JEJLP
LIV
LLN
LNG
MLN
OME
SCL
SKM
SPL
TCL
THY
G
Manually Curated Cell Type of Non-Cycling Cells and Predicted Cell Type of Cycling Cells
Predicted Classical monocytes
Predicted DC1
Predicted DC2
Predicted MNP/T doublets
Predicted Uncertain
CD74
HLA-DRA
CLEC9A
DNASE1L3
IDO1
XCR1
CLEC10A
CD1C
PKIB
FSCN1
LAMP3
CCR7
CD14
FCGR3A
C1QA
CRISPLD2
CLU
MARC1
CDKN1C
ZNF703
CX3CR1
GPNMB
TREM2
BHLHE41
TNIP2
CCL2
SPP1
SELENOP
CD5L
SPIC
SLC40A1
VCAM1
CXCL12
CD209
SDS
IGF1
MKI67
CD3E
Fraction of cells in group (%)
Mean expression in group
H
Macrophages
Alveolar macrophages
Erythrophagocytic macrophages
Intermediate macrophages
Intestinal macrophages
I
Predicted MNP/T Doublet Cells by Organ
BMA (11)
ILE (1)
JEJLP (1)
LLN (4)
LNG (115)
MLN (1)
J
Predicted Uncertain Cells by Organ
BMA (50)
JEJLP (6)
LIV (2)
LLN (5)
LNG (25)
SPL (6)

Figure S4: Applying CellUntangler and Scanpy on the immune cells dataset to remove the effects of the cell cycle, related to Figure 3. (**A**) The second component, $\mathbf{z}^2$, of CellUntangler projected to 2D using UMAP when only cycling cells are colored by cell cycle phase while non-cycling cells are in gray. (**B**) The same UMAP as (**A**) but only the cycling cells. (**C**) The first component of CellUntangler when $\mathbf{z}^1 \in \mathbb{E}^2$ and (**D**) when $\mathbf{z}^1 \in \mathbb{E}^{10}$, colored by cell cycle phase (left) and manually curated cell type (right). (**E**) UMAP visualizations colored by cell cycle phase (left) and manually curated cell type (right) after regressing out the cell cycle using Scanpy and performing PCA. (**F**) UMAP visualizations colored by different batches present in the dataset, after regressing out the cell cycle using Scanpy and performing PCA. (**G**) Marker gene expression across cell type of the non-cycling cells and across predicted cell type of the cycling cells. The cell type was considered uncertain if one or more of the *k*-NN classifiers predicted a different cell type from the rest. (**H**) Marker gene expression of the macrophages present in the original dataset of immune cells. (**I**) Marker gene expression of cycling cells predicted to be MNP/T doublets and (**J**) cycling cells whose identity was uncertain. The organs are bone marrow (BMA), ileum (ILE), the lamina propria of the jejunum (JEJLP), lung-draining lymph nodes (LLN), lung (LG), mesenteric lymph nodes (MLN), liver (LIV), and spleen (SPL). The number of cells from each organ is indicated in the parentheses for (**J**) and (**I**).

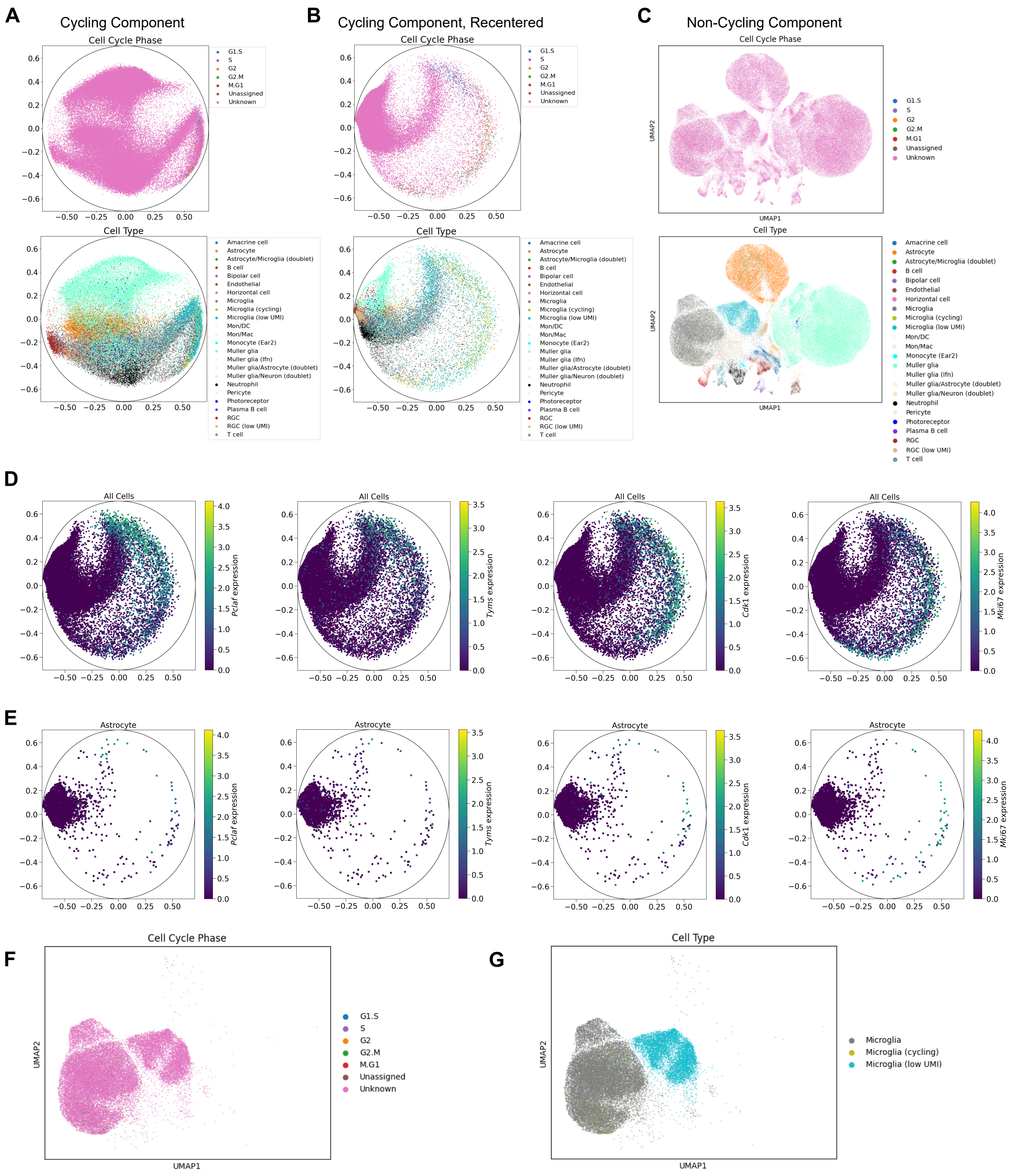
A
Cycling Component
Cell Cycle Phase
G1.S
S
G2
G2.M
M.G1
Unassigned
Unknown
Cell Type
B
Cycling Component, Recentered
C
Non-Cycling Component
UMAP1
UMAP2
Amacrine cell
Astrocyte
Astrocyte/Microglia (doublet)
B cell
Bipolar cell
Endothelial
Horizontal cell
Microglia
Microglia (cycling)
Microglia (low UMI)
Mon/DC
Mon/Mac
Monocyte (Ear2)
Muller glia
Muller glia (Ifn)
Muller glia/Astrocyte (doublet)
Muller glia/Neuron (doublet)
Neutrophil
Pericyte
Photoreceptor
Plasma B cell
RGC
RGC (low UMI)
T cell
D
All Cells
Pclaf expression
Tyms expression
Cdk1 expression
Mki67 expression
E
Astrocyte
F
G

Figure S5: Capturing and removing the cell cycle signal in the mouse retinal dataset, related to Figure 3. (**A**) The embeddings, $\mathbf{z}^1$, before recentering and (**B**) after recentering the origin to (-0.5, 0.1). (**C**) The embeddings of the second component, $\mathbf{z}^2$, after projecting to 2D using UMAP. (**D**) The embeddings of the first component, $\mathbf{z}^1$, for all cells and (**E**) only astrocytes colored by *Pclaf* (G1.S), *Tyms* (S), *Cdk1* (G2), and *Mki67* (M) gene expression. Cell cycle stages are in parentheses. (**F**) The projected UMAP with only the microglia colored by cell cycle phase and (**G**) cell state.

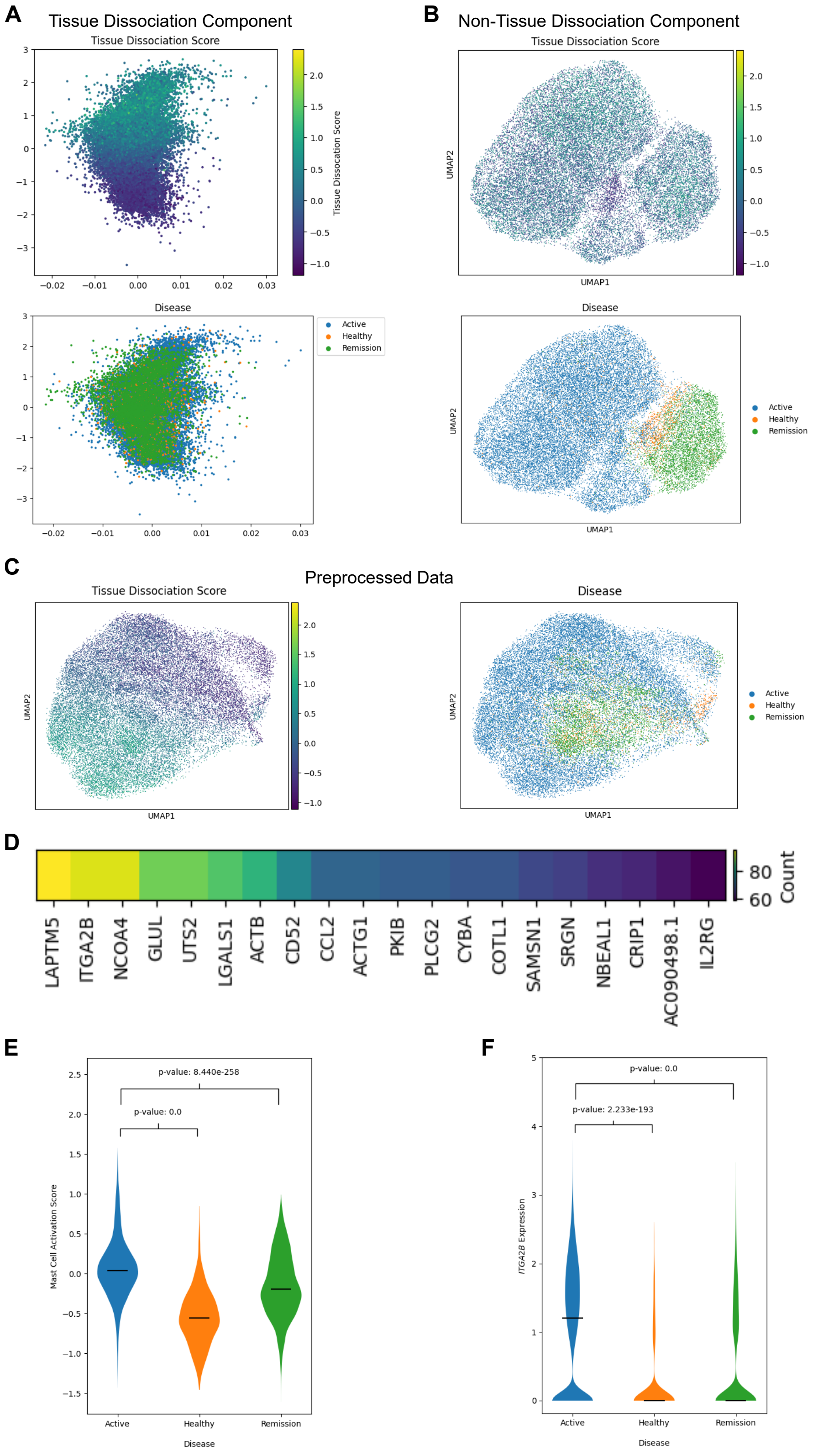

A
Tissue Dissociation Component
Tissue Dissociation Score
Tissue Dissocation Score
Disease
Active
Healthy
Remission
B
Non-Tissue Dissociation Component
Tissue Dissociation Score
UMAP1
UMAP2
Disease
C
Preprocessed Data
Tissue Dissociation Score
Disease
D
LAPTM5
ITGA2B
NCOA4
GLUL
UTS2
LGALS1
ACTB
CD52
CCL2
ACTG1
PKIB
PLCG2
CYBA
COTL1
SAMSN1
SRGN
NBEAL1
CRIP1
AC090498.1
IL2RG
Count
E
p-value: 8.440e-258
p-value: 0.0
Mast Cell Activation Score
Active
Healthy
Remission
Disease
F
p-value: 0.0
p-value: 2.233e-193
ITGA2B Expression
Active
Healthy
Remission
Disease

Figure S6: CellUntangler filters the tissue dissociation signal from mast cells, related to Figure 7. (**A**) The embeddings of the first component, $\mathbf{z}^1$, and (**B**) the embeddings of the second component, $\mathbf{z}^2$, after projecting to 2D using UMAP. (**C**) The standard preprocessing pipeline outputs, projected to 2D using UMAP. Batch correction on 10x Genomics chemistry version is performed using Harmony. (**D**) The top 20 genes found that were upregulated in active EoE patients compared to healthy individuals. (**E**) The signature scores computed using the 20 genes from (**D**) and the original preprocessed data (size-factor normalized and log1p-transformed). (**F**) Expression of *ITGA2B* in the original preprocessed data (size-factor normalized and log1p-transformed). Statistical tests were performed using a two-sided Mann-Whitney *U* test between disease statuses (Active and Healthy as well as Active and Remission). Horizontal lines indicate the medians.

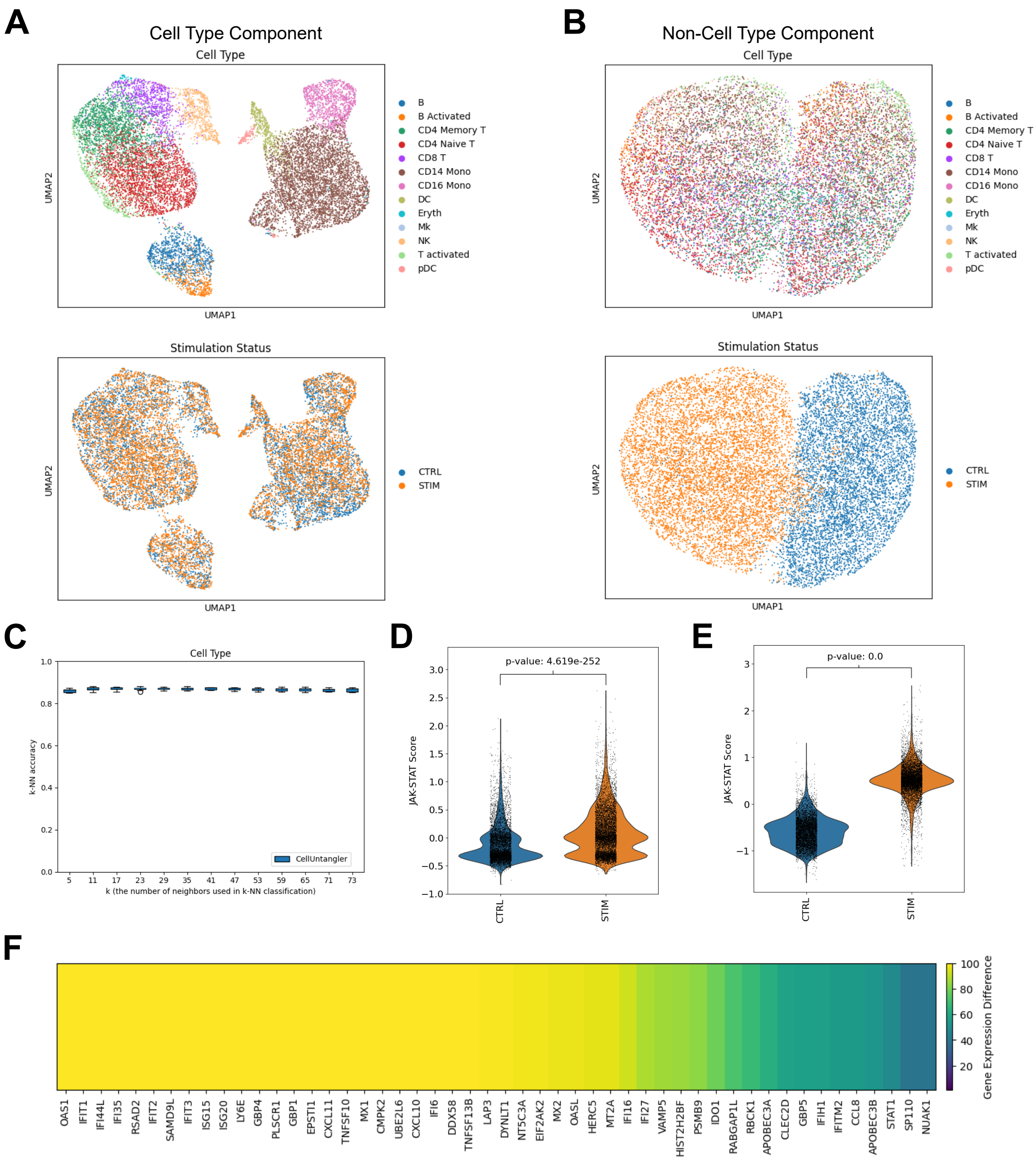

Figure S7: CellUntangler filters cell type to isolate control and stimulation signals, related to Figure 7. (**A**) The embeddings of the first component, $\mathbf{z}^1$, and (**B**) the embeddings of the second component, $\mathbf{z}^2$, both projected to 2D using UMAP. (**C**) The *k*-NN accuracies on cell type using $\mathbf{z}^1$ and 10-fold cross-validation. (**D**) The JAK-STAT scores using the original pre-processed data (size-factor normalized and log1p-transformed), and (**E**) the JAK-STAT scores using the reconstructed gene expression from CellUntangler after decoding $\mathbf{z}^2$. Statistical tests were performed with a two-sided Mann-Whitney *U* test between the control (CTRL) and stimulated (STIM) cells. (**F**) The top 50 upregulated genes in stimulated cells relative to control cells found using the reconstructed gene expression output from CellUntangler after decoding $\mathbf{z}^2$.

Supplemental Tables

	CellUntangler	tricycle	scPrisma	Revelio	Seurat	SiFT	DeepCycle	reCAT	Cyclum	ccRemover	SC1CC
Cell cycle pseudotime or ordering	Yes	Yes	Yes	Yes	No^{a}	No	Yes	Yes	Yes	No	Yes
Filter cell cycle	Yes	No	Yes	Yes	Yes	Yes	No	No	Yes	Yes	No
Applicable to non-cyclic signals	Yes	No	Yes	No	Yes	Yes	No	No	No	No	No

Table S2: Scope of application for CellUntangler and other methods, related to Figure 2. We compared the cell cycle pseudotime and cell cycle filtering of CellUntangler with representative and well-maintained models (tricycle, scPrisma, Revelio, Seurat, and SiFT). DeepCycle was not used in our experiments due to the requirement of spliced and unspliced counts. ^{a}Although Seurat does not provide a continuous cell cycle pseudotime or ordering, it does provide gene signature scores and assign discrete cell cycle phases. scPrisma does not perform well in cases where only a subset of cells are cycling (Figure **??**F).

	CellUntangler			Revelio	scPrisma	SiFT	Seurat	Baseline 1	Baseline 2
	$\mathbf{z}^1 \in \mathbb{H}^2$	$\mathbf{z}^1 \in \mathbb{E}^2$	$\mathbf{z}^1 \in \mathbb{E}^{10}$						
iLISI	0.592	0.611	0.618	**0.654**	0.551	0.646	0.649	0.442	0.457
ARI Leiden (15)	0.472	0.446	0.399	**0.515**	0.280	0.167	0.347	0.148	0.152
NMI Leiden (15)	**0.406**	0.387	0.346	0.400	0.262	0.231	0.334	0.196	0.206
ARI Louvain (15)	**0.468**	0.435	0.348	0.299	0.228	0.142	0.308	0.166	0.167
NMI Louvain (15)	**0.395**	0.364	0.303	0.310	0.217	0.181	0.339	0.162	0.237
ARI Leiden (50)	**0.562**	0.548	0.495	0.313	0.272	0.193	0.281	0.117	0.166
NMI Leiden (50)	**0.467**	0.462	0.412	0.275	0.267	0.227	0.320	0.147	0.223
ARI Louvain (50)	0.489	**0.529**	0.505	0.231	0.271	0.171	0.259	0.080	0.147
NMI Louvain (50)	0.420	**0.442**	0.424	0.275	0.267	0.206	0.319	0.060	0.159
cLISI	**0.834**	0.813	0.812	0.404	0.321	0.270	0.355	0.370	0.452

Table S3: Benchmarking of cell cycle signal removal and revealing of cell identity as a knockout (Ago2KO) cell or wild type (WT) cell on the HeLa data, related to Figure 2. iLISI is reported with respect to cell cycle phase where values closer to 1 indicate better cell cycle removal. Both Leiden and Louvain clustering were used. The number in brackets indicates the number of neighbors used. For the rest of the metrics, the default values were used. The average over ten runs for each of these metrics is reported for CellUntangler. Baseline 1 removes the cell cycle genes in our list and Baseline 2 removes the Revelio genes. iLISI: integration local inverse Simpson's index; ARI: adjusted Rand index; NMI: normalized mutual information; cLISI: normalized cell-type local inverse Simpson's index. The higher the better for these metrics.

	CellUntangler			tricycle	scPrisma	Revelio
	$\mathbf{z}^1 \in \mathbb{H}^2$	$\mathbf{z}^1 \in \mathbb{E}^2$	$\mathbf{z}^1 \in \mathbb{E}^{10}$			
Cell cycle correlation	0.805	**0.833**	0.807	0.516	0.590	0.584

Table S4: Comparison of cell cycle reconstruction for the mESCs data, related to Figure S2. The transcriptional phase output by DeepCycle[1], as analyzed by the original authors of this dataset, is used as the ground truth. The average correlation over ten runs is reported for CellUntangler.

References

1. Riba, A., Oravecz, A., Durik, M., Jiménez, S., Alunni, V., Cerciat, M., Jung, M., Keime, C., Keyes, W. M., and Molina, N. (2022). Cell cycle gene regulation dynamics revealed by RNA velocity and deep-learning. Nature Communications *13*, 2865. 10.1038/s41467-022-30545-8.
