# Supplementary material for: CellUntangler: Separating distinct biological signals in single-cell data with deep generative models
Source: Cell Genom. 2025 Dec 1;6(2):101073. doi: 10.1016/j.xgen.2025.101073 (PMC12903416; doi:10.1016/j.xgen.2025.101073)
Supplement: Document S2. Transparent peer-review records for Chen et al. [file mmc3.pdf]

Cell Genomics

Transparent Peer Review Record

CellUntangler: separating distinct biological signals in single-cell data with deep generative models

Author list: Sarah Chen, Aviv Regev, Anne Condon, and Jiarui Ding

Summary

Initial submission:	Received : January 23rd 2025
	Scientific editor: Judith Nicholson
First round of review:	Number of reviewers: 2<br>Revision invited : August 11th 2025 (after rebuttal)<br>Revision received : August 12th 2025
Second round of review:	Number of reviewers: 2<br>Accepted : 28th October 2025
Data freely available:	Yes
Code freely available:	Yes

This transparent peer review record is not systematically proofread, type-set, or edited. Special characters, formatting, and equations may fail to render properly. Standard procedural text within the editor's letters has been deleted for the sake of brevity, but all official correspondence specific to the manuscript has been preserved.

Referees' reports, first round of review

Reviewer 1:

The paper demonstrates a variant of the VAE framework for signal decomposition with single cell data. The structure of the VAE is setup to perform a signal decomposition into a pre-defined marker gene latent component and a residual component comprising the rest of the input signal. Independence of the two latent components is promoted through the use of the stop gradient trick to prevent an interaction between one part of the latent space and a subset of input features. Further, the dimensions of the latent space are further decomposed into different governing curvatures so that these can be adapted to expected behaviours.

Unusually for a paper to this type of journals, methods were technically well-described and meticulously detailed that I do not find any significant fault or issue with the derivations.

I have the following questions:

Fig 1B: Which data set is this? What does the UMAP show on the original data without cell cycle removal? Could the same effect be achieved by removing the cell cycle genes from the features?

Fig 2D: What is performance of CellUntangler on the same set of genes as Revelio?

Fig 6A: Could the authors clarify the visual interpretation of the zonation plots? The layering discussed in the corresponding section of the main text does not appear to be consistent.

pg 13. "For the mouse pancreas dataset, it was stopped for all 500 epochs due to the presence of a strong differentiation signal. For all other datasets, the gradient for z1i was not stopped at any point." Could the author comment on why the gradient was not stopped?

Overall, the paper is well presented and methodology described clearly and experiments demonstrate the utility of the model. The main limitation is that the paper is largely focused on cell cycle programs with the only exception the last example on zonation where I struggled to see, as clearly, the patterns discussed in the text. While cell cycle correction is important, it is not clear to what extent the implementation is geared toward cell cycle and further alternate examples would be appreciated.

Reviewer 2:

The article introduces CellUntangler, a tool based on deep generative models. CellUntangler is innovative in its multi-space modeling using both hyperbolic and Euclidean spaces, which allows for more precise separation and analysis of different biological signals. Additionally, the authors demonstrate the tool's broad applicability through validation on multiple datasets using various methods, such as KNN classification and validation of marker gene expression. This makes CellUntangler a potentially important tool in the field of single-cell data analysis. However, there are still some areas for improvement:

（1）Although the article mentions the limitations of some other tools, a more comprehensive comparative analysis， including performance metrics and scope of application， could further highlight the advantages of CellUntangler.

（2）The article does not provide a detailed discussion on the computational efficiency and scalability of CellUntangler, especially on large-scale datasets. This is crucial for the practical application of the tool, particularly when dealing with high-throughput single-cell data.

Authors' response to the first round of review

Response to Reviewers

We thank the reviewers for their time and thoughtful comments on our manuscript. We are grateful for the specific comments raised, which are addressed in detail. We first highlight the key revisions and then address each comment in a point-by-point manner below.

The key highlights in our revision are:

1. Additional computational experiments to demonstrate CellUntangler's extensibility to diverse signals. Specifically, we apply CellUntangler to capture the **interferon response signal** in cells from the high-grade serous ovarian cancer tumor microenvironment, aiming to better understand which cell types respond to interferons[1]. Furthermore, we use CellUntangler to disentangle **cell type signal** from interferon stimulation using a human peripheral blood mononuclear cell (PBMC) dataset[2]. We also use CellUntangler to capture and remove the **cell dissociation signal** in mast cells from active eosinophilic esophagitis (EoE) patients, remission EoE patients, and healthy individuals, enabling the identification of genes upregulated in mast cells from active EoE patients[3]. The results from these three datasets are presented in **Figure 7**, **Figure S7**, and **Figure S6** of the updated manuscript.

2. Additional experimental results and figures to help interpret the spatial and temporal signals captured and removed by CellUntangler. We add two UMAPs to demonstrate that the spatial and temporal signals are entangled in liver hepatocytes. We also plot cells from different spatial layers separately and annotate their median coordinates in the embedded space to better illustrate the capture of spatial information. Additionally, we plot the results for a new rhythmic-zonated gene, *Pck1* (instead of the original gene *Hlf*, which is a rhythmic gene), whose expression changes over time and across spatial layers. The results are presented in **Figure 5** of the updated manuscript. More importantly, we analyze **a recently published dataset** profiling mouse liver hepatocytes in fed, fasted, and starvation states[4] and present the results in **Figure 6** of the updated manuscript. In both studies, we show that CellUntangler successfully captures the spatial zonation signal and the coordinates along a latent dimension can be interpreted as a **"pseudospace"**, enabling new biological discoveries and counterfactual reasoning.

3. Additional benchmarking metrics on the HeLa and mouse embryonic stem cell (mESC) datasets. For the HeLa dataset, we include additional metrics to compare the performance of CellUntangler, Seurat, SiFT, scPrisma, and Revelio in removing the effects of the cell cycle and in revealing the identity of cells as knockout (Ago2KO) or wild type (WT), as presented in **Table S3**. For the mESCs dataset, we measure and compare how well the cell cycle is captured by CellUntangler, tricycle, scPrisma, and Revelio, as presented in **Table S4**. We also add a table to summarize the scope of each method and their performance on the two benchmarking datasets, as presented in **Table S2**.

4. Computational efficiency and scalability studies using an atlas of 121,309 retina cells[5]

and a large dataset of high-grade serous ovarian cancer with approximately **one million cells**[1]. We ran CellUntangler on these datasets to generate embeddings in which the cell cycle (or other signals) is captured, and embeddings in which non-cell cycle signals are captured. We subsample an increasingly large number of cells from the ovarian cancer dataset to study how CellUntangler's runtime scales with the number of cells. The results on the two datasets are presented in **Figure S5** and **Figure 7**, respectively.

Response to Reviewer 1

The paper demonstrates a variant of the VAE framework for signal decomposition with single cell data. The structure of the VAE is setup to perform a signal decomposition into a pre-defined marker gene latent component and a residual component comprising the rest of the input signal. Independence of the two latent components is promoted through the use of the stop gradient trick to prevent an interaction between one part of the latent space and a subset of input features. Further, the dimensions of the latent space are further decomposed into different governing curvatures so that these can be adapted to expected behaviours.

Unusually for a paper to this type of journals, methods were technically well-described and meticulously detailed that I do not find any significant fault or issue with the derivations.

We thank the Reviewer for the excellent summary of our study and for the assessment that our work is "technically well-described and meticulously detailed".

I have the following questions:

1. Fig 1B: Which data set is this? What does the UMAP show on the original data without cell cycle removal? Could the same effect be achieved by removing the cell cycle genes from the features?

We thank the reviewer for this question and we regret our omission regarding the UMAP. We did not use a dataset to create this UMAP; rather, it was manually generated to illustrate how CellUntangler might disentangle two biological signals into different latent embeddings. We added this clarification to the **Figure 1** legend.

Removing the cell cycle marker genes does not fully eliminate the cell cycle signal, because the markers we used account for only a proportion of the total cell cycle genes, as noted in our response to the first comment from Reviewer 2 (**Table R2**, the relatively poor performance of Baseline 1 and Baseline 2).

2. Fig 2D: What is performance of CellUntangler on the same set of genes as Revelio?

We thank the reviewer for this question. The below boxplots show the performance of

CellUntangler when using the same 800 cell cycle genes as used for Revelio. We find that CellUntangler tends to perform similarly to or better than Revelio. We added these new results as **Figure S1C**.

By default, CellUntangler uses a set of **229** cell-cycle marker genes and performs slightly worse than Revelio using the **800** marker genes (**Figure 2G**) on the HeLa dataset. These results suggest that adding more cell cycle-specific marker genes could improve performance.

3. Fig 6A: Could the authors clarify the visual interpretation of the zonation plots? The layering discussed in the corresponding section of the main text does not appear to be consistent.

We appreciate the reviewer for asking this question, giving us the opportunity to revise the figure. We regret our lack of clarity regarding the visual interpretation of the zonation plots. First, the "ground-truth" spatial layer information was **computationally inferred** by the original authors[6] and, as expected, since hepatocytes are distributed continuously along the central-portal axis, partitioning hepatocytes into eight layers could introduce ambiguity. Indeed, researchers commonly partition them into three zones (zone 1-3), instead of eight layers[7]. Accordingly, in CellUntangler results, the cells do not completely separate into distinct clusters based off of the zonation layer and somewhat overlap with one another in the original **Figure 6A**. However, the organization follows the expected progression from layer 0 to layer 7, based on the y-coordinates. Layer 0 cells are placed near the bottom of the plot, layer 1 cells are placed slightly on top, and so on, all the way to layer 7 cells. The cells from different times (zeitgeber time, 0h, 6h, 12h, 18h) are mixed in this plot. We change our discussion of the layering in the main text to be more consistent with the plots. Specifically, we update CellUntangler results, plotting the cells from each layer separately, and also add the median position along the y-axis (which captures the spatial zonation signal) of $\mathbf{z}^1$, so that we can clearly see the monotonic increase in the y-coordinates from layer zero through layer seven. We also change layer 0 through layer 7 to be layer 1 through layer 8 to be consistent with previous publications[6]. Furthermore, we visualize the expression of two landmark zonated genes, *Cyp2f2* and *Cyp2e1*.

In the original **Figure 6B**, we plot $\mathbf{z}^2$, which is supposed to capture the circadian rhythm. We can see the cells are separated by time, but mixed by zonation layer, which suggests that $\mathbf{z}^2$ captures the circadian rhythm but not the zonation layer signal.

For the original **Figure 6C** and **Figure 6D**, we want to show the expression patterns of two independent rhythmic-zonated (layer-independent rhythmic and time-independent zonated) genes. For a good conceptual visualization of the expression of these genes, we adopt the figures from Annunziato, S., & Tchorz, J. S. (2021) below (**Figure R1**).

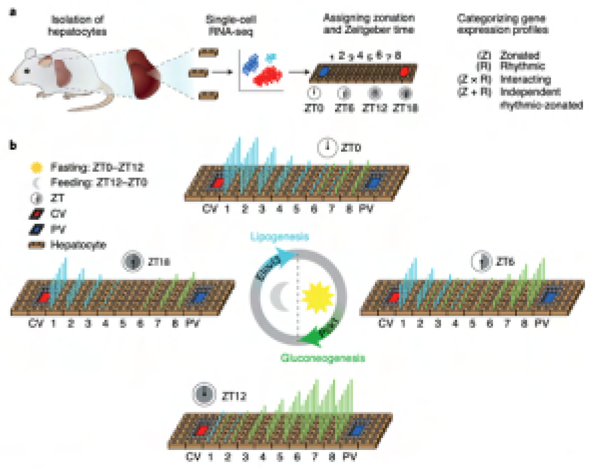

Fig. 1 | **Spatiotemporal distribution of hepatic gene expression. a**, Single-cell RNA-seq of hepatocytes isolated at four different times within a day (Zeitgeber time (ZT) 0, 6, 12 and 18), and assigning each cell to one of the eight porto-central hepatocyte layers, enabled identification of diverse gene expression profiles regulated in space and time. **b**, Exemplary illustration of independent rhythmic-zonated gene expression for *Elovl3* (involved in lipogenesis, specifically elongation of long-chain fatty acids) and *Pck1* (regulating gluconeogenesis) highlights temporal and zonal restrictions of key metabolic processes. Whereas *Elovl3* is expressed mostly during the feeding cycle in pericentral hepatocytes, *Pck1* is predominantly expressed in periportal hepatocytes towards the end of the fasting cycle. CV, central vein; PV, portal vein.

Figure R1: The figure is adopted from Annunziato, S., & Tchorz, J.S. (2021) to show the conceptual expression pattern for two independent rhythmic-zonated genes, *Elovl3* and *Pck1*.

In the original data (**Figure 6C**, blue curve), we can see the variation of *Elovl3* expression across zonation layers (top) and time points (bottom). After filtering the zonation signal (green), *Elovl3* expression is more uniform across the zonation layers (original **Figure 6C**, top) but the expression variation across time is retained (bottom, peaking at time 0, decreasing at time 6, then reaching its lowest point at time 12, and then increasing at time 18). Similarly, after enhancing the zonation signal (orange), we can see a steep decrease in *Elovl3* expression across zonation layers (top), while the expression variation across time is more uniform (bottom).

In the original **Figure 6D**, we used the *Hlf* gene[6], which was classified as a rhythmic gene (expression changes mostly with time). Thus, compared to *Elovl3*, *Hlf* expression showed less variation across the zonation layers (original **Figure 6D**, top). Therefore, it's even harder to see the effects of filtering and enhancing the zonation signals for this gene. In this revision, we replace *Hlf* by *Pck1*, which is another independent rhythmic-zonated gene.

We hope the updated **Figure 6**, which becomes **Figure 5** in the revised manuscript, will be clearer. We replaced the panel **A** with the standard preprocessing pipeline to show entanglement of the spatial and temporal signals. We changed panel **B** to better show the progression of layers. The panels **C** and **D** change to be visualizations of $\mathbf{z}^1$, while the old

panel **B** becomes the new panel **E**. Finally, the original panels **C** and **D** (**Figure 6C, D**) became panels **F** and **G**.

4. pg 13. "For the mouse pancreas dataset, it was stopped for all 500 epochs due to the presence of a strong differentiation signal. For all other datasets, the gradient for z1i was not stopped at any point." Could the author comment on why the gradient was not stopped?

We'd like to thank the reviewer for asking this important question. The primary reason for not applying stop-gradient is that the number of cell cycle marker genes (229) is relatively small, as is the case for other marker sets, such as the 50 zonation marker genes. This makes it efficient to learn $\mathbf{z}^1$ and thus after a number of epochs, $\mathbf{z}^1$ changes very little (essentially becomes 'constant') compared to $\mathbf{z}^2$. In practice, the default setting (without stop-gradient) yields strong performance across all datasets tested in this study.

It is generally challenging to faithfully capture complex cell differentiation trajectories in a two-dimensional space. The mouse pancreas dataset is a complex dataset, with both cycling and non-cycling cells, and there is a strong differentiation signal in these cells. To carefully study the pancreas cell differentiation, some previous studies first removed the ductal cells (mostly in cycling)[8]. Because of the inductive bias of CellUntangler using the hyperbolic space to capture the cell cycle component, CellUntangler generally produces good results ($\mathbf{z}^1$ captures the cell cycle and $\mathbf{z}^2$ captures the cell differentiation) even without using stop-gradient. However, because of the strong cell differentiation signal in this dataset, the cell cycle marker genes also **partially capture this cell differentiation signal** (updated **Figure 4A**). Without using stop-gradient for $\mathbf{z}^1$, it could potentially lead to $\mathbf{z}^1$ to capture more cell differentiation signal, and $\mathbf{z}^2$ to also capture the cell cycle signal. By using stop-gradient for $\mathbf{z}^1$, we make sure that $\mathbf{z}^1$ primarily captures the cell cycle signal (which is still dominant in the cell cycle marker genes), and that $\mathbf{z}^2$ captures the cell differentiation signal. We **update the "Methods" section** to reflect the reasons to use stop-gradient in analyzing this dataset.

To further help address the reviewer's comments, we **compared the results** from running CellUntangler with and without using stop gradient, using the HeLa, mouse pancreas, immune cell, and the mouse liver hepatocyte datasets.

We used CellUntanger to separate the cell cycle signal and non-cell cycle signals into different embeddings, $\mathbf{z}^1$ and $\mathbf{z}^2$ for the HeLa, mouse pancreas, and immune cell datasets. As expected, when visualizing $\mathbf{z}^1$ from different runs of CellUntangler without using stop-gradient, the cells separated by cell cycle stage (**Figure R2A**), while when visualizing $\mathbf{z}^2$, the cells separated by knockout (Ago2KO) and wild type (WT) (**Figure R2B**). Similarly, when stop-gradient is used, in $\mathbf{z}^1$, cells separated by cell cycle stage (**Figure R2C**) and in $\mathbf{z}^2$, cells separated by knockout and wild type (**Figure R2D**). However, when stop-gradient is used, the $\mathbf{z}^1$ embeddings are closer to the origin of the Poincaré disk whereas when stop-gradient is not used, cells lie closer to the edge. The performance for capturing the cell cycle is similar

though (**Figure R2E**). When measuring the k-NN accuracies on knockout and wild type, using stop-gradient results in slightly better performance (**Figure R2F**).

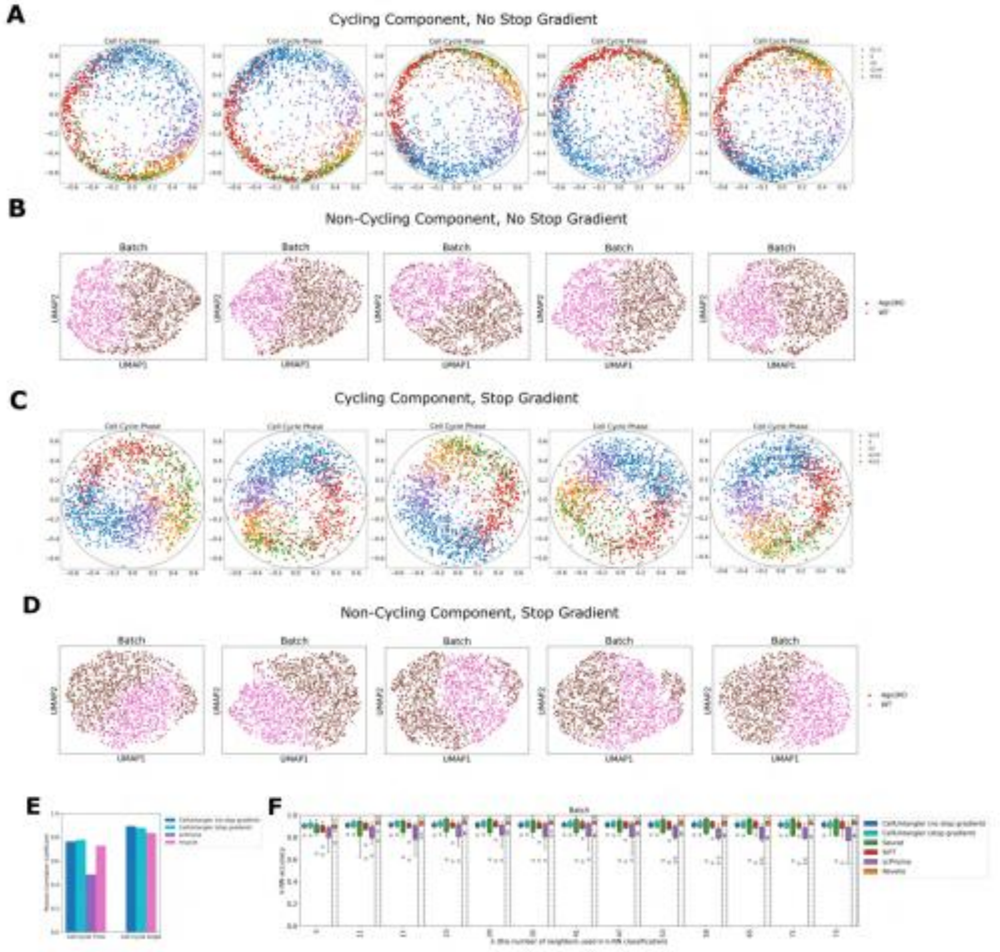

Figure R2: **Using no stop-gradient and stop-gradient on the HeLa dataset** (**A**) The embeddings of the first component, $\mathbf{z}^1$, and (**B**) the embeddings of the second component, $\mathbf{z}^1$ when stop-gradient is not used for five different runs of CellUntangler. (**C**) The embeddings of the first component, $\mathbf{z}^1$, and (**D**) the embeddings of the second component, $\mathbf{z}^2$, when stop-gradient is used for five different runs of CellUntangler. (**E**) Comparison of cell cycle reconstruction. (**F**) Accuracy of k-nearest neighbors on wild type and knockout cells using $\mathbf{z}^2$ from CellUntangler with no stop-gradient and CellUntangler with stop-gradient and different methods. Boxplots depict the medians and the interquartile ranges (IQR) . The whiskers are the lowest datum still within 1.5 IQR of the lower quartile and the highest datum still within 1.5 IQR of the upper quartile. Individual points below and above the whiskers indicate outliers.

For the mouse pancreas dataset, as this dataset has a strong differentiation signal, $\mathbf{z}^1$ partially captures the differentiation trajectory as cycling cells are placed on one side and differentiated cells on the other side when no stop-gradient is used (**Figure R3A**). As some of the differentiation trajectory is captured in $\mathbf{z}^1$, when no stop-gradient is used, the differentiation trajectory may not be captured as clearly in $\mathbf{z}^2$ (**Figure R3B**). By using stop-gradient, while some of the differentiation trajectory is captured in $\mathbf{z}^1$ (**Figure R3C**), we can better capture the differentiation signal in $\mathbf{z}^2$ (**Figure R3D**).

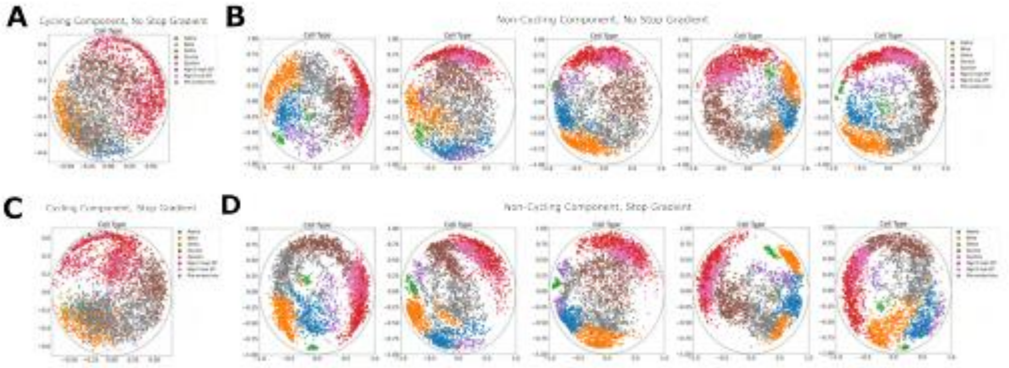

Figure R3: Using no stop-gradient and stop-gradient on the mouse pancreas dataset. (**A**) The embeddings of the first component, $\mathbf{z}^1$, when stop-gradient is not used. (**B**) The embeddings of the second component, $\mathbf{z}^2$ when stop-gradient is not used for five different runs of CellUntangler. (**C**) The embeddings of the first component, $\mathbf{z}^1$, when stop-gradient is used. (**D**) The embeddings of the second, $\mathbf{z}^2$, when stop-gradient is used for five different runs of CellUntangler.

For the immune cells dataset, when using no stop-gradient, $\mathbf{z}^1$ captures the cell cycle signal (**Figure R4A**) and $\mathbf{z}^2$ captures cell type (**Figure R4B**). The results are visually similar when stop-gradient is used (**Figure R4C, D**). Results using the non-cycling cells for both no stop-gradient (**Figure R5A, B**) and stop-gradient are similar as well (**Figure R5C, D**).

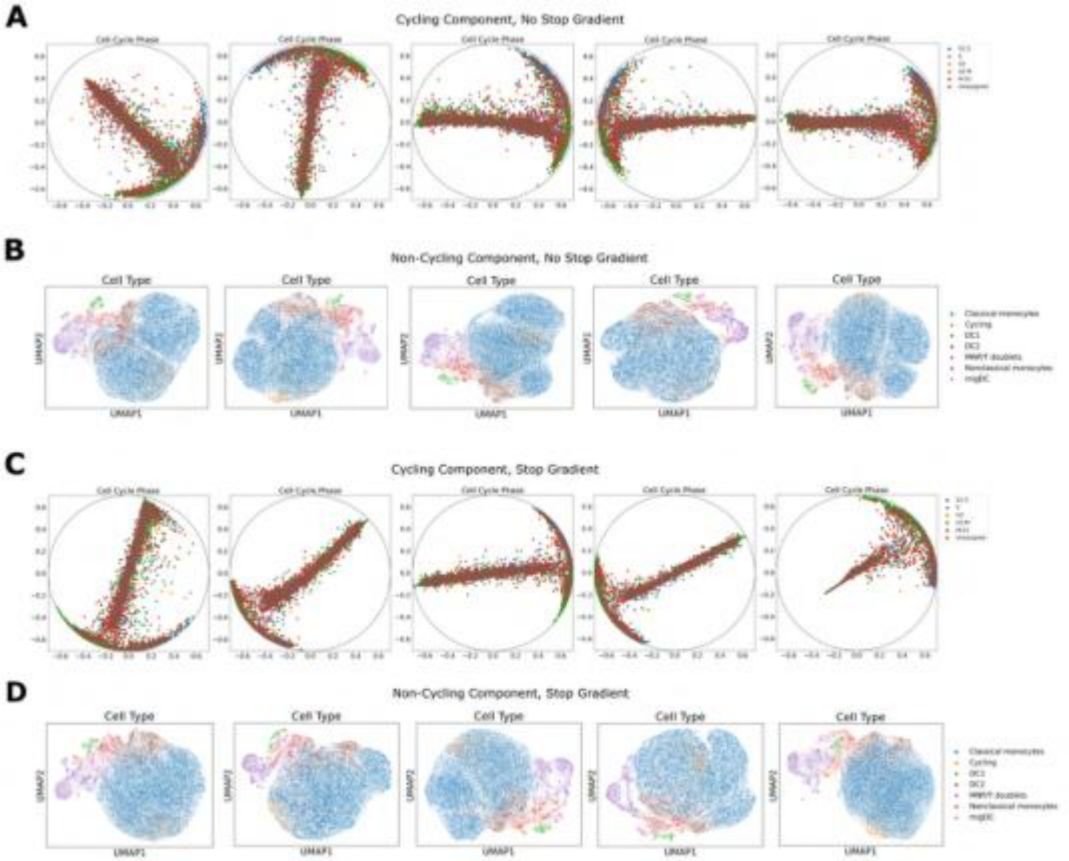

Figure R4: **Using no stop-gradient and stop-gradient on the immune cells dataset.** (**A**) The embeddings of the first component, $\mathbf{z}^1$, and (**B**) the embeddings of the second component $\mathbf{z}^2$ when stop-gradient is not used for five different runs of CellUntangler. (**C**) The embbeddings of the first component, $\mathbf{z}^1$ and (**D**) the embeddings of the second component, $\mathbf{z}^2$, when stop gradient is used for five different runs of CellUtangler.

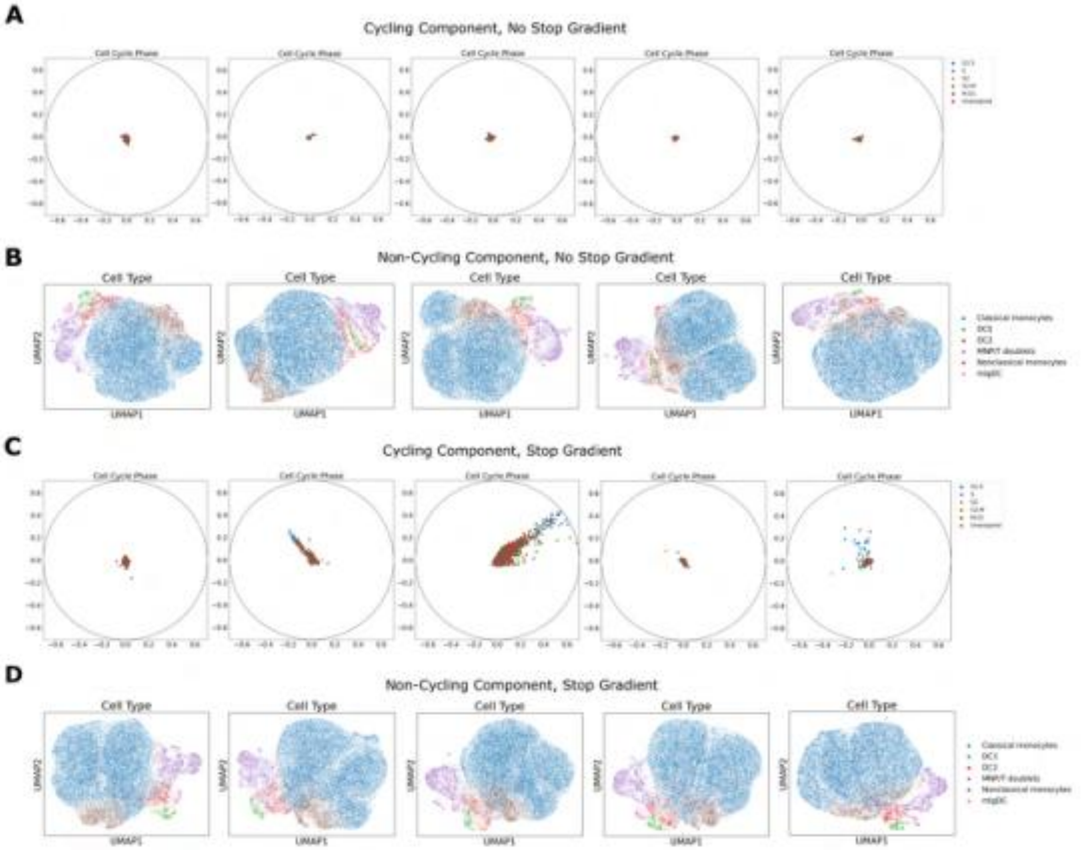

Figure R5: **Using no stop-gradient and stop-gradient on the immune cells dataset without the cycling cells.** (**A**) The embeddings of the first component, $\mathbf{z}^1$, and (**B**) the embeddings of the second component, $\mathbf{z}^2$ when stop-gradient is not used for five different runs of CellUntangler. (**C**) The embeddings of the first component, $\mathbf{z}^1$ and (**D**) the embeddings of the second component, $\mathbf{z}^2$, when stop gradient is used for different five runs of CellUntangler.

We applied CellUntangler to a dataset of mouse liver cells to separate the spatial and temporal signals into different embeddings, $\mathbf{z}^1$ and $\mathbf{z}^2$. We did this for both 50 zonated genes and 15 zonated genes. When using 50 zonated genes and no stop-gradient, the non-spatial component, $\mathbf{z}^2$ captured the circadian rhythm signal only and not the zonation signal (**Figure R6A**). Using stop-gradient, $\mathbf{z}^2$ also captured the circadian rhythm signal only and not the zonation signal (**Figure R6B**). However, when using only 15 zonation genes, without using stop-gradient allowed the zonation signal to be filtered out (**Figure R7A**), but using stop-gradient resulted in the zonation and circadian rhythm signals being captured in the

non-spatial component (**Figure R7B**). Because we use only 15 zonated genes, other such zonated genes will likely be present in the gene list not marked as zonated genes. By using stop-gradient though, $\mathbf{z}^1$ cannot learn from these other genes in our setting, resulting in $\mathbf{z}^2$ capturing some of the zonation signal.

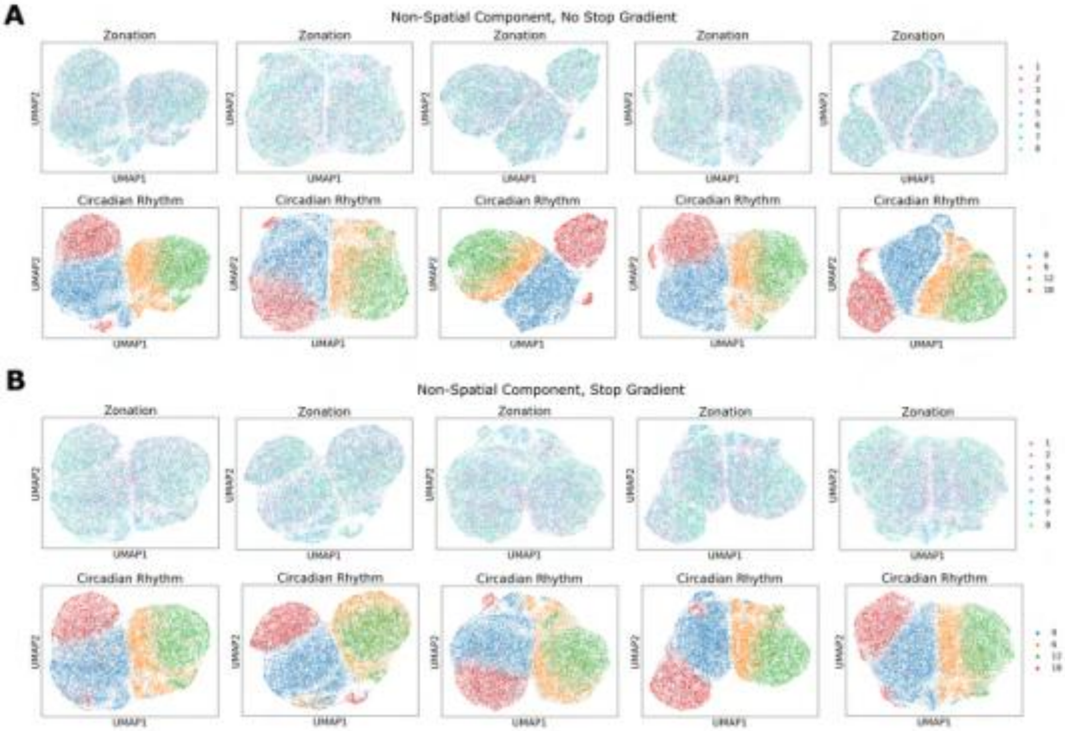

Figure R6: **Using no stop-gradient and stop-gradient on the mouse liver cells with 50 zonated genes.** (**A**) The embeddings of the second component, $\mathbf{z}^2$, when stop-gradient is not used and (**B**) when stop-gradient is used for five different runs of CellUntangler.

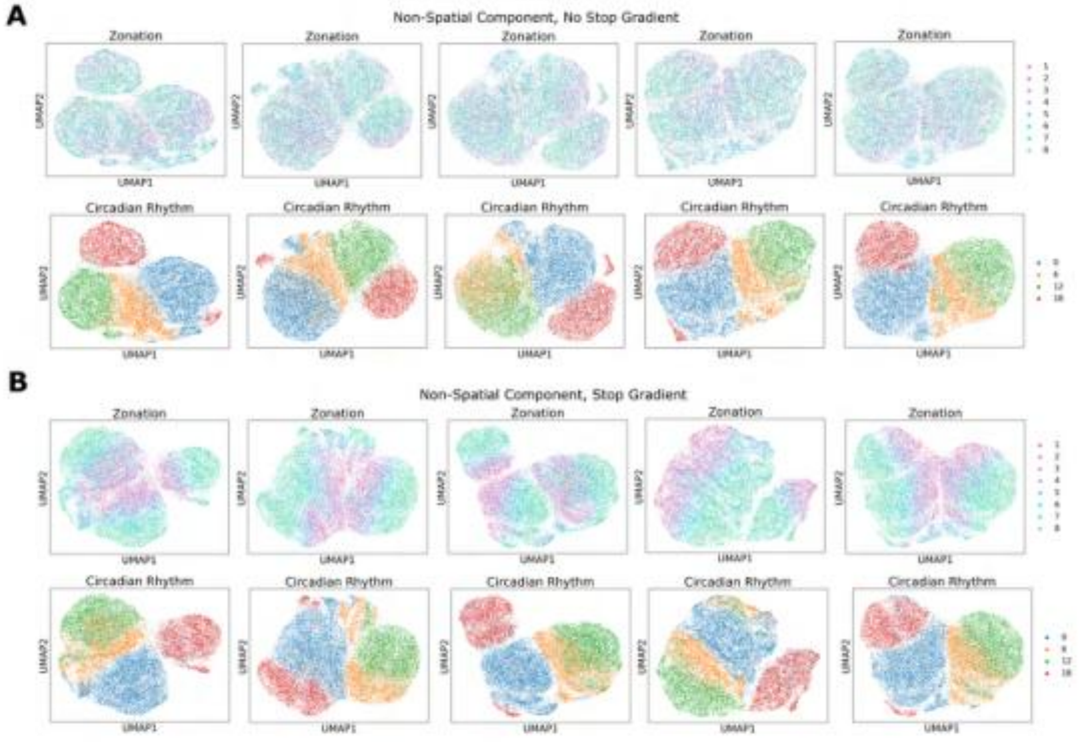

Figure R7: **Using no stop-gradient and stop-gradient on the mouse liver cells with 15 zonated genes.** (**A**) The embeddings of the second component, $\mathbf{z}^2$, when stop-gradient is not used and (**B**) when stop-gradient is used for five different runs of CellUntangler.

5. Overall, the paper is well presented and methodology described clearly and experiments demonstrate the utility of the model. The main limitation is that the paper is largely focused on cell cycle programs with the only exception the last example on zonation where I struggled to see, as clearly, the patterns discussed in the text. While cell cycle correction is important, it is not clear to what extent the implementation is geared toward cell cycle and further alternate examples would be appreciated.

We appreciate the Reviewer's recognition of the explanation of our methods and experiments. To help address the reviewer's concerns, we add results from reanalyzing the mouse liver data, using **50** zonation marker genes, instead of 15 marker genes. We also show the

enhancement and filtering of the zonation signal on gene *Pck1*, which is an independent rhythmic-zonated gene (as in Q3), while the *Hlf* gene we originally used is a rhythmic gene changing expression primarily with time (which unfortunately complicated the interpretation of the original **Figure 6**).

Importantly, we add the results from analyzing **a new mouse liver dataset**[4], to further demonstrate CellUntangler mapping cells to spatial tissue locations, and disentangling spatial information from non-spatial information (i.e., time). Importantly, CellUntangler enables us to do counterfactual reasoning (e.g., predicting the expression of genes of a cell, by setting it to the central location) for biological discovery. We add the results from analyzing this new mouse liver hepatocyte dataset as **Figure 6** in the updated manuscript.

CellUntangler is a general framework to capture and remove signals of interest in single-cell data. To further demonstrate the broad utility of CellUntangler on capturing and removing non-cell cycle signals, we used CellUntangler to analyze three signals. First, we used it to capture and remove the interferon response signal in the ovarian cancer tumor microenvironment. For this analysis, we used the set of 65 upregulated genes in human peripheral blood mononuclear cells (PBMCs) after interferon beta stimulation[2]. The interferon response gene signature has been shown to be upregulated in tumor cells, $CD4^+$ T cells, $CD8^+$ T cells, and NK cells[1]. We ran CellUntangler on the $CD8^+$ T cells, as in the original publications[1], and used the Euclidean space of dimension two to capture the interferon response signal, $\mathbf{z}^1 \in \mathbb{E}^2$, and the hyperbolic space of dimension two to capture the differentiation trajectory, $\mathbf{z}^2 \in \mathbb{H}^2$. In the first component ($\mathbf{z}^1$) (**Figure R8A**), cells were ordered by the interferon beta (IFN beta) score while in the second component ($\mathbf{z}^2$) (**Figure R8B**), cells separated by cell subtype and followed the expected organization: $CD8^+$ T naive central memory 1, $CD8^+$ T naive central memory 2, and $CD8^+$ T effector memory cells, followed by $CD8^+$ T cytotoxic, $CD8^+$ T dysfunctional early, and $CD8^+$ T dysfunctional late cells. Notably, $CD8^+$ T ISG early, $CD8^+$ T ISG late, and $CD8^+$ T ISG dysfunctional cells were scattered throughout the rest of the cells in the second component due to the interferon response signal being removed, suggesting that different $CD8^+$ subsets, such as naive central memory cells, cytotoxic cells, and dysfunctional cells contribute to the ISG cells.

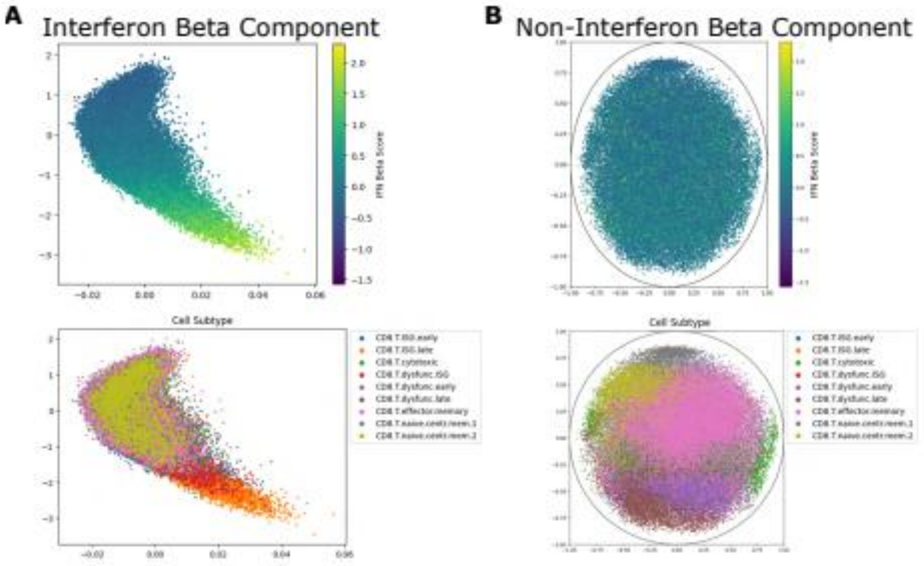

Figure R8: **Capturing and removing the interferon response signal in CD8$^+$ T cells from high-grade serous ovarian cancer patients.** (**A**) The embeddings of the first component, $\mathbf{z}^1 \in \mathbb{E}^2$, and (**B**) the embeddings of the second component, $\mathbf{z}^2 \in \mathbb{H}^2$.

We further investigated whether the interferon response signal is also upregulated in other cells of the tumor environment which were not reported to respond to interferon. We chose mast cells, dendritic cells, B cells, plasma B cells, and endothelial cells for this analysis. We used the Euclidean space of dimensionality two to capture the interferon response signal, $\mathbf{z}^1 \in \mathbb{E}^2$, and the Euclidean space of dimension ten to capture cell type, $\mathbf{z}^2 \in \mathbb{E}^{10}$. Again in the first component (**Figure R9A**), cells were ordered by the interferon beta score where we observed that some of these cells, the ones near the top, do exhibit an interferon response signal based on the higher interferon beta scores. In the second component (**Figure R9B**), cells separated by cell type. These results suggest that a broader range of cell types within the tumor microenvironment respond to the interferon signal.

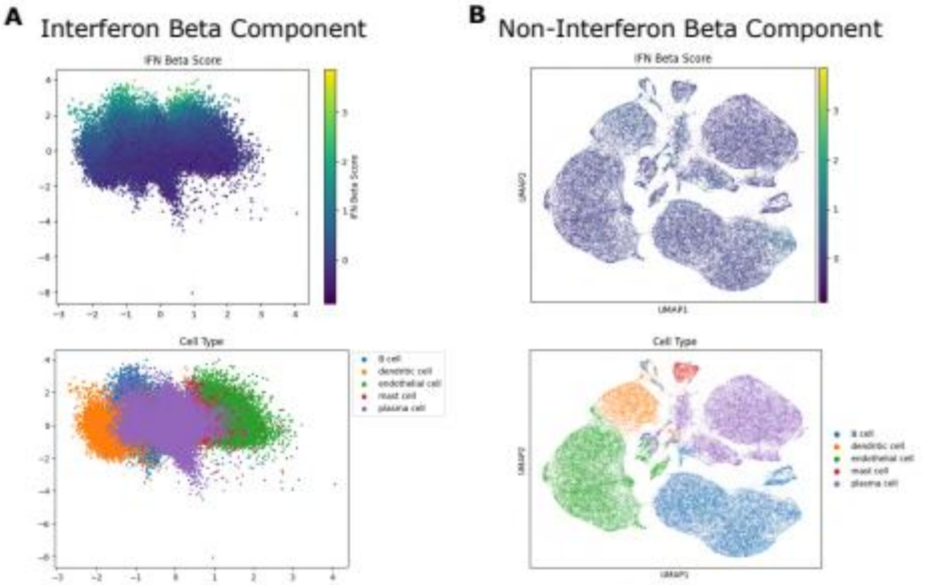

Figure R9: Capturing and removing the interferon response signal in a subset of cells from high-grade serous ovarian cancer patients. (**A**) The embeddings of the first component, $\mathbf{z}^1$, and (**B**) the embeddings of the second component, $\mathbf{z}^2$, projected to 2D using UMAP.

Finally, we ran CellUntangler on the full atlas of high-grade serous ovarian cancer cells. We used the Euclidean space of dimension two to capture the interferon beta response signal, $\mathbf{z}^1 \in \mathbb{E}^2$, and the Euclidean space of dimension ten to capture cell type, $\mathbf{z}^2 \in \mathbb{E}^{10}$. The first component, $\mathbf{z}^1$, captured the interferon beta response signal while the second component, $\mathbf{z}^2$, captured cell type. Visualization of $\mathbf{z}^2$ by individual cell type additionally showed how the different cell types exhibit a response to interferon beta. We present these results as panels **C-E** in **Figure 7** of the updated manuscript.

Next, we ran CellUntangler on the dataset of PBMCs stimulated with interferon beta[2]. We used cell type marker genes[9–11] to separate the cell type and stimulation signals into difference components, $\mathbf{z}^1 \in \mathbb{E}^{10}$ and $\mathbf{z}^2 \in \mathbb{E}^5$ respectively. We visualized $\mathbf{z}^1$ and $\mathbf{z}^2$ to confirm that the cell type and stimulation signals were separately captured. To assess how well $\mathbf{z}^1$ captured the cell type signal, we measured the k-NN accuracies on cell type using $\mathbf{z}^1$. Interferon beta stimulation activates the JAK-STAT signalling pathway in cells. However, this can be obscured by other factors (e.g., cell types), resulting in control and stimulated cells having similar JAK-STAT scores (mean of -0.111 in the unstimulated cells and 0.0972 in the stimulated cells) although statistically significantly different (Mann-Whitney U test p-value (two-sided): 4.619e-252). We used CellUntangler to obtain a gene reconstruction with the cell type signal filtered out by passing $\mathbf{z}^2$ to CellUntangler's decoder. Using this

reconstruction, the stimulated cells have a JAK-STAT score[12] more noticeably higher than the control cells (mean of -0.587 in the unstimulated cells and 0.516 in the stimulated cells, Mann-Whitney U test p-value (two-sided): 0.0). Next, we examined the genes upregulated due to interferon beta stimulation. We first performed PCA on $\mathbf{z}^2$ then selected one hundred stimulated cells and one hundred control cells based on PC1 and PC2. We reconstructed $\mathbf{z}^2$ for each of these cells using four PCs and passed the reconstructed $\mathbf{z}^2$ to CellUntangler's decoder to obtain the reconstructed gene expression. Control cells and stimulated cells were grouped into one hundred pairs and we subtracted the reconstructed control expression from the reconstructed stimulated expression for each pair. We obtained the top 50 genes with the largest positive difference for each pair and counted the frequency of these genes. Genes present in the cell type markers list used with CellUntangler were removed. As expected, genes such as *ISG20* and *IFIT1* were present in the top 50 genes with the highest frequency. Note, 32/50 genes were among the interferon beta stimulation signature we used for analyzing the ovarian cancer dataset (Fisher's exact test p-value (one-sided): 3.185e-66). We add these results as **Figure S7** to the updated manuscript.

We finally used CellUntangler to analyze mast cells in the context of eosinophilic esophagitis (EoE), a chronic inflammatory disease of the esophagus[3]. Mast cells are enriched in the esophageal mucosa of patients with active EoE, compared to healthy individuals. However, as tissue-resident cells, mast cells also exhibit a strong tissue dissociation signal, complicating the analysis of their function. Here we used CellUntangler to capture and remove the tissue-dissociation signal using a set of 34 tissue-dissociation signature genes[13]. We used $\mathbf{z}^1 \in \mathbb{E}^2$ to capture the tissue-dissociation signal and $\mathbf{z}^2 \in \mathbb{E}^{10}$ to capture other signals such as disease. As expected, we can clearly see $\mathbf{z}^1$ is correlated with the tissue-dissociation signature score[14] (calculated by Scanpy). In $\mathbf{z}^2$, cells were grouped by disease status but not by the tissue-dissociation signal, suggesting cells from active EoE change their gene expression, compared to mast cells from healthy individuals. In contrast, using the standard pipeline, with batch correction on 10x Genomics Chemistry version using Harmony[15], we did not observe this pattern because of the strong tissue dissociation signal in the dataset. We next wanted to detect the upregulated genes in mast cells from active EoE patients, compared to those from healthy individuals. This can be done by extending deep generative models for differential expression analysis[16]. For simplicity, we first did a PCA analysis of $\mathbf{z}^2$, and found that PC1, PC3, and PC4 captured the disease signal. We thus picked one hundred points from the 'healthy' region based on PC1-4 (we set PC2 to 0) and one hundred points from the 'Active' region based on PC1-4 (we set PC2 to 0). We grouped these two hundred points into one hundred pairs composed of one healthy cell and one active cell and then used CellUntangler decoder to decode the reconstructed latent coordinates for each pair, and calculated their decoded gene expression difference. We obtained the top 50 genes with the largest positive difference (active - healthy) for each pair and counted the frequency of these genes. We used the top 20 genes with the highest frequency to compute 'mast cell activation' signature scores[14] in individual cells, and found that mast cells from active EoE patients upregulated this gene signature compared to those from healthy or remission patients. These top upregulated genes include *ITGA2B*. *ITGA2B* is a well-known marker of

megakaryocytes/platelets. It has also been shown to be expressed in bone marrow-derived mast cells and modulates chronic inflammation[17], representing a potentially important factor in regulating esophageal mast cells. We include these results as **Figure S6** of the revised manuscript.

Response to Reviewer 2

The article introduces CellUntangler, a tool based on deep generative models. CellUntangler is innovative in its multi-space modeling using both hyperbolic and Euclidean spaces, which allows for more precise separation and analysis of different biological signals. Additionally, the authors demonstrate the tool's broad applicability through validation on multiple datasets using various methods, such as KNN classification and validation of marker gene expression. This makes CellUntangler a potentially important tool in the field of single-cell data analysis. However, there are still some areas for improvement:

We thank the reviewer for the feedback and are heartened by the reviewer's description of CellUntangler as an innovative and potentially important tool.

1. Although the article mentions the limitations of some other tools, a more comprehensive comparative analysis, including performance metrics and scope of application, could further highlight the advantages of CellUntangler.

We thank the reviewer for the suggestion of a more comprehensive analysis to highlight the advantages of CellUntangler. We report the iLISI graph with respect to the **cell cycle phase** to measure how well the cell cycle is removed. To measure how well the identity of a cell as a knockout (Ago2KO) cell or a wildtype (WT) cell is revealed, we report the NMI score, ARI score, and cLISI graph, all with respect to whether the cell is an Ago2KO or WT cell. We do this for CellUntangler, Seurat, SiFT, scPrisma, and Revelio. As a baseline, we compute the metrics after removing the cell cycle genes in our list, Baseline 1, and after removing the Revelio genes, Baseline 2. These metrics range from 0 to 1 with higher scores being better. We use the package scib[18] to compute these metrics. We add a **supplementary table** to summarize the strength and limitations of each method (**Table R1**) which is added as **Table S2** to the manuscript, and their performance on inferring and removing the cell-cycle signal, on the HeLa dataset (**Table R2**, added as **Table S3** to the manuscript).

	CellUntangler	tricycle	scPrisma	Revelio	Seurat	SiFT	DeepCycle	reCAT	Cyclum	ccRemover	SC1CC
Cell cycle pseudotime or ordering	Yes	Yes	Yes	Yes	No[a]	No	Yes	Yes	Yes	No	Yes
Filter cell cycle	Yes	No	Yes	Yes	Yes	Yes	No	No	Yes	Yes	No
Applicable to non-cyclic signals	Yes	No	Yes	No	Yes	Yes	No	No	No	No	No

Table R1: **Scope of application for CellUntangler and other methods, related to Figure 2.** We compared the cell cycle pseudotime and cell cycle filtering of CellUntangler with representative and well-maintained models (tricycle, scPrisma, Revelio, Seurat, and SiFT). DeepCycle was not used in our experiments due to the requirement of spliced and unspliced counts. [a]Although Seurat does not provide a continuous cell cycle pseudotime or ordering, it does provide gene signature scores and assign discrete cell cycle phases. scPrisma does not perform well in cases where only a subset of cells are cycling (**Figure 3F**).

	CellUntangler	Revelio	scPrisma	SiFT	Seurat	Baseline 1	Baseline 2
iLISI	0.592	**0.654**	0.551	0.646	0.649	0.442	0.457
ARI Leiden (15)	0.472	**0.515**	0.280	0.167	0.347	0.148	0.152
NMI Leiden (15)	**0.406**	0.400	0.262	0.231	0.334	0.196	0.206
ARI Louvain (15)	**0.468**	0.299	0.228	0.142	0.308	0.166	0.167
NMI Louvain (15)	**0.395**	0.310	0.217	0.181	0.339	0.162	0.237
ARI Leiden (50)	**0.562**	0.313	0.272	0.193	0.281	0.117	0.166
NMI Leiden (50)	**0.467**	0.275	0.267	0.227	0.320	0.147	0.223
ARI Louvain (50)	**0.489**	0.231	0.271	0.171	0.259	0.080	0.147
NMI Louvain (50)	**0.420**	0.275	0.267	0.206	0.319	0.060	0.159
cLISI	**0.834**	0.404	0.321	0.270	0.355	0.370	0.452

Table R2: Benchmarking of cell cycle signal removal and revealing of cell identity as a knockout (Ago2KO) cell or wild type (WT) cell on the HeLa data. Both Leiden and Louvain clustering were used. The number in brackets indicates the number of neighbors used. For the rest of the metrics, the default values were used. The average over ten runs for each of these metrics is reported for CellUntangler. Baseline 1 removes the cell cycle genes in our list and Baseline 2 removes the Revelio genes.

For the mESCs dataset, we report (as **Table S4**) the correlation between the ground truth pseudotime and the predicted pseudotime to measure and compare how well CellUntangler, tricycle, scPrisma, and Revelio capture the cell cycle (**Table R3**).

	CellUntangler	tricycle	scPrisma	Revelio
Cell cycle correlation	**0.805**	0.516	0.590	0.584

Table R3: Comparison of cell cycle reconstruction for the mESC dataset. The transcriptional phase output by DeepCycle[19], as analyzed by the original authors of this dataset, is used as the ground truth. The average correlation over ten runs is reported for CellUntangler.

Regarding the scope of application, as addressed in Comment 5 of Reviewer 1, we ran CellUntangler on a dataset of human peripheral blood mononuclear cells (PBMCs) stimulated

with interferon beta. We used cell type marker genes to separate the cell type and non-cell type signals into different embeddings, $\mathbf{z}^1$ and $\mathbf{z}^2$. For the cell type component, $\mathbf{z}^1$, we measured how well cell type is captured by running k-NN on $\mathbf{z}^1$. To analyze the non-cell type component, $\mathbf{z}^2$, we reconstructed the gene expression using $\mathbf{z}^2$ to find genes upregulated due to interferon beta stimulation. These results are added as **Figure S7** in the updated manuscript.

We used CellUntangler to capture the interferon response signal in cells of the high-grade serous ovarian cancer microenvironment to understand the broad cell types that respond to interferons. We separately ran CellUntangler on a subset of CD8 T cells, **Figure R8**), other non-T cells such as mast cells (**Figure R9**), and also on the entire atlas. The results on the entire atlas are added as panels **C-E** in **Figure 7** of the updated manuscript.

We also ran CellUntangler on mast cells from patients in active eosinophilic esophagitis (EoE), those in remission, and healthy individuals. We examined which genes were upregulated in active EoE by disentangling the cell dissociation signal from disease status. These results are added as **Figure S6** in the updated manuscript.

Finally, we added a new main figure, **Figure 6**, from analyzing a new mouse hepatocyte dataset from mice in fed, fasted, and starvation states. CellUntangler successfully captured the spatial zonation signal, and one latent dimension of $\mathbf{z}^1$ can be considered as the "pseudospace", while $\mathbf{z}^2$ captured the temporal information only, but not the spatial information.

2. The article does not provide a detailed discussion on the computational efficiency and scalability of CellUntangler, especially on large-scale datasets. This is crucial for the practical application of the tool, particularly when dealing with high-throughput single-cell data.

We'd like to thank the reviewer for bringing up the important question about the computational efficiency and scalability. As a deep learning model trained using mini-batch (each step using 128 cells for training by default), CellUntangler is scalable to large datasets, as for other deep-learning based models such as scVI. Similar to other deep-learning based models, the computational complexity could be higher by using more genes for analysis. We use the retina dataset with 121,309 cells (2684 genes, highly variable genes with cell cycle genes added in) and the high-grade serous ovarian cancer dataset with $\sim$1M cells to demonstrate the scalability of CellUntangler. We add a section to the main text titled "**CellUntangler effectively captures and removes diverse biological signals and scales to large datasets**" to discuss the performance on large-scale datasets. To study how the performance of CellUntangler changes with the number of cells, we subsampled an increasingly large number of cells (e.g., 5000, 10,000, 20,000, 40,000, 80,000, 160,000, 500,000, 750,000, 927,205) from the atlas of ovarian cancer cells and measured the runtime of CellUntangler on CPU and GPU. These plots become panels **A** and **B** in **Figure 7** of the updated manuscript.

We first ran CellUntangler on the retina cell atlas of 121,309 cells. The dataset contains

various cell types, including cycling and non-cycling microglia. We used CellUntangler to capture the cell cycle in the hyperbolic space with the rotated wrapped normal distribution, $\mathbf{z}^1 \in \mathbb{H}^2$, and the Euclidean space to capture non-cell cycle specific signals, $\mathbf{z}^2 \in \mathbb{E}^{10}$. In the cell cycle component, the cycling microglia are placed along the edge of the Poincaré disc. After recentering the origin, the cycling microglia form a circle around the non-cycling cells. In the non-cell cycle component, cells separate by cell type. As expected, the cycling microglia express cell cycle genes, as well as some astrocytes placed with the cycling microglia. Note, these cycling astrocytes **cannot be identified by jointly clustering the full dataset** using the 'standard' scRNA-seq data analysis pipeline. As expected, the cycling microglia are integrated with the non-cycling microglia in $\mathbf{z}^2$. These results have been added as **Figure S5** of the updated manuscript. The runtime was approximately six hours for 500 epochs on CPU and approximately four hours for 500 epochs on an A100 GPU. For datasets with a large number of cells though, fewer epochs may be necessary. We obtained similar results when running CellUntangler on the retina cells for only 50 epochs (**Figure R10**).

Finally, on the large high-grade serous ovarian cancer dataset with 927,205 cells and 3038 genes, we were able to separately capture the interferon beta response and cell type signals in 50 epochs. The average runtime, over three runs, was **94.84 minutes** on an A100 GPU. We believe this demonstrates that CellUntangler is scalable to large-scale single-cell data. We add the results from analyzing the high-grade serous ovarian cancer as panels **C-E** in **Figure 7** of the updated manuscript.

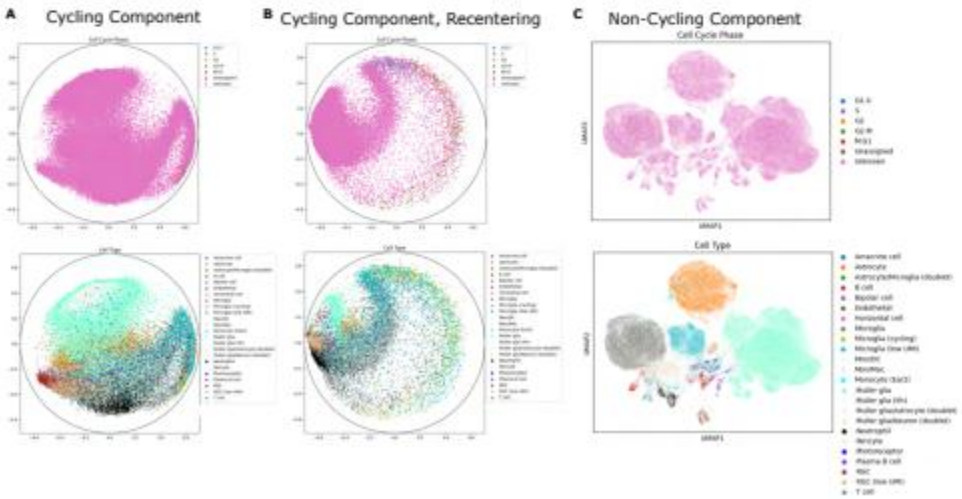

Figure R10: Capturing and removing the cell cycle signal in the mouse retinal dataset, only training CellUntangler for 50 epochs. (**A**) The embeddings of the first component, $\mathbf{z}^1$, before recentering and (**B**) after recentering the origin to (-0.5, 0.1), when running CellUntangler for 50 epochs. (**C**) The embeddings of the second component, $\mathbf{z}^2$, projected to 2D using UMAP, when running CellUntangler for 50 epochs.

References

1. Vázquez-García, I. *et al.* Ovarian cancer mutational processes drive site-specific immune evasion. *Nature* **612**, 778–786 (2022).

2. Kang, H. M. *et al.* Multiplexed droplet single-cell RNA-sequencing using natural genetic variation. *Nature Biotechnology* **36**, 89–94 (2018).

3. Ding, J. *et al.* An esophagus cell atlas reveals dynamic rewiring during active eosinophilic esophagitis and remission. *Nature Communications* **15**, 3344 (2024).

4. Okada, J. *et al.* Spatial hepatocyte plasticity of gluconeogenesis during the metabolic transitions between fed, fasted and starvation states. *Nature Metabolism* **7**, 1073–1091 (2025).

5. Benhar, I. *et al.* Temporal single-cell atlas of non-neuronal retinal cells reveals dynamic, coordinated multicellular responses to central nervous system injury. *Nature Immunology* **24**, 700–713 (2023).

6. Droin, C. *et al.* Space-time logic of liver gene expression at sub-lobular scale. *Nature Metabolism* **3**, 43–58 (2021).

7. Watson, B. R. *et al.* Spatial transcriptomics of healthy and fibrotic human liver at single-cell resolution. *Nature Communications* **16**, 319 (2025).

8. Lange, M. *et al.* CellRank for directed single-cell fate mapping. *Nature Methods* **19**, 159–170 (2022).

9. Hao, Y. *et al.* Integrated analysis of multimodal single-cell data. *Cell* **184**, 3573–3587 (2021).

10. Zhang, X. *et al.* CellMarker: a manually curated resource of cell markers in human and mouse. *Nucleic Acids Research* **47**, D721–D728 (2019).

11. Hu, C. *et al.* CellMarker 2.0: an updated database of manually curated cell markers in human/mouse and web tools based on scRNA-seq data. *Nucleic Acids Research* **51**, D870–D876 (2023).

12. Hu, X., Li, J., Fu, M., Zhao, X. & Wang, W. The jak/stat signaling pathway: from bench to clinic. *Signal Transduction and Targeted Therapy* **6**, 402 (2021).

13. van den Brink, S. C. *et al.* Single-cell sequencing reveals dissociation-induced gene expression in tissue subpopulations. *Nature Methods* **14**, 935–936 (2017).

14. Tirosh, I. *et al.* Dissecting the multicellular ecosystem of metastatic melanoma by single-cell RNA-seq. *Science* **352**, 189–196 (2016).

15. Korsunsky, I. *et al.* Fast, sensitive and accurate integration of single-cell data with harmony. *Nature Methods* **16**, 1289–1296 (2019).

16. Boyeau, P. *et al.* An empirical bayes method for differential expression analysis of single cells with deep generative models. *Proceedings of the National Academy of Sciences* **120**, e2209124120 (2023).

17. Oki, T. *et al.* Evidence that integrin αiibβ3-dependent interaction of mast cells with fibrinogen exacerbates chronic inflammation. *Journal of Biological Chemistry* **284**, 31463–31472 (2009).

18. Luecken, M. D. *et al.* Benchmarking atlas-level data integration in single-cell genomics. *Nature methods* **19**, 41–50 (2022).

19. Riba, A. *et al.* Cell cycle gene regulation dynamics revealed by rna velocity and deep-learning. *Nature Communications* **13**, 2865 (2022).

Referees' report, second round of review

Reviewer 1:

The authors have comprehensively and thoroughly responded to my questions and gone beyond in some instances particularly with the additional stop gradient experiments. I have no further concerns. I hope my initial queries did not cause too much anxiety and that the authors recognise that their efforts since have significantly improved the paper.

Reviewer 2:

CellUntangler, proposed in this manuscript, is a single-cell data analysis tool based on deep generative models. Its core innovation lies in the design of "latent subspaces with multiple geometric structures," which enables the accurate separation of different biological signals (such as cell cycle, spatial zonation, and interferon response). Addressing the limitations of existing methods—including "single-signal targeting" and "heavy reliance on assumptions"—this tool offers flexible signal capture and filtering capabilities, demonstrating potential in cell cycle disentanglement, spatial-temporal signal separation, and scalability for large datasets. Below are suggested revision directions for reference:

1. The manuscript selects geometric spaces for different signals (e.g., hyperbolic

space with RoWN for cell cycle, Euclidean space for interferon response), yet fails to verify the "necessity of such selections" through controlled experiments. For example, in the mESC dataset, if the latent space for cell cycle is switched from hyperbolic space to Euclidean space, will the cell cycle correlation decrease significantly? If the difference is negligible, the advantage of using hyperbolic space will be difficult to justify.

2. The authors demonstrate the independence of latent subspaces through visualization (e.g., in Figure R2, z1 clusters cells by cell cycle phase while z2 clusters cells by genotype), but lack quantitative metrics. No Mutual Information (MI) or cosine similarity between z1 and z2 has been calculated, making it impossible to objectively measure the "impact of the stop-gradient strategy on the degree of disentanglement." For instance, in the pancreatic dataset, after applying the stop-gradient strategy, is the Mutual Information between z1 (cell cycle) and z2 (differentiation trajectory) significantly lower than that without the stop-gradient strategy?

3. The paper demonstrates a variant of the VAE framework for signal decomposition with single-cell data. The latest deep generative models, scCross (PMID: 39075536) and scGlue (PMID: 35501393), are currently available. It is recommended that the author compare your model with these two methods.

Authors’ response to the second round of review

Response to Reviewers

We thank the reviewers for their time and thoughtful comments on our manuscript. We greatly appreciate the specific comments raised, which are addressed in detail below. We first highlight the key revisions and then address each comment in a point-by-point manner.

The key highlights in our revision are:

1. Additional experiments to verify our selection of the hyperbolic space to capture the cell cycle signal over the Euclidean space. We compared using the hyperbolic space against using the Euclidean space with a dimension of two and the Euclidean space with a dimension of ten for the HeLa dataset, the mouse embryonic stem cells (mESCs) dataset, and the immune cells dataset. The results for the HeLa dataset are presented in **Figure S1** and **Table S3** of the updated manuscript. The results for the mESCs dataset are presented in **Figure S2** and **Table S4**, and the results for the immune cells dataset are presented in **Figure S3**.

2. Measured the Mutual Information (MI) to assess the effect of using CellUntangler with stop-gradient.

3. Acknowledged scCross and scGlue and explained why they were not included in our benchmarking in the **Limitations of the study** section.

Response to Reviewer 1

The authors have comprehensively and thoroughly responded to my questions and gone beyond in some instances particularly with the additional stop gradient experiments. I have no further concerns. I hope my initial queries did not cause too much anxiety and that the authors recognise that their efforts since have significantly improved the paper.

We sincerely thank the reviewer for their thoughtful comments and kind words. We are glad that our additional experiments and revisions have addressed your questions, and we greatly appreciate your guidance in improving the clarity and rigor of our work.

Response to Reviewer 2

CellUntangler, proposed in this manuscript, is a single-cell data analysis tool based on deep generative models. Its core innovation lies in the design of "latent subspaces with multiple geometric structures," which enables the accurate separation of different biological signals (such as cell cycle, spatial zonation, and interferon response). Addressing the limitations of existing methods—including "single-signal targeting" and "heavy reliance on assumptions"—this tool offers flexible signal capture and filtering capabilities, demonstrating potential in cell cycle disentanglement, spatial-temporal signal separation, and scalability for large datasets. Below are suggested revision directions for reference:

We thank the reviewer for the feedback and are heartened by the reviewer's description of CellUntangler as an innovative and potentially important tool.

1. The manuscript selects geometric spaces for different signals (e.g., hyperbolic space with RoWN for cell cycle, Euclidean space for interferon response), yet fails to verify the "necessity of such selections" through controlled experiments. For example, in the mESC dataset, if the latent space for cell cycle is switched from hyperbolic space to Euclidean space, will the cell cycle correlation decrease significantly? If the difference is negligible, the advantage of using hyperbolic space will be difficult to justify.

We thank the reviewer for their question regarding the choice of geometric space. To address this, we compared using the Euclidean space with dimension two ($\mathbb{E}^2$) and the Euclidean space with dimension ten ($\mathbb{E}^{10}$) against using the hyperbolic space with the RoWN ($\mathbb{H}^2$) to capture the cell cycle signal.

For the HeLa dataset, we used the hyperbolic space with the RoWN to capture the cell cycle signal, $\mathbf{z}^1 \in \mathbb{H}^2$, and the Euclidean space to capture non-cell cycle specific signals, $\mathbf{z}^2 \in \mathbb{E}^{10}$. In $\mathbf{z}^1$, cells separated by cell cycle phase (**Figure R1A**), whereas in $\mathbf{z}^2$, cells separated by wild type and knockout (**Figure R1B**). When using $\mathbf{z}^1 \in \mathbb{E}^2$ to capture the cell cycle signal, cells still separated by cell cycle phase. However, the gap in the middle of the cycling cells may be hard to observe as cells were encouraged to be placed near the origin, (0, 0), due to the KL-divergence term (**Figure R1C**). We find that using the hyperbolic space provides a nicer visualization since cycling cells were placed near the edge of the Poincaré disc resulting in a clearer circle. Again, when we used $\mathbf{z}^2 \in \mathbb{E}^{10}$, cells separated by wild type and knockout (**Figure R1D**). Visualizations of $\mathbf{z}^1 \in \mathbb{E}^{10}$ projected to 2D using PCA (**Figure R1E**) and $\mathbf{z}^2 \in \mathbb{E}^{10}$ (**Figure R1F**) were similar to those for $\mathbf{z}^1 \in \mathbb{E}^2$ and $\mathbf{z}^2 \in \mathbb{E}^{10}$.

We measured the cell cycle correlation by measuring the angle from the origin, (0, 0), for each of the cells. This angle was considered the cell's pseudotime. Using the hyperbolic space resulted in the highest cell cycle correlation (**Figure R1G**, **Table R1**). When comparing k-NN accuracies on wild type and knockout, using the hyperbolic space had a similar performance to the Euclidean space with dimension two and better performance than the Euclidean space with dimension ten (**Figure R1H**). We updated **Figure S1** to include a visualization of the cell cycle component using $\mathbf{z}^1 \in \mathbb{E}^2$ and $\mathbf{z}^1 \in \mathbb{E}^{10}$ from **Figure R1C** and **Figure R1E** to become panels **D** and **E**. We added **Figure R1G** and **Figure R1H** as panels **F** and **G**.

For other performance metrics, using the hyperbolic space with the RoWN tended to result in similar or better performance as using the Euclidean space (**Table R2**). We updated **Table S3** to include results for $\mathbf{z}^1 \in \mathbb{E}^2$ and $\mathbf{z}^1 \in \mathbb{E}^{10}$.

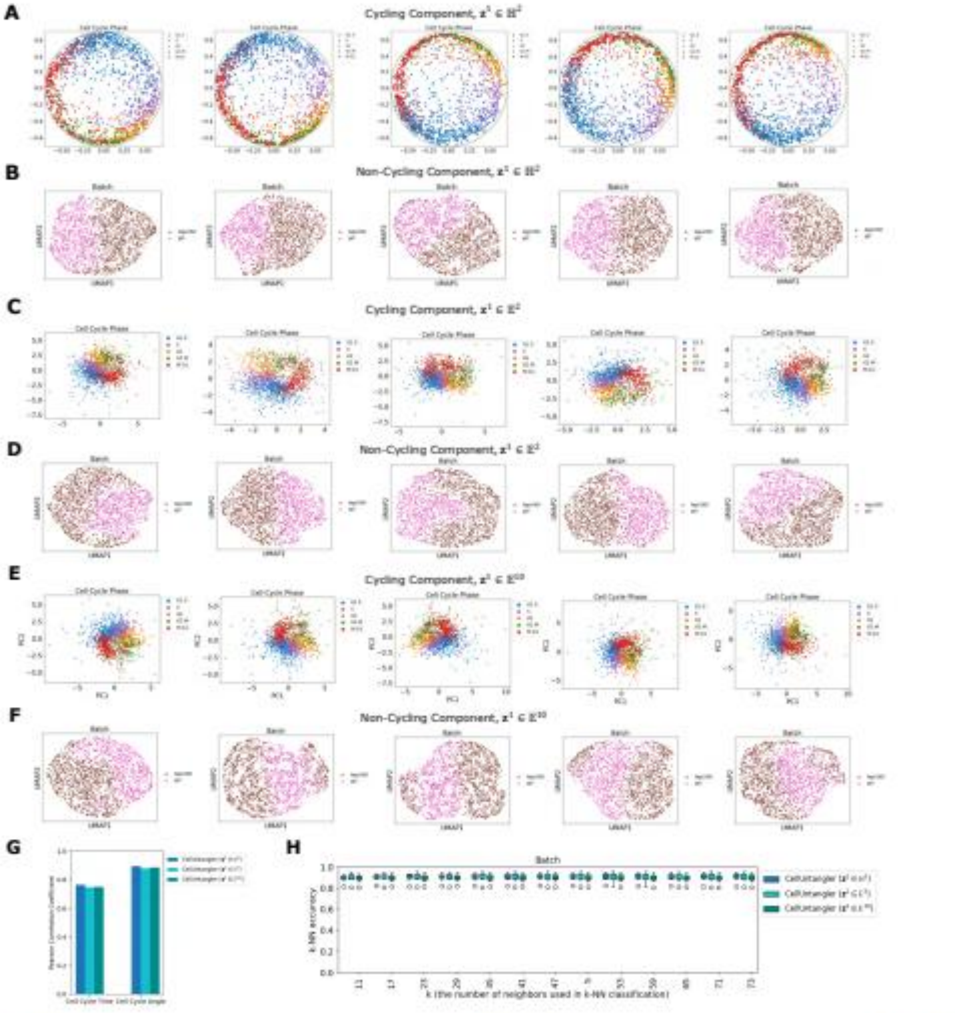

Figure R1: **Using the hyperbolic space or the Euclidean space to capture the cell cycle signal.** (**A**) The embeddings of the first component, $\mathbf{z}^1$, and (**B**) the embeddings of the second component, $\mathbf{z}^2$, when $\mathbf{z}^1 \in \mathbb{H}^2$ and $\mathbf{z}^2 \in \mathbb{E}^{10}$ for five different runs of CellUntangler. (**C**) The embeddings of the first component, $\mathbf{z}^1$, and (**D**) the embeddings of the second component, $\mathbf{z}^2$, when $\mathbf{z}^1 \in \mathbb{E}^2$ and $\mathbf{z}^2 \in \mathbb{E}^{10}$ for five different runs of CellUntangler. (**E**) The embeddings of the first component, $\mathbf{z}^1$, and (**F**) the embeddings of the second component, $\mathbf{z}^2$, when $\mathbf{z}^1 \in \mathbb{E}^{10}$ and $\mathbf{z}^2 \in \mathbb{E}^{10}$ for five different runs of CellUntangler. (**G**) Comparison of cell cycle reconstruction. (**H**) Accuracy of k-nearest neighbors on wild type and knockout cells using $\mathbf{z}^2$ from CellUntangler when using the hyperbolic space or the Euclidean space for $\mathbf{z}^1$. Boxplots depict the medians and the interquartile ranges (IQR). The whiskers are the lowest datum still within 1.5 IQR of the lower quartile and the highest datum still within 1.5 IQR of the upper quartile. Individual points below and above the whiskers indicate outliers.

	$\mathbf{z}^1 \in \mathbb{H}^2$	$\mathbf{z}^1 \in \mathbb{E}^2$	$\mathbf{z}^1 \in \mathbb{E}^{10}$
Cell Cycle Time	**0.763**	0.746	0.749
Cell Cycle Angle	**0.893**	0.878	0.885

Table R1: **Comparison of cell cycle reconstruction for the HeLa data.** The cell cycle time and the cell cycle angle output by Revelio[1], as analyzed by the original authors of this dataset is used as the ground truth. The average correlation over ten runs is reported for CellUntangler.

	$\mathbf{z}^1 \in \mathbb{H}^2$	$\mathbf{z}^1 \in \mathbb{E}^2$	$\mathbf{z}^1 \in \mathbb{E}^{10}$
iLISI	0.592	0.611	**0.618**
ARI Leiden (15)	**0.472**	0.446	0.399
NMI Leiden (15)	**0.406**	0.387	0.346
ARI Louvain (15)	**0.468**	0.435	0.348
NMI Louvain (15)	**0.395**	0.364	0.303
ARI Leiden (50)	**0.562**	0.548	0.495
NMI Leiden (50)	**0.467**	0.462	0.412
ARI Louvain (50)	0.489	**0.529**	0.505
NMI Louvain (50)	0.420	**0.442**	0.424
cLISI	**0.834**	0.813	0.812

Table R2: **Benchmarking of cell cycle signal removal and revealing of cell identity as a knockout (Ago2KO) cell or wild type (WT) cell on the HeLa data.** iLISI is reported with respect to cell cycle phase where values closer to 1 indicate better cell cycle removal. Both Leiden and Louvain clustering were used. The number in brackets indicates the number of neighbors used. For the rest of the metrics, the default values were used. The average over ten runs for each of these metrics is reported for CellUntangler. iLISI: integration local inverse Simpson's index; ARI: adjusted Rand index; NMI: normalized mutual information; cLISI: cell-type local inverse Simpson's index. The higher the better for these metrics.

For the mouse embryonic stem cells (mESC) dataset, we again used the hyperbolic space with the RoWN to capture the cell cycle signal, $\mathbf{z}^1 \in \mathbb{H}^2$, and the Euclidean space to capture non-cell cycle specific signals, $\mathbf{z}^2 \in \mathbb{E}^{10}$. As expected, in $\mathbf{z}^1$, cells were ordered by transcriptional phase, the cell cycle pseudotime output by DeepCycle[2], from $\theta = 0.0$ to $\theta = 1.0$ (**Figure R2A**), while in $\mathbf{z}^2$, cells were mixed by transcriptional phase (**Figure R2B**). When using $\mathbf{z}^1 \in \mathbb{E}^2$ and $\mathbf{z}^2 \in \mathbb{E}^{10}$, cells were also ordered by transcriptional phase in $\mathbf{z}^1$ (**Figure R2C**) and mixed by transcriptional phase in $\mathbf{z}^2$ (**Figure R2D**). When using $\mathbf{z}^1 \in \mathbb{E}^{10}$ and $\mathbf{z}^2 \in \mathbb{E}^{10}$, the gap in the middle of the cycling cells may be hard to observe in $\mathbf{z}^1$ (**Figure R2E**). Cells were mixed by transcriptional phase in $\mathbf{z}^2$ (**Figure R2F**). We updated **Figure S2** to include a visualization of the cell cycle component using $\mathbf{z}^1 \in \mathbb{E}^2$ and $\mathbf{z}^1 \in \mathbb{E}^{10}$ from **Figure R2C** and **Figure R2E** to become panels **F** and **G**.

The cell cycle correlation increased when using the Euclidean space with a dimension of two to capture the cell cycle signal and remained about the same when using the Euclidean space

with a dimension of ten (**Table R3**). We updated **Supplementary Table S4** to include these results.

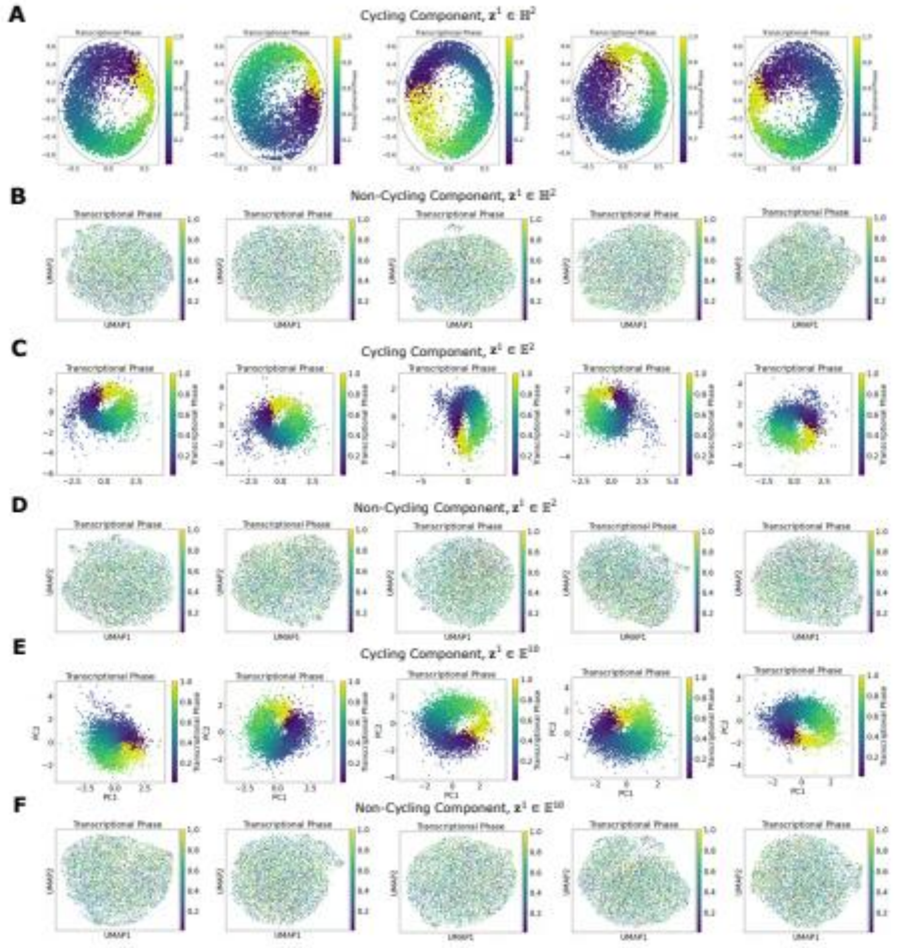

Figure R2: **Using the hyperbolic space or the Euclidean space to capture the cell cycle signal.** (**A**) The embeddings of the first component, $\mathbf{z}^1$, and (**B**) the embeddings of the second component, $\mathbf{z}^2$, when $\mathbf{z}^1 \in \mathbb{H}^2$ and $\mathbf{z}^2 \in \mathbb{E}^{10}$ for five different runs of CellUntangler. (**C**) The embeddings of the first component, $\mathbf{z}^1$, and (**D**) the embeddings of the second component, $\mathbf{z}^2$, when $\mathbf{z}^1 \in \mathbb{E}^2$ and $\mathbf{z}^2 \in \mathbb{E}^{10}$ for five different runs of CellUntangler. (**E**) The embeddings of the first component, $\mathbf{z}^1$, and (**F**) the embeddings of the second component, $\mathbf{z}^2$, when $\mathbf{z}^1 \in \mathbb{E}^{10}$ and $\mathbf{z} \in \mathbb{E}^{10}$ for five different runs of CellUntangler.

	$\mathbf{z}^1 \in \mathbb{H}^2$	$\mathbf{z}^1 \in \mathbb{E}^2$	$\mathbf{z}^1 \in \mathbb{E}^{10}$
Cell Cycle Time	0.805	**0.833**	0.807

Table R3: **Comparison of cell cycle reconstruction for the mESCs data.** The transcriptional phase output by DeepCycle[2], as analyzed by the original authors of this dataset is used as the ground truth. The average correlation over ten runs is reported for CellUntangler

However, while using the Euclidean space results in the cycling cells forming a circle overall, this is not the case for datasets with both cycling and non-cycling cells such as the immune cells dataset. When using the hyperbolic space with the RoWN to capture the cell cycle signal, $\mathbf{z}^1 \in \mathbb{E}^2$, we can recenter the cycling cells around the non-cycling cells (**Figure R3A**). Non-cell cycle specific signals like cell type were captured using the Euclidean space, $\mathbf{z}^2 \in \mathbb{E}^{10}$ (**Figure R3B**). Using the Euclidean space with dimension two, $\mathbf{z}^1 \in \mathbb{E}^2$, the cycling cells were only separated from the non-cycling cells (**Figure R3C**). Cell type was still captured in the non-cell cycle component, $\mathbf{z}^2 \in \mathbb{E}^{10}$ (**Figure R3D**). Similarly, when using $\mathbf{z}^1 \in \mathbb{E}^{10}$ and $\mathbf{z}^2 \in \mathbb{E}^{10}$, cycling cells only separated from non-cycling cells in $\mathbf{z}^1$ (**Figure R3E**), and cells separated by cell type in $\mathbf{z}^2$ (**Figure R3F**). We updated **Figure S3** to include a visualization of the cell cycle component using $\mathbf{z}^1 \in \mathbb{E}^2$ and $\mathbf{z}^2 \in \mathbb{E}^{10}$ from **Figure R3C** and **Figure R3E** to become panels **C** and **D**.

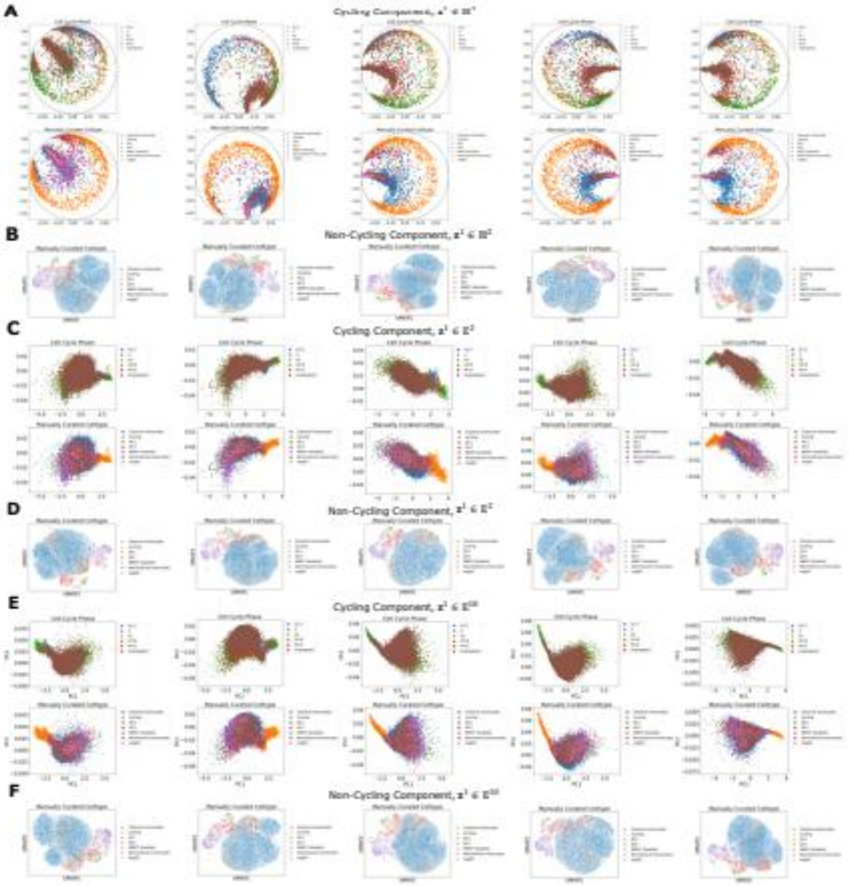

Figure R3: **Using the hyperbolic space or the Euclidean space to capture the cell cycle signal.** (**A**) The embeddings of the first component, $\mathbf{z}^1$, and (**B**) the embeddings of the second component, $\mathbf{z}^2$, when $\mathbf{z}^1 \in \mathbb{H}^2$ and $\mathbf{z}^2 \in \mathbb{E}^{10}$ for five different runs of CellUntangler. (**C**) The embeddings of the first component, $\mathbf{z}^1$, and (**D**) the embeddings of the second component, $\mathbf{z}^2$, when $\mathbf{z}^1 \in \mathbb{E}^2$ and $\mathbf{z}^2 \in \mathbb{E}^{10}$ for five different runs of CellUntangler. (**E**) The embeddings of the first component, $\mathbf{z}^1$ and (**F**) the embeddings of the second component, $\mathbf{z}^2$, when $\mathbf{z}^1 \in \mathbb{E}^{10}$ and $\mathbf{z}^2 \in \mathbb{E}^{10}$ for five different runs of CellUntangler.

2. The authors demonstrate the independence of latent subspaces through visualization (e.g., in Figure R2, z1 clusters cells by cell cycle phase while z2 clusters cells by genotype), but lack quantitative metrics. No Mutual Information (MI) or cosine similarity between z1 and z2 has

been calculated, making it impossible to objectively measure the "impact of the stop-gradient strategy on the degree of disentanglement." For instance, in the pancreatic dataset, after applying the stop-gradient strategy, is the Mutual Information between z1 (cell cycle) and z2 (differentiation trajectory) significantly lower than that without the stop-gradient strategy?

We thank the reviewer for their important question of providing an objective measurement of disentanglement. We measured the Mutual Information (MI)[3] between $\mathbf{z}^1$, the embeddings to capture a specific signal, and $\mathbf{z}^2$, the embeddings to capture other signals. E.g., $\mathbf{z}^1$ captures the cell cycle signal while $\mathbf{z}^2$ captures non-cell cycle specific signals. Using no stop-gradient tended to result in a lower MI between $\mathbf{z}^1$ and $\mathbf{z}^2$ across datasets (**Table R4**). However, we would like to clarify that good disentanglement does not necessarily mean that $\mathbf{z}^1$ and $\mathbf{z}^2$ are independent of each other. For example, for the pancreas dataset, the cycling cells consist of mostly ductal cells and low *Ngn3* EP cells, and thus $\mathbf{z}^1$ and $\mathbf{z}^2$ are not independent. In addition, cells of different types or states may inappropriately occupy a cell cycle stage, which could also lead to dependencies between $\mathbf{z}^1$ and $\mathbf{z}^2$. E.g., for the mouse pancreas dataset, without stop-gradient, the differentiation trajectory in $\mathbf{z}^2$ may not be captured as clearly (**Figure R4A**) compared to the differentiation trajectory in $\mathbf{z}^2$ with stop-gradient (**Figure R4B**). The MI was lower though between $\mathbf{z}^1$ and $\mathbf{z}^2$ without stop-gradient.

	No Stop-Gradient	Stop-Gradient
HeLa	0.122	0.206
mESCs	0.186	0.503
Immune cells	0.369	0.364
Mouse pancreas	0.876	1.174
Mouse liver cells	0.261	0.423

Table R4: The Mutual Information between $\mathbf{z}^1$ and $\mathbf{z}^2$ across different datasets. The average Mutual Information over ten runs is reported for CellUntangler.

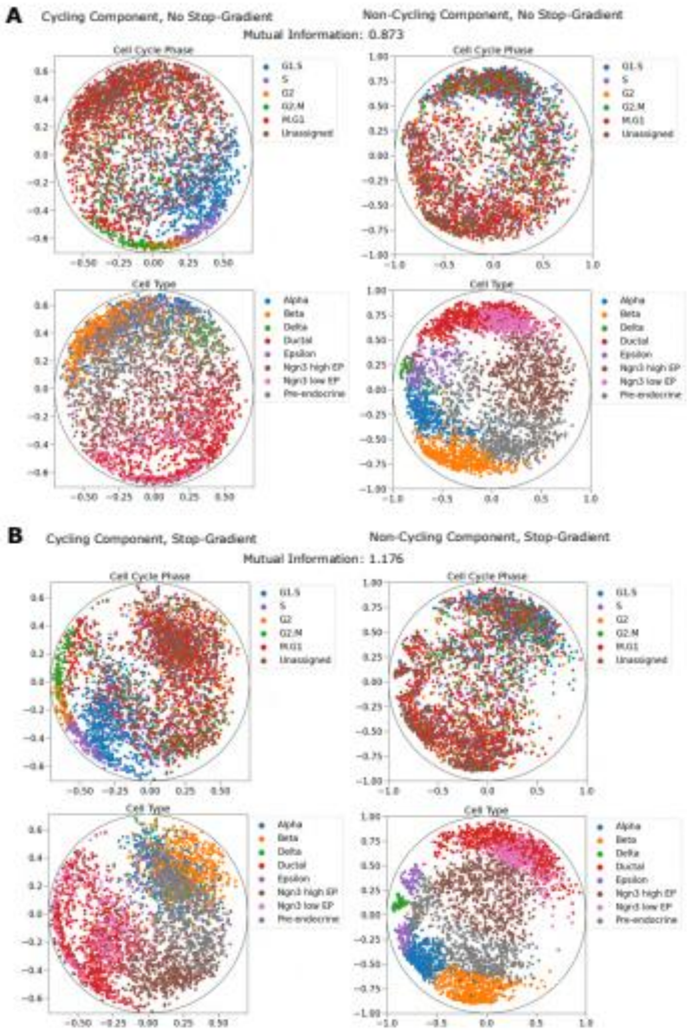

Figure R4: **The Mutual Information between embeddings without stop-gradient and with stop-gradient.** (**A**) Visualization of $\mathbf{z}^1$ and $\mathbf{z}^2$ without stop-gradient and (**B**) with stop-gradient. The Mutual Information is between $\mathbf{z}^1$ and $\mathbf{z}^2$.

We thus also measured the MI between the embeddings and the labels for the different signals in each dataset. E.g., we measured the MI between $\mathbf{z}^1$ and the cell cycle phase labels. For the mouse pancreas dataset, the MI was higher between $\mathbf{z}^1$ and the cell cycle phase when using the cycling cells only since the cell cycle phase assigned to the non-cycling cells by

Revelio could be incorrect (**Table R5**). The MI between the embeddings and the signal they should capture was higher when using stop-gradient. The conditional MI of $\mathbf{z}^2$ and the cell type labels given $\mathbf{z}^2$, MI($\mathbf{z}^1$;cell type | $\mathbf{z}^2$), was closer to 0 using stop-gradient suggesting that $\mathbf{z}^2$ is more informative of the differentiation trajectory when using stop-gradient. The k-NN accuracies on cell type using $\mathbf{z}^2$ were higher when using stop-gradient as well (**Figure R5**).

	No Stop-Gradient	Stop-Gradient
MI($\mathbf{z}^1$;**cell cycle phase**)	0.383	0.412
MI($\mathbf{z}^1_{cycling}$;**cell cycle phase**)	0.669	0.697
MI($\mathbf{z}^1$;cell type)	0.840	0.947
MI($\mathbf{z}^2$;cell cycle phase)	0.174	0.180
MI($\mathbf{z}^2_{cycling}$;cell cycle phase)	0.0722	0.0831
MI($\mathbf{z}^2$;**cell type**)	1.514	1.612
MI($\mathbf{z}^1$;cell type | $\mathbf{z}^2$)	0.142	0.0416

Table R5: The Mutual Information between embeddings and signals for the mouse pancreas dataset. The signal $\mathbf{z}^j$ should capture is bolded. $\mathbf{z}^1$ and $\mathbf{z}^2$ are the embeddings for all cells. $\mathbf{z}^1_{cycling}$ and $\mathbf{z}^2_{cycling}$ are the embeddings for the ductal and low *Ngn3* EP cells only. The average Mutual Information over ten runs is reported for CellUntangler.

A

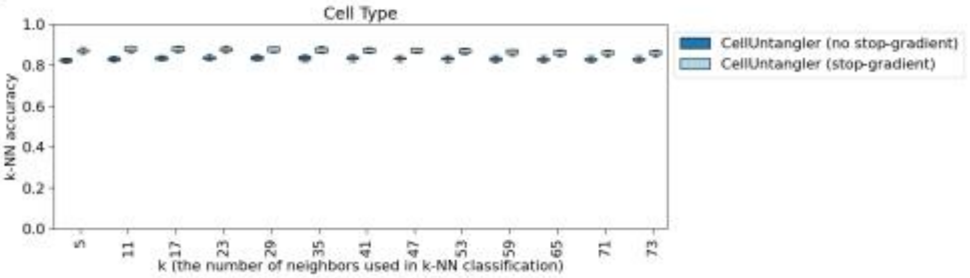

Figure R5: The k-NN accuracies on cell type for the mouse pancreas dataset. (**A**) The k-NN accuracies on cell type for the mouse pancreas dataset using $\mathbf{z}^2$. Boxplots depict the medians and the interquartile ranges (IQRs). The whiskers are the lowest datum still within 1.5 IQR of the lower quartile and the highest datum still within 1.5 IQR of the upper quartile. Individual points below and above the whiskers indicate outliers.

However, for the HeLa dataset (**Table R6**) and immune cells dataset (**Table R7**), the MI between the embeddings and the signal they should capture did not increase as much when using stop-gradient compared to the mouse pancreas dataset. For the mouse liver dataset, the MI between $\mathbf{z}^1$ and the spatial layers was lower when using stop-gradient (**Table R8**).

We did not measure the MI between the embeddings and the labels for the mESCs dataset since we do not have discrete ground truth labels. As stop-gradient did not always lead to better results and tended to increase the MI between $\mathbf{z}^1$ and $\mathbf{z}^2$, this confirmed our default setting of not using stop-gradient for CellUntangler.

	No Stop-Gradient	Stop-Gradient
MI($\mathbf{z}^1$;**cell cycle phase**)	0.888	0.900
MI($\mathbf{z}^1$;batch)	0.012	0.009
MI($\mathbf{z}^2$;cell cycle phase)	0.096	0.096
MI($\mathbf{z}^2$;**batch**)	0.483	0.517

Table R6: **The Mutual Information between embeddings and signals for the HeLa dataset.** The signal $\mathbf{z}^j$ should capture is bolded. The average Mutual Information over ten runs is reported for CellUntangler.

	No Stop-Gradient	Stop-Gradient
MI($\mathbf{z}^1$;**cell cycle phase**)	0.103	0.0987
MI($\mathbf{z}^1_{cycling}$;**cell cycle phase**)	0.707	0.708
MI($\mathbf{z}^1$;cell type)	0.308	0.298
MI($\mathbf{z}^2$;cell cycle phase)	0.162	0.161
MI($\mathbf{z}^2_{cycling}$;cell cycle phase)	0.151	0.147
MI($\mathbf{z}^2$;**cell type**)	0.757	0.765

Table R7: **The Mutual Information between embeddings and signals for the immune cells dataset.** The signal $\mathbf{z}^j$ should capture is bolded. $\mathbf{z}^1$ and $\mathbf{z}^2$ are the embeddings for all the cells. $\mathbf{z}^1_{cycling}$ and $\mathbf{z}^2_{cycling}$ are the embeddings for the cycling cells only. The average Mutual Information over ten runs is reported for CellUntangler

	No Stop-Gradient	Stop-Gradient
MI($\mathbf{z}^1$;**layer**)	0.572	0.541
MI($\mathbf{z}^1$;ZT)	0.148	0.254
MI($\mathbf{z}^2$;layer)	0.0495	0.0840
MI($\mathbf{z}^2$;**ZT**)	1.148	1.160

Table R8: **The Mutual Information between embeddings and signals for the mouse liver dataset.** The signal $\mathbf{z}^j$ should capture is bolded. The average Mutual Information over ten runs is reported for CellUntangler.

Regarding the cosine similarity, it requires to compute the dot product between the embeddings $\mathbf{z}^1$ and $\mathbf{z}^2$ of a cell. Thus, both $\mathbf{z}^1$ and $\mathbf{z}^2$ should be of the same dimensionality, which is typically not true. Even if they are of the same dimensionality, this metric could be inappropriate for aessessing the disentanglement between $\mathbf{z}^1$ and $\mathbf{z}^2$. E.g., for the mouse pancreas

dataset, $\mathbf{z}^1 \in \mathbb{H}^2$ captured the cell cycle and $\mathbf{z}^2 \in \mathbb{H}^2$ captured the differentiation trajectory resulting a low cosine similarity (**Figure R6A**). However, when we moved the cells in $\mathbf{z}^2$ so that the same cell types are at similar regions in $\mathbf{z}^1$ and $\mathbf{z}^2$, the cosine similarity became higher (**Figure R6B**). The cosine similarity became lower again when cell types were placed at different regions in $\mathbf{z}^1$ and $\mathbf{z}^2$ (**Figure R6C**). Placing the differentiation trajectory in $\mathbf{z}^2$ at the same orientation as the cells in $\mathbf{z}^1$ though should not result in a worse performance score.

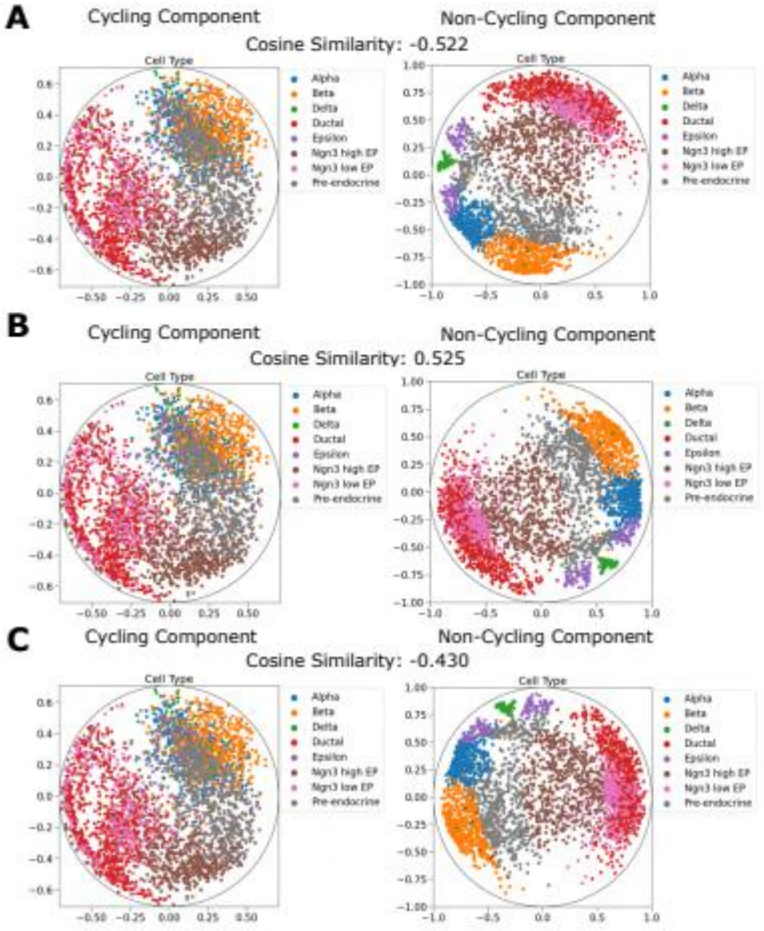

Figure R6: **The cosine similarity for the mouse pancreas dataset.** (**A**) Visualization of $\mathbf{z}^1$ and $\mathbf{z}^2$ without rotation, (**B**) when $\mathbf{z}^2$ is rotated 220°, and (**C**) when $\mathbf{z}^2$ is rotated 60°.

3. The paper demonstrates a variant of the VAE framework for signal decomposition with single-cell data. The latest deep generative models, scCross (PMID: 39075536) and scGlue (PMID: 35501393), are currently available. It is recommended that the author compare your model with these two methods.

We thank the reviewer for suggesting a comparison with scGlue[4] and scCross[5]. These tools are designed for single-cell multiomics data integration and require different omics layers as input. For example, they may be used to integrate chromatin accessibility (single-cell ATAC-sequencing), DNA methylation (snmC-seq, sci-MET), and single-cell RNA sequencing data. In contrast, CellUntangler is currently tailored to disentangle biological signals within scRNA-seq data only and is not applicable for across-modality integration. Given the distinct inputs and outputs, we believe a direct comparison is beyond the scope of this study. Nevertheless, we cite these tools in the **Limitations of the Study** section and note that extending CellUntangler to multi-omics data analysis is a potential future direction.

References

1. Schwabe, D., Formichetti, S., Junker, J. P., Falcke, M. & Rajewsky, N. The transcriptome dynamics of single cells during the cell cycle. *Molecular Systems Biology* **16**, e9946 (2020).
2. Riba, A. *et al.* Cell cycle gene regulation dynamics revealed by rna velocity and deep-learning. *Nature Communications* **13**, 2865 (2022).
3. Wally, Y. *et al.* Flatland and beyond: Mutual information across geometries. In *2nd Beyond Euclidean Workshop: Hyperbolic and Hyperspherical Learning for Computer Vision* (2025).
4. Cao, Z.-J. & Gao, G. Multi-omics single-cell data integration and regulatory inference with graph-linked embedding. *Nature Biotechnology* **40**, 1458–1466 (2022).
5. Yang, X., Mann, K. K., Wu, H. & Ding, J. scCross: a deep generative model for unifying single-cell multi-omics with seamless integration, cross-modal generation, and in silico exploration. *Genome Biology* **25**, 198 (2024).
